# Cross-Linking Cellular Prion Protein Induces Neuronal Type 2-Like Hypersensitivity

**DOI:** 10.3389/fimmu.2021.639008

**Published:** 2021-07-30

**Authors:** Utpal Kumar Adhikari, Elif Sakiz, Xian Zhou, Umma Habiba, Sachin Kumar, Meena Mikhael, Matteo Senesi, Chun Guang Li, Gilles J. Guillemin, Lezanne Ooi, Monique Antoinette David, Steven Collins, Tim Karl, Mourad Tayebi

**Affiliations:** ^1^School of Medicine, Western Sydney University, Campbelltown, NSW, Australia; ^2^National Institute of Complementary Medicine (NICM) Health Research Institute, Western Sydney University, Campbelltown, NSW, Australia; ^3^Australian National Creutzfeldt-Jakob Disease Registry, The Florey Institute of Neuroscience and Mental Health, The University of Melbourne, Parkville, VIC, Australia; ^4^Department of Medicine, Royal Melbourne Hospital, The University of Melbourne, Parkville, VIC, Australia; ^5^Department of Biomedical Sciences, Faculty of Medicine and Health Sciences, Macquarie University, Wollongong, NSW, Australia; ^6^School of Chemistry and Molecular Bioscience, Illawarra Health and Medical Research Institute, Wollongong, NSW, Australia; ^7^School of Chemistry and Molecular Bioscience, University of Wollongong, Wollongong, NSW, Australia; ^8^Neuroscience Research Australia (NeuRA), Sydney, NSW, Australia; ^9^School of Medical Sciences, University of New South Wales, Sydney, NSW, Australia

**Keywords:** anti-PrP antibodies, cellular prion protein, neurotoxicity, allergenicity, mouse primary neurons, neuroblastoma cell line, microglia cell line

## Abstract

**Background:**

Previous reports identified proteins associated with ‘apoptosis’ following cross-linking PrP^C^ with motif-specific anti-PrP antibodies *in vivo* and *in vitro*. The molecular mechanisms underlying this IgG-mediated neurotoxicity and the role of the activated proteins in the apoptotic pathways leading to neuronal death has not been properly defined. Previous reports implicated a number of proteins, including apolipoprotein E, cytoplasmic phospholipase A2, prostaglandin and calpain with anti-PrP antibody-mediated ‘apoptosis’, however, these proteins are also known to play an important role in allergy. In this study, we investigated whether cross-linking PrP^C^ with anti-PrP antibodies stimulates a neuronal allergenic response.

**Methods:**

Initially, we predicted the allergenicity of the epitope sequences associated with ‘neurotoxic’ anti-PrP antibodies using allergenicity prediction servers. We then investigated whether anti-PrP antibody treatment of mouse primary neurons (MPN), neuroblastoma cells (N2a) and microglia (N11) cell lines lead to a neuronal allergenic response.

**Results:**

*In-Silico* studies showed that both tail- and globular-epitopes were allergenic. Specifically, binding regions that contain epitopes for previously reported ‘neurotoxic’ antibodies such as ICSM18 (146-159), ICSM35 (91-110), POM 1 (138-147) and POM 3 (95-100) lead to activation of allergenic related proteins. Following direct application of anti-PrP^C^ antibodies on N2a cells, we identified 4 neuronal allergenic-related proteins when compared with untreated cells. Furthermore, we identified 8 neuronal allergenic-related proteins following treatment of N11 cells with anti-PrP^C^ antibodies prior to co-culture with N2a cells when compared with untreated cells. Antibody treatment of MPN or MPN co-cultured with antibody-treated N11 led to identifying 10 and 7 allergenic-related proteins when compared with untreated cells. However, comparison with 3F4 antibody treatment revealed 5 and 4 allergenic-related proteins respectively. Of importance, we showed that the allergenic effects triggered by the anti-PrP antibodies were more potent when antibody-treated microglia were co-cultured with the neuroblastoma cell line. Finally, co-culture of N2a or MPN with N11-treated with anti-PrP antibodies resulted in significant accumulation of NO and IL6 but not TNF-α in the cell culture media supernatant.

**Conclusions:**

This study showed for the first time that anti-PrP antibody binding to PrP^C^ triggers a neuronal hypersensitivity response and highlights the important role of microglia in triggering an IgG-mediated neuronal hypersensitivity response. Moreover, this study provides an important impetus for including allergenic assessment of therapeutic antibodies for neurodegenerative disorders to derive safe and targeted biotherapeutics.

## Introduction

Prion diseases or transmissible spongiform encephalopathies (TSE) are invariably fatal diseases characterized by loss of motor control, dementia and paralysis (1, 2). These disorders are caused by the conversion of a transmembrane cellular prion protein (PrP^C^) into a misfolded form (PrP^Sc^) (3, 4). PrP^C^ is a soluble protein rich in alpha helical content while PrP^Sc^ is rich in β-pleated sheets and characterised by its insolubility in detergents and partial resistance to proteases (4–7). The function of PrP^C^ has not been completely elucidated but due its conserved nature in a wide range of species, it is believed to play key and vital roles in maintaining cell homeostasis. However, PrP^C^ was implicated in cell activation, proliferation and differentiation (8–10), copper binding (11), synaptic plasticity and signal transduction (12–15). Prion diseases immunotherapeutics that directly target PrP^Sc^ elimination and transient inhibition of PrP^C^ have been efficacious in rodent models (16–20). However, several reports highlighted potential side-effects caused by anti-PrP^C^ antibody treatment *in vitro* and *in vivo* (12, 15, 21–25). Of note, the antibody-mediated ‘neurotoxic’ effects reported previously were made on the basis of microscopic assessments following TUNEL and/or standard histological staining but have not been characterized at a molecular level (23, 24, 26), with the exception of reports by Tayebi et al, Sonati et al. and Goniotaki et al. that confirmed the association of apolipoprotein E (APOE), cytoplasmic phospholipase A2 (cPLA2), prostaglandin (PG), calpain (CAPN) and group-I metabotropic glutamate receptors (mGluR1 and mGluR5) with anti-PrP mediated ‘neurotoxicity’ (21, 27, 28). In fact, these proteins are known to play a key role in allergic reactions, and most were identified as human allergy-related proteins by the AllerGAtlas database. For instance, levels of APOE in the bronchoalveolar fluid derived from patients with hypersensitivity pneumonitis were shown to be significantly high and APOE was suggested to play an important role in this allergic disease (29). Moreover, impaired delayed type hypersensitivity responses were observed in APOE-null mice, demonstrating the important role of APOE in regulating allergy (30). The role of cPLA2 in allergic response was characterized (31). Uozumi and colleagues showed that cPLA2-deficient mice displayed marked decrease in the synthesis of eicosanoids (including PG) and platelet activating factor and that the anaphylactic responses were significantly affected. Moreover, cPLA2 was also shown to be essential for fast eicosanoid generation (including PG) by providing arachidonic acid (32). PGs are synthesized by the cyclooxygenase (COX) enzymes in the arachidonic acid metabolic pathway and have been shown to play an important role in hypersensitivity (33). The neuron-expressed mGluR7 was shown to regulate histamine and ablation of mGluR7 in mice led to anaphylaxis (34). Of importance, mGluR7-interactors; mGluR1 and mGluR5 were shown to form complexes with PrP^C^ and their pharmacological inhibition cancelled the ‘neurotoxic’ effects caused by anti-PrP antibodies (28). Of interest, the anti-histaminic drug astemizole, a second generation H1-receptor antagonist, was shown to extend the survival of mice infected with prions (35), suggesting a connection between PrP and histamine.

Microglia activation is an important neuropathological feature associated with neurodegenerative disorders, including prion diseases (36–38). It was previously reported that the cytotoxic effects triggered by a putative toxic PrP peptide were intimately linked to microglia activation (36, 39). Moreover, a report by Lefebvre-Roque et al. demonstrated that a 2-week intraventricular infusion of a full length anti-PrP^C^ antibody or with its F(ab’)2 and Fab fragment derivative to wild-type and prion-infected mice initiated at the beginning of prion neuroinvasion led to microglial recruitment/activation (25). Interestingly, the authors show that microglial activation associated with neuronal death was only observed after injection of anti−PrP antibodies and did not seem to be related to prion infection.

In this study, we aimed to verify whether direct treatment of neurons with anti-PrP antibodies or following co-culture with anti-PrP antibody-treated microglia leads to a neuronal hypersensitive response. In order to investigate the molecular mechanisms underlying antibody-induced toxicity, mass spectrometry analysis was performed to identify allergenic proteins and to characterize possible pathways leading to *IgG-Mediated Neuronal Hypersensitivity* post-antibody treatment. Here, we used a set of anti-PrP antibodies, including ICSM (40), SAF (41, 42), and POM antibodies (43) with binding specificity for epitopes located on the globular domain (GD) or flexible tail (FT) of PrP^C^. We show that direct application of anti-PrP antibodies (DAT) on N2a induces a neuronal allergenic reaction by activating 4 allergenic-related proteins. Co-culture of N2a with antibody treated N11 (DMT) led to a more extensive alteration of the proteome and identified 8 allergenic-related proteins. Furthermore, DAT and DMT of mouse primary neurons (MPN) revealed 10 and 7 allergenic-related proteins respectively when compared with untreated cells; and 5 and 4 allergenic-related proteins when compared with treatment with a hamster/human specific anti-PrP 3F4 antibody. Of importance, we show that DMT with some of these anti-PrP antibodies lead to a substantial release of proinflammatory cytokine Interleukin 6 (IL6) but not Tumour Necrosis Factor alpha (TNF-α) confirming a possible role of anti-PrP antibodies in triggering a type 2-like hypersensitive reaction (44). Finally, we show that FcεR1a was activated following treatment with anti-PrP antibodies. This study demonstrates and for the first time that cross-linking PrP^C^ with anti-PrP antibodies leads to a neuronal allergic reaction and also highlights the crucial role played by microglia in this *IgG-Mediated Neuronal Type-2 like Hypersensitive reaction*.

## Methods

**Figure 1:**
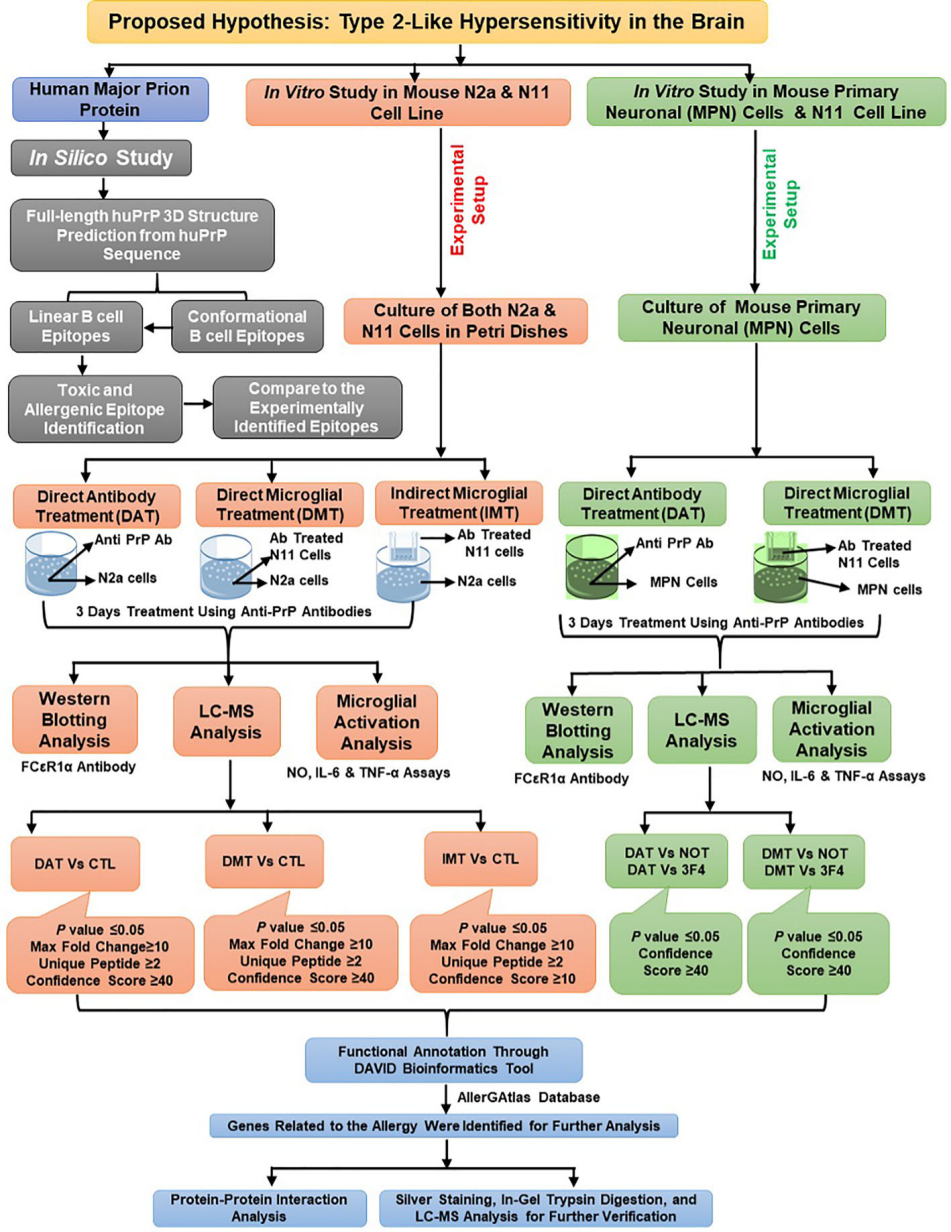
Overall workflow of the *in silico* and *in vitro* study for the identification of allergenic proteins following antibody treatment and LC-MS analysis.

The overall methods of the *in-silico* study, *in vitro* experimental setup, western blotting, immunofluorescence study and liquid-chromatography mass-spectrophotometry (LC-MS) analysis are illustrated in **Figure 1**. Methods related to the predictions of the 3-dimensional structure of the human PrP^C^; of linear and conformational B cell epitopes; and of the toxicity and allergenicity of the linear B cell epitopes are described in extended methods.

### Treatment of Neurons and Microglia Cell Line With Anti-PrP Antibodies

We used a mouse neuroblastoma (N2a) (American Type Culture Collection, ATCC, USA) (45), mouse primary neurons (MPN) derived from eight week-old wild-type (WT) mice (C57BL/6 x SV129 background) harvested from the brain’s sub ventricular zone (SVZ) as described (46), and a mouse microglia (N11) (47) cell line to investigate the effects of anti-PrP antibodies. The N2a cells were used to assess hypersensitivity following direct application of anti-PrP antibodies (DAT). The N11 cells, initially treated with anti-PrP antibodies, were used to assess their effects on N2a or MPNs following direct co-culture (DMT) or after separating the antibody treated N11 and N2a cells by a tissue culture insert (IMT).

#### Direct Antibody Treatment (DAT)

N2a cells were plated on 24 tissue culture well plates (Falcon, country) at 200,000 cells/well for 48 hours in tissue culture medium [Dulbecco’s Modified Eagle Medium (DMEM) (Thermo Fisher Scientific, Australia), 10% fetal bovine serum (FBS) (Gibco, Fisher Scientific, Australia), and 1% Penicillin-streptomycin (Sigma, USA)] at 37°C in 5% CO_2_ until optimum growth and adhesion to the surface of the plates were observed. The medium was changed daily. After 48 hours, 3μg of different anti-PrP antibodies, including ICSM18 (40), ICSM35 (40), POM1 (43), POM2 (43), POM3 (43), SAF32 (42), or SAF70 (41) were added daily to the N2a cultures for 3 days. The cells were then removed from the plates and centrifuged at 800 rpm for 5 minutes. The cells were lysed with NP-40 lysis buffer (150mM NaCl, 1.0% Nonidet P-40 and Triton X-100, 50 mM Tris-Cl, adjust PH to 7.4) with addition of AEBSF protease inhibitor (Sigma, USA) and stored at −80°C until further use.

Undifferentiated WT MPN were plated in 24 well plate at 50,000 cells/well in NeuroCult™ Differentiation kit (Stem Cell Technologies, Canada) and incubated at 37°C in 5% CO_2_ for 7 days. The cells were then treated daily with 1μg of ICSM18, ICSM35 (40) or 3F4 (Sigma-Aldrich, Australia) for 3 days. Cells were then trypsin-removed from the plates, centrifuged at 800 rpm for 5 minutes and lysed with NP-40 lysis buffer (150mM NaCl, 1.0% Nonidet P-40 and Triton X-100, 50 mM Tris-Cl, adjust PH to 7.4) with addition of AEBSF protease inhibitor (Sigma, USA) and stored at −80°C until further use.

#### Direct Microglia Treatment (DMT)

For co-culture with N2a cells, the N11 cells were plated and cultured on a Petri dish at 200,000 cells/well for 48 hours in culture medium and incubated at 37°C in 5% CO_2_. N11 cells were then treated with 3μg of different anti-PrP antibodies as above daily for 3 days. The antibody treated N11 cells were centrifuged at 800 rpm for 5 minutes before co-culturing with confluent N2a cells for 3 days. Finally, the N2a/antibody-treated N11 co-culture was centrifuged at 800 rpm for 5 minutes and the pellet was lysed with NP-40 lysis buffer with addition of AEBSF protease inhibitor then stored at −80°C until further use. The supernatants were snap frozen in liquid nitrogen and stored at −80°C until further use. In another experiment, the antibody-treated N11 cells were centrifuged at 800 rpm for 5 minutes and the pellet was lysed with NP-40 lysis buffer with addition of AEBSF protease inhibitor then stored at −80°C until further use. The supernatants were snap frozen in liquid nitrogen and stored at −80°C until further use.

For co-culture with MPN, the N11 cells were plated and cultured on a 24 well plate at 50,000 cells/well for 24 hours in culture medium (DMEM medium, 10% FBS, and 1% Penicillin-streptomycin) and incubated at 37°C in 5% CO_2_. The cells were then treated with 1μg of ICSM18, ICSM35 or 3F4 antibody as above. The antibody treated N11 cells were centrifuged at 800 rpm for 5 minutes before co-culturing with confluent MPN for 3 days. Finally, the WT MPN and antibody treated N11 co-culture was centrifuged at 800 rpm for 5 minutes and the pellet was lysed with NP-40 lysis buffer with addition of AEBSF protease inhibitor then stored at −80°C until further use. The supernatants were snap frozen in liquid nitrogen and stored at −80°C until further use.

#### Indirect Microglia Treatment (IMT)

In order to verify whether the potential allergenic effect is caused by molecules released from N11 cells following treatment with anti-PrP antibodies, the N11 cells were plated and cultured on tissue culture inserts (Nunc™ Polycarbonate Cell Culture Inserts, 0.4-micron pore size) in 24 well plate at 200,000 cells/well for 48 hours. The N11 cells were treated daily with 3μg of different anti-PrP antibodies as above. The tissue culture inserts containing antibody-treated N11cells were transferred to 24 well tissue culture plate containing confluent N2a cells and left for 3 days. Finally, the N2a cells were removed from the wells and centrifuged at 800 rpm for 5 minutes and lysed with NP-40 lysis buffer and AEBSF protease inhibitor before storing at −80°C until further use.

### Sample Preparation for Liquid Chromatography-Mass Spectrometry

#### In-Solution Trypsin Digestion

The cell lysates prepared above were used for Liquid Chromatography-Mass Spectrometry (LC-MS) sample preparation. For in-solution trypsin digestion, 100μl of protein sample (300 µg/mL cell lines or 170 µg/ml MPN) was concentrated using Rotational Vacuum Concentrator (Martin Christ Gefriertrocknungsanlagen GmbH, Germany). 6 µL DTT (Roche Diagnostics Deutschland GmbH, Germany) (200 mM DTT in Tris buffer, pH 7.8) was then added and the mixture was vortexed before addition of 30 µL of 6M Urea into the sample then incubated for 1h at room temperature. 6 μL iodoacetamide (Sigma-Aldrich, Australia) alkylating reagent (200 mM iodoacetamide in Tris buffer, pH 7.8) was then added, the sample mixture vortexed then followed by incubation for 1h at room temperature. The mixture was topped up with 225 µL of distilled water before adding 5 µL of trypsin (Promega Corporation, USA) solution and incubated overnight at 37°C. Finally, the reaction was stopped, and the pH of the solution adjusted to <6 with concentrated acetic acid. After trypsin digestion, the solution was purified using Solid Phase Extraction (SPE - Oasis HLB 1 cc Vac Cartridge, 30 mg) vacuum manifold (Waters Milford, Massachusetts, USA) then reconstituted in 15µL 0.1% formic acid, vortexed and kept for 30 minutes at 25°C. The solution was then vortexed and sonicated for 3 minutes then centrifuged at 14,000 rpm for 10 minutes before transferring into labelled glass vials.

#### In-Gel Trypsin Digestion

Cell lysates derived from ICSM35 or 3F4 antibody-treated MPN were run in a 12% SDS-PAGE gel (Bio-Rad, CA, USA) as above. Silver staining (ThermoFisher Scientific, Australia) was performed following the manufacturer’s instructions. Band were then cut out and used for in-gel trypsin digestion for final LC-MS analysis. Initially, 200 μL wash solution (50% acetonitrile, 50 mM ammonium bicarbonate) was added to the sample following continuous vortex for 10 minutes at room temperature. The solution was removed and repeated two times. 200 μL of acetonitrile was added to the gel, vortexed briefly and incubated at room temperature for 5 minutes. Acetonitrile was removed and residual amount was evaporated with a heated vacuum centrifugal concentrator for 5 minutes. 100 μL of the freshly prepared DTT solution (10 mM DTT in 50 mM ammonium bicarbonate) was added to the dried gel and incubated at 55°C for 1 hour. 100 μL of freshly prepared iodoacetamide solution (55 mM iodoacetamide in 50 mM ammonium bicarbonate) was then added to the gel then mixed by brief vertexing and incubated in the dark at room temperature for 45 minutes. 200 μL of wash solution was added to the sample following continuous vertexing for 10 minutes at room temperature. The wash solution was removed and repeated two times. 200 μL of acetonitrile was added to the gel, vortexed briefly and incubated at room temperature for 5 minutes. Acetonitrile was removed and discarded, and the residual acetonitrile was evaporated with a heated vacuum centrifugal concentrator for 5 minutes. 15 μL of 20 ng/μL trypsin solution (Promega Corporation, USA) (in 50 mM ammonium bicarbonate) was added to the dried gel core following 1 hour incubation at 30°C for rehydrating the gel. Sufficient amount of digestion buffer (50 mM ammonium bicarbonate/10% acetonitrile) was added to the gel and incubated at 37°C overnight. 50 μL of ultrapure (≥18 MΩ) H_2_O was added to the in-gel digestion mixture following continuous vertexing for 10 minutes at room temperature. The supernatant was removed and collected in a fresh, non-stick micro centrifuge tube prior to adding 50 μL of 50% acetonitrile/5% formic acid (v/v) and incubated the tube for 60 minutes at room temperature with frequent vortexing. The solution was collected after spinning the tube briefly in a micro centrifuge. Finally, the volume of the pooled solution was concentrated using a centrifugal concentrator at room temperature until the appropriate volume (~20 μL) is reached. The samples were then transferred into glass vial and ready for LS-MS analysis.

### Liquid Chromatography-Mass Spectrometry Analysis

The samples prepared above were carefully placed in a Waters Total Recovery chromatography sample vials for analysis. System specific cleaning protocol was run before loading the sample to avoid contamination in the system. LC-MS was performed using a Waters nanoAcquity UPLC equipped with a Waters nanoEase M/Z Peptide BEH C18 Column, 130Å, 1.7 µm 75 um x 100 mm, thermostatted to 40°C (Waters Corporation, USA). Briefly, solvent A consisted of ultrapure water (Milli-Q) plus 0.1% formic acid and solvent B consisted of LC-MS grade acetonitrile (Burdick and Jackson) plus 0.1% formic acid. Samples were injected onto a trapping column (Waters nanoEase M/Z Symmetry C18 Trap Column, 100A, 5 µm, 180 µm x 20mm) at 5 uL/min at 99% Solvent A for 3 min before being eluted on the Analytical Column with a flowrate of 0.30 uL/min. An initial solvent composition of 1% B was ramped to 85% B over 50 minutes. Injections of 1 μL were made from sample solutions stored at 4°C.

Mass spectrometry was performed using a Waters SYNAPT G2-Si (HDMS) spectrometer fitted with a nano electrospray ionization source and operating in positive ion mode. Mass accuracy was maintained by infusing at 0.5 μL/min a lock spray solution of 1 pg/μL leucine encephalin in 50% aqueous acetonitrile, plus 0.1% formic acid, calibrated against a sodium iodide solution. The capillary voltage was maintained at 3 kV, cone voltage at 30 V, source offset at 30 V, ion block temperature 80°C, gas (N2) flows: purge gas 20 L/hr., cone gas 20 L/hr. MassLynx Mass Spectrometry Software (Waters Corporation, USA) was used to process the data. Each sample was run for three times in the LC-MS system and finally the collected data were run against the mouse proteome using Uniprot database and analysed using Progenesis QI software (Waters Corporation, USA).

### Identification of the Allergy Related Genes

The final dataset from LC-MS was checked to find out whether there is any allergy related genes or not in our identified gene list. We used AllerGAtlas 1.0 (http://biokb.ncpsb.org/AlleRGatlas/), a human allergy-related genes database which has been developed based on the 1195 well-annotated human allergy-related genes, determined by text-mining and manual curation (48). The objectives of developing this AllerGAtlas database was to look on the pathogenesis and epidemiology of individual cases, novel diagnostic and prognostic biomarker, individual treatment responses and precision medicine (48).

### Western Blot Analysis

Cell lysates derived from antibody-treated cell lines and MPN was mixed with an equal volume of Laemmli buffer (Bio-Rad, CA, USA). The solution was vortexed then heated for 5 min to 95°C. The solution was left to cool down before loading the sample into 12% SDS-PAGE gel (Bio-Rad, CA, USA) and run at 200 Volt for 5 min then 1h 30 min at 100V in running buffer (Bio-Rad, CA, USA). Following transfer at 18V for 2h 30 min in transfer buffer (Bio-Rad, CA, USA), the membranes were blocked using 2% bovine serum albumin (BSA) (Sigma-Aldrich, USA) or 5% skimmed milk followed by human TrueStain FC_x_™ blocker (Biolegend, San Diego, USA) (5µl/blot). The blots were rinsed with TBST and 0.5 µg/ml of primary antibody mouse anti-human FcϵRIα (Biolegend, San Diego, USA) was added for overnight incubation before washing with 0.1% TBST buffer. The secondary antibody goat anti-mouse IgG (Fab specific) (1:80000) (Sigma-Aldrich, USA) was then added for 1hour at room temperature. The blot was washed using 0.1% TBST then visualized using the Clarity Western ECL Substrate (Bio-Rad, CA, USA) in iBright™ CL1000 Imaging System (Thermo Fisher Scientific).

### Nitric Oxide, TNFα and IL-6 assays

Nitric oxide (NO) production was measured in all antibody-treated cells and N11 cells by Griess reaction. Cell supernatants were collected and mixed with Griess reagent (1% sulfanilamide in 5% phosphoric acid and 0.1% N-1-naphthylethylenediamine dihydrochloride in Milli-Q water) for NO measurement in a microplate which was monitored under 540 nm using a microplate reader (BMG CLARIOstar, Victoria, Australia).

Supernatants derived from anti-PrP-treated cells were also collected and analysed for IL-6 and TNF-α synthesis using commercial ELISA kits (IL6: Biolegend, Australia; TNF: Peprotech, Australia) according to the manufacturer’s instructions.

### Statistical Analyses

Statistical analyses were assessed using GraphPad Prism 9 with One-way ANNOVA followed by Tukey’s multiple comparisons test method. The results were considered significant at *p*<0.05.

## Results

### Identification of Allergenic B Cell Epitopes in the Three-Dimensional Structure of the Human Major Prion Protein

**Figure 2:**
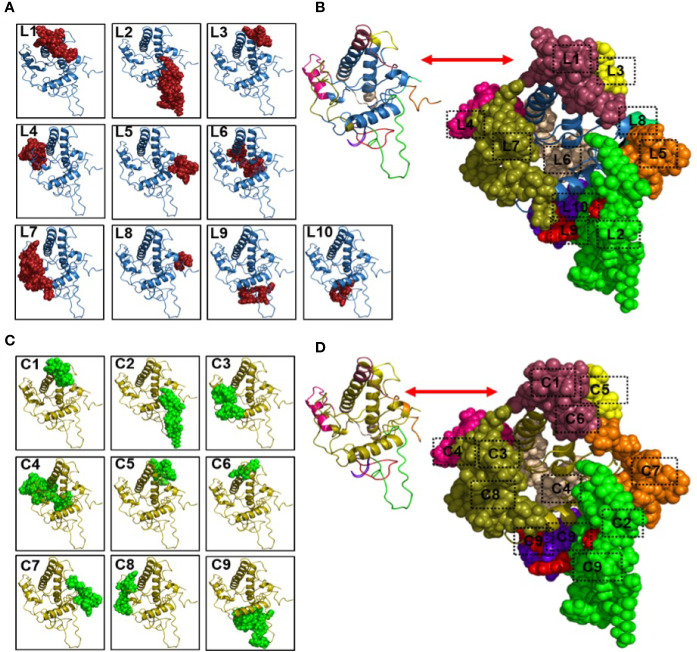
Predicted linear and conformational B cell epitopes and their position in the human major prion protein structure. Here, the protein structures have shown as cartoon structure and the linear and conformational B cell epitopes have shown as spheres on the protein structures. **(A)** Linear B cell epitopes where L1 (222-238), L2 (89-111), L3 (167-174), L4 (137-153), L5 (245-253), L6 (189-204), L7 (24-49), L8 (1-4), L9 (56-66), and L10 (76-86) representing different epitope position. **(B)** all the linear B cell epitopes have been shown as spheres by different colours to see the overlaps of the identified linear B cell epitopes. **(C)** Conformational B cell epitopes where C1(227-235), C2 (100-112), C3 (25-38), C4 (137-149, 151-153, 157, 189, 191-204), C5 (164, 168-174, 177), C6 (222-225), C7 (236-238, 242, 245-253), C8 (39-49), and C9 (55-67, 75-86, 89-99) representing different epitope position. **(D)** all the conformational B cell epitopes have been shown as spheres by different colours to see the overlaps of the identified conformational B cell epitopes. Here, liner B cell epitope L1 was found in the conformational B cell epitope position C1 and C6; C2 and C9 representing the linear epitope L2; C5 representing the linear epitope L3; C4 (two position where one is in the left side and the other one is in the middle position of the structure) representing the linear epitope L4 and L6; C7 representing the linear epitope C5; C3 and C8 representing the linear epitope L7; and the conformational epitope C9 (two position in the bottom of structure) representing both the linear epitope L9 and L10.

Following modelling of the human major prion protein (huPrP) 3-dimensional structure (**Figure S1**), we predicted the linear B cell epitopes from the huPrP 3D structure and found 10 B cell linear epitopes ranging from 4 to 26 amino acids long with a protrusion index (PI) of 0.503 to 0.798 (**Table 1**) (49). The full-length huPrP is divided into three major parts, including the flexible tail (FT) region (23-123), which comprises the octa-peptide repeats (OR) region (50-90) and the globular domain (GD) regions (124-230). We found four epitopes (epitope L7: 24-49, epitope L9: 56-66 and epitope L10: 76-86, epitope L2: 89-111) in the FT region, two of them located in the OR region. The GD contained three epitopes (epitope L4: 137-153, epitope L3: 167-174 and epitope L6: 189-204). On the contrary, the short epitope 1-4 (epitope L8) is located in the N-terminal region (signal peptide region) and comparatively two longer epitopes L1:222-238 (both in GD and non-structured region) and L5:245-253 (non-structured region) are located in the C-terminal region of the full length huPrP (**Table 1**). The position of the B cell linear epitopes on the protein structure are illustrated in (**Figures 2A, B**). We also predicted 9 B cell conformational epitopes, shown in **Table 2**. The length of the B cell conformational epitopes ranged between 4 and 36 amino acid residues. The protrusion index value for the B cell conformational epitopes ranged between 0.55 and 0.883.

**Table 1:** Linear B cell epitopes and their toxicity and allergenicity from the three-dimensional structure of the human major prion protein.

Epitope No.	Position	Peptide	Number of Residues	Score	Allergenicity		Overall
					AllergenFP	AllerTop 2.0	
**L1**	222-238	SQAYYQRGSSMVLFSSP	17	0.798	Non-allergenic	Non-allergenic	Non-allergenic
**L2**	89-111	WGQGGGTHSQWNKPSKPKTNMKH	23	0.767	Non-allergenic	Allergenic	Allergenic
**L3**	167-174	DEYSNQNN	8	0.751	Allergenic	Non-allergenic	Allergenic
**L4**	137-153	PIIHFGSDYEDRYYREN	17	0.737	Allergenic	Non-allergenic	Allergenic
**L5**	245-253	SFLIFLIVG	9	0.701	Non-allergenic	Non-allergenic	Non-allergenic
**L6**	189-204	VTTTTKGENFTETDVK	16	0.685	Non-allergenic	Non-allergenic	Non-allergenic
**L7**	24-49	KRPKPGGWNTGGSRYPGQGSPGGNRY	26	0.676	Non-allergenic	Non-allergenic	Non-allergenic
**L8**	1-4	MANL	4	0.559	Allergenic	Allergenic	Allergenic
**L9**	56-66	GWGQPHGGGWG	11	0.541	Non-allergenic	Allergenic	Allergenic
**L10**	76-86	PHGGGWGQPHG	11	0.503	Non-allergenic	Allergenic	Allergenic

**Table 2:** Conformational B cell epitopes from the three-dimensional structure of the human major prion protein.

Epitope No.	Residues	Number of residues	Score
**C1**	Q227, R228, G229, S230, S231, M232, V233, L234, F235	9	0.883
**C2**	N100, K101, P102, S103, K104, P105, K106, T107, N108, M109, K110, H111, M112	13	0.811
**C3**	R25, P26, K27, P28, G29, G30, W31, N32, T33, G34, G35, S36, R37, Y38	14	0.747
**C4**	P137, I138, I139, H140, F141, G142, S143, D144, Y145, E146, D147, R148, Y149, R151, E152, N153, Y157, V189, T191, T192, T193, K194, G195, E196, N197, F198, T199, E200, T201, D202, V203, K204	32	0.717
**C5**	R164, E168, Y169, S170, N171, Q172, N173, N174, H177	9	0.708
**C6**	S222, Q223, A224, Y225	4	0.708
**C7**	S236, S237, P238, L242, S245, F246, L247, I248, F249, L250, I251, V252, G253	13	0.669
**C8**	P39, G40, Q41, G42, S43, P44, G45, G46, N47, R48, Y49	11	0.579
**C9**	G55, G56, W57, G58, Q59, P60, H61, G62, G63, G64, W65, G66, Q67, Q75, P76, H77, G78, G79, G80, W81, G82, Q83, P84, H85, G86, W89, G90, Q91, G92, G93, G94, T95, H96, S97, Q98, W99	36	0.55

The B cell linear epitope L2 overlapped with B cell conformational epitopes C2 and C9 (**Tables 1** and **2**). Further, linear epitope L4 also overlapped with conformational epitope C4 (**Tables 1** and **2**). On the other hand, rest of the other linear epitopes L1 overlapped with both C1 and C6; L3 overlapped with C5; L5 overlapped with C7; L6 overlapped with C4; L7 overlapped with both C3 and C8; and both L9 and L10 overlapped with C9 (**Figures 2C, D**).

In the *in silico* analysis, the prediction of the toxicity for each linear epitope was achieved with ToxinPred server (http://crdd.osdd.net/raghava/toxinpred/) using Support Vector Machine (SVM) and Quantitative Matrix (QM) method (50). The SVM method did not identify ‘toxic’ B cell linear epitopes from 3D structure however, the QM method identified epitopes L2 (89-111), L4 (137-153), L7 (24-49), L9 (56-66), and L10 (76-86) as being toxic. Of note, L2 and L4 epitope contain the binding sequences for the ‘neurotoxic’ anti-PrP antibodies ICSM35/POM3 antibodies (40, 43) and ICSM18/POM1 antibodies (40, 43), respectively. Interestingly, epitopes L9 (56-66) and L10 (76-86) are located in the octa-repeat region of the huPrP protein that contains the binding sequences for SAF 32 (59-89) (42) and POM2 (57-88) antibodies (43), respectively. We also predicted the allergenicity for the B cell linear epitopes using the AllergenFP (51) and AllerTop (52) allergenicity prediction server. L3 (167-174), L4 (137-153), and L8 (1-4) were predicted to be allergenic in AllergenFP, while AllerTop server identified epitopes L2 (89-111), L8 (1-4), L9 (56-66), and L10 (76-86) as allergenic. The allergenicity prediction results are shown in **Table 1**. Three linear epitopes were shown to be non-toxic and non-allergenic and included L1 (222-238) and L6 (189-204) on the globular domain and L5 (245-253) located on the non-structured region. We therefore investigated whether ICSM18, ICSM35, POM1, POM2, POM3, SAF32, and SAF70 antibodies trigger a neuronal allergenic reaction *in vitro*.

### Anti-PrP Antibodies Treatment Leads to Neuronal Type 2-Like Hypersensitivity *In Vitro*

**Figure 3:**
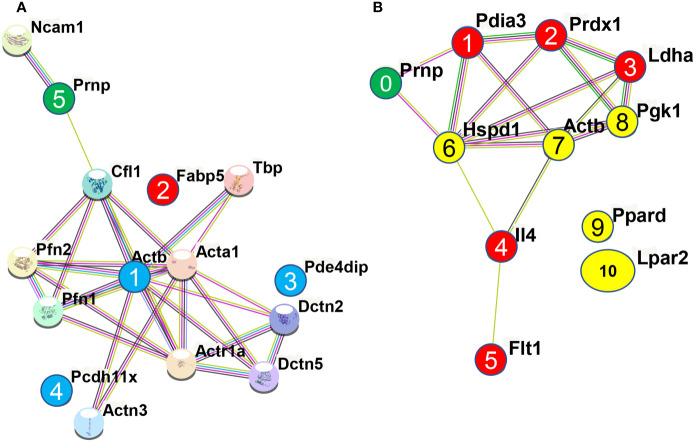
Protein-protein interaction of the identified allergenic genes following direct antibody treatment (DAT) of mouse neuroblastoma cells and mouse primary neuron (MPN). Protein-protein interaction analysis was performed using STRING v11.0 server. The *Mus Musculus* database was used as the host organism for the identification of protein interactions. **(A)** Protein-protein interaction of the identified allergenic genes in mouse neuroblastoma cells. Herein, interaction among the allergenic proteins (numbered 1, 2, 3, & 4) and with PrP^C^ (numbered 5) following direct antibody treatment. **(B)** Protein-protein interaction of the identified allergenic genes in mouse primary neuron (MPN). Herein, interaction among the allergenic proteins (numbered 1, 2, 3, 4, 5, 6, 7, 8, 9 & 10) and with PrP^C^ (numbered 0) following direct antibody treatment, where red and yellow color indicate the downregulated and upregulated genes, respectively and green color represents the cellular prion protein PrP^C^.

Treatment of N2a cells with ICSM18, ICSM35, POM1 and SAF70 led to the identification of 211 proteins (*p <*0.05) after LC-MS analysis when compared with untreated N2a cells. Out of the 211 proteins, only differentially expressed proteins were considered using maximum fold change ≥10, at least 2 identified unique peptides and a confidence score ≥ 40. The stringent parameters used here led to the identification of 26 proteins (**Table S1**). Of note, the parameters used to identify proteins associated with anti-PrP treatment are unusually high and would allow elimination of ‘false-negatives’ post LC-MS analysis. The 26 proteins were then assessed for allergenicity using AllergGAtlas database (http://biokb.ncpsb.org/AlleRGatlas/) (48) and 4 allergy related proteins, including beta-actin (ACTB), fatty acid-binding protein 5 (FABP5), protocadherin 11 (PCDH11X), and myomegalin (PDE4DIP) were identified. Among the 4 allergenic-related proteins, ACTB, PCDH11X and PDE4DIP were upregulated but FABP5 was found to be down regulated when compared to untreated control (**Table 3**). Protein-protein interaction of the identified 4 allergenic-related proteins showed that PrP networks with ACTB *via* Cofilin-1 (CFL1), while no direct interaction was observed for FABP5, PCDH11X and PDE4DIP (**Figure 3A**). Finally, analysis of individual anti-PrP antibody treatments revealed that PDE4DIP was present after DAT with ICSM18 and POM1 treatment, however, DAT with ICSM35 and SAF70 was not found to be associated with allergenic related proteins (**Table 4**).

**Table 3:** Properties of the identified allergenic proteins following direct antibody treatment (DAT) of the neuroblastoma cell line.

Accession	Gene Id	Protein Name	Anova (p)	Max Fold Change	Confidence Score	Peptides	Unique peptides	Highest Mean	Lowest Mean
**Q05816**	Fabp5	Fatty acid-binding protein 5	0.017	16	260	22	3	CTL	DAT
**B1AZR7**	Pcdh11X	Protocadherin 11	0.006	11.7	60.8	9	4	DAT	CTL
**Q3UBP6**	Actb	Uncharacterized protein	0.018	12.3	802	58	3	DAT	CTL
**Q3UR03**	Pde4dip	Myomegalin (Fragment)	0.02	29.9	49.1	7	3	DAT	CTL

The properties were identified by Progenesis Software after the LC-MS analysis.

**Table 4:** Identification of antibody-specific allergenic proteins following direct antibody treatment (DAT).

Gene ID	Accession	ICSM Antibodies				SAF Antibody		POM Antibody	
		ICSM18	CTL	ICSM35	CTL	SAF70	CTL	POM1	CTL
**Q05816**	Fabp5	-	-	-	-	-	-	-	-
**B1AZR7**	Pcdh11X	-	-	-	-	-	-	-	-
**Q3UBP6**	Actb	-	-	-	-	-	-	-	-
**Q3UR03**	Pde4dip	√	-	-	-	-	-	√	-

(√) Upregulated and (-) Downregulated.

Treatment of MPN with 1µg of ICSM18 or ICSM35 led to the identification of 135 proteins after LC-MS analysis when compared with untreated cells (**Table S2**). However, 73 proteins were identified when compared with MPN treated with 1µg of anti-PrP antibody 3F4 antibody (**Table S3**). Differentially expressed proteins (*p*< 0.05) were only considered with a confidence score ≥15 (**Tables S2, S3**). The 135 proteins (identified *vs* untreated control) were assessed for allergenicity using AllergGAtlas database and 10 allergenic related proteins were identified, including vascular endothelial growth factor receptor 1 (FLT1), peroxiredoxin-1 (PRDX1), lysophosphatidic acid receptor 2 (LPAR2), 60 kDa heat shock protein (HSPD1), protein disulfide-isomerase A3 (PDIA3), L-lactate dehydrogenase (LDHA), interleukin-4 (IL4), phosphoglycerate kinase (PGK1), peroxisome proliferator-activated receptor delta (PPARD), and beta-actin (ACTB) (**Table 5**). Among the identified 10 allergenic-related proteins, LPAR2, HSPD1, PGK1, PPARD, and ACTB were found to be upregulated, but FLT1, PRDX1, PDIA3, LDHA, and IL4 were found to be downregulated when compared to untreated control (**Table 5**). Furthermore, allergenicity assessment of the 73 proteins (identified *vs* 3F4 treated control) revealed 5 allergenic-related proteins, including LDHA, LPAR2, elongation factor 1-alpha (EEF1A1), FLT1, and gamma actin-like protein (ACTG1) (**Table 6**). Among the 5 allergenic proteins, LDHA, EEF1A1 and FLT1 were found to be downregulated and LPAR2 as well as ACTG1 were found to be upregulated (**Table 6**).

**Table 5:** Properties of the identified allergenic proteins following direct antibody treatment (DAT) and direct microglia treatment (DMT) on the mouse primary neuron (MPN) cells in comparison with untreated cells.

Accession	Gene ID	Description	Peptides	Unique peptides	Confidence score	Anova (p)	Max fold change	Highest Mean	Lowest Mean
**Direct Antibody Treatment (DAT)**									
**A0A0R4J0A4**	Flt1	Vascular endothelial growth factor receptor 1	4	4	15.6	7.59E-05	5.69	NOT	DAT
**B1AXW5**	Prdx1	Peroxiredoxin-1 (Fragment)	8	6	60.2	9.35E-05	2.44	NOT	DAT
**A0A571BEW5**	Lpar2	Lysophosphatidic acid receptor 2	3	2	19.3	0.000114	10.6	DAT	NOT
**P63038**	Hspd1	60 kDa heat shock protein_ mitochondrial	48	43	479	0.008456	1.76	DAT	NOT
**F6Q404**	Pdia3	Protein disulfide-isomerase A3 (Fragment)	6	1	34.6	0.00903	1.79	NOT	DAT
**Q3TCI7**	Ldha	L-lactate dehydrogenase	15	4	126	0.011567	3.83	NOT	DAT
**Q91Y50**	Il4	Interleukin-4	3	1	27	0.015679	2.02	NOT	DAT
**S4R2M7**	Pgk1	Phosphoglycerate kinase	12	1	115	0.018003	1.54	DAT	NOT
**A0A3B2W7W2**	Ppard	Peroxisome proliferator-activated receptor delta	19	17	121	0.036966	1.43	DAT	NOT
**Q3UBP6**	Actb	Uncharacterized protein	36	4	356	0.040245	1.39	DAT	NOT
**Direct Microglia Treatment (DMT)**									
**A0A0R4J0A4**	Flt1	Vascular endothelial growth factor receptor 1	4	4	15.6	8.51E-05	10.3	NOT	DMT
**Q3TD08**	Ndrg1	Uncharacterized protein	6	6	32	0.001982	2.86	DMT	NOT
**Q78NA6**	Rag1	V(D)J recombination-activating protein 1	5	3	22.3	0.002166	2.98	NOT	DMT
**Q61916**	Muc5ac	Mucin (Fragment)	3	1	24.4	0.013162	1.5	DMT	NOT
**A0A571BEW5**	Lpar2	Lysophosphatidic acid receptor 2	3	2	19.3	0.013879	2.94	DMT	NOT
**Q3UBS0**	Apoe	Uncharacterized protein	7	5	38.8	0.015268	2.15	NOT	DMT
**Q99LX0**	Park7	Protein/nucleic acid deglycase DJ-1	7	7	58.3	0.044158	1.36	NOT	DMT

The properties were identified by Progenesis Software after the LC-MS analysis.

**Table 6:** Properties of the identified allergenic proteins following direct antibody treatment (DAT) and direct microglia treatment (DMT) on the mouse primary neuron (MPN) cells in comparison with 3F4 antibody treated cells.

Accession	Gene ID	Description	Peptides	Unique peptides	Confidence score	Anova (p)	Max fold change	Highest Mean	Lowest Mean
**Direct Antibody Treatment (DAT)**									
**Q3TCI7**	Ldha	L-lactate dehydrogenase	15	4	126	1.31E-05	14.9	3F4	DAT
**A0A571BEW5**	Lpar2	Lysophosphatidic acid receptor 2	3	2	19.3	1.65E-05	32.4	DAT	3F4
**Q58E64**	Eef1a1	Elongation factor 1-alpha	22	2	253	0.000541	9.08	3F4	DAT
**A0A0R4J0A4**	Flt1	Vascular endothelial growth factor receptor 1	4	4	15.6	0.000542	5.12	3F4	DAT
**Q9QZ83**	Actg1	Gamma actin-like protein	25	1	267	0.020739	2.44	DAT	3F4
**Direct Microglia Treatment (DMT)**									
**Q78NA6**	Rag1	V(D)J recombination-activating protein 1	5	3	22.3	0.000645	4.79	3F4	DMT
**Q546G4**	Alb	Serum albumin	29	26	194	0.023819	2.29	DMT	3F4
**P16045**	Lgals1	Galectin-1	3	3	20	0.027397	2.06	DMT	3F4
**Q3UBS0**	Apoe	Uncharacterized protein	7	5	38.8	0.03547	2.23	3F4	DMT

The properties were identified by Progenesis Software after the LC-MS analysis.

Analysis of individual direct anti-PrP antibody treatment effect on MPN (*vs* no treatment) showed that FLT1, PRDX1, LPAR2, PDIA3 and IL4 are present after ICSM18 and ICSM35 treatment, but PGK1 and HSPD1 were found to be present after ICSM35 treatment when compared with untreated cells (**Table 7**). However, analysis of individual direct anti-PrP antibody treatment effect on MPN (*vs* 3F4) revealed that LDHA, LPAR2, EEF1A1, FLT1 and ACTG1 are present after ICSM18 and ICSM35 treatment (**Table 8**).

**Table 7:** Identification of antibody-specific allergenic proteins following direct antibody treatment (DAT) and direct microglia treatment (DMT) on mouse primary neuron (MPN) cells in comparison with untreated cells.

Comparison with No Treatment (NOT)						
Treatment	Accession ID	Gene ID	ICSM18	CTL (NOT)	ICSM35	CTL (NOT)
**Direct Antibody Treatment** **(DAT)**	A0A0R4J0A4	Flt1	-	√	-	√
	B1AXW5	Prdx1	-	√	-	√
	A0A571BEW5	Lpar2	√	-	√	-
	P63038	Hspd1	-	-	√	-
	F6Q404	Pdia3	-	√	-	√
	Q3TCI7	Ldha	-	-	-	-
	Q91Y50	Il4	-	√	-	√
	S4R2M7	Pgk1	-	-	√	-
	A0A3B2W7W2	Ppard	-	-	-	-
	Q3UBP6	Actb	-	-	-	-
**Direct Microglia Treatment** **(DMT)**	A0A0R4J0A4	Flt1	-	√	-	√
	Q3TD08	Ndrg1	√		√	
	Q78NA6	Rag1	-	√	-	√
	Q61916	Muc5ac	-	-	√	-
	A0A571BEW5	Lpar2	-	-	√	-
	Q3UBS0	Apoe	-	√	-	√
	Q99LX0	Park7	-	-	-	-

(√) Upregulated and (-) Downregulated.

**Table 8:** Identification of antibody-specific allergenic proteins following direct antibody treatment (DAT) and direct microglia treatment (DMT) on mouse primary neuron (MPN) cells in comparison with 3F4 antibody treated cells.

Comparison with 3F4						
Treatment	Accession ID	Gene ID	ICSM18	CTL (3F4)	ICSM35	CTL (3F4)
**Direct Antibody Treatment** **(DAT)**	Q3TCI7	Ldha	-	√	-	√
	A0A571BEW5	Lpar2	√	-	√	-
	Q58E64	Eef1a1	-	√	-	√
	A0A0R4J0A4	Flt1	-	√	-	√
	Q9QZ83	Actg1	√	-	√	-
**Direct Microglia Treatment** **(DMT)**	Q78NA6	Rag1	-	√	-	√
	Q546G4	Alb	-	-	√	-
	P16045	Lgals1	-	-	-	-
	Q3UBS0	Apoe	-	--	-	√

(√) Upregulated and (-) Downregulated.

Protein-protein interaction analysis of the identified 10 allergenic-related proteins revealed that FLT1, PRDX1, PDIA3, IL4, PGK1, HSPD1, LDHA and ACTB are part of the same interactome, however, PDIA3 and HSPD1 were observed to interact directly with PRNP (**Figure 3B**). Protein-protein interaction analysis of the 5 allergenic-related proteins (*vs* 3F4) revealed that EEF1A1 and ACTG1 directly interact with each other, whereas LDHA and FLT1 interact *via* other proteins (**Figure S2**).

### Co-Culture of Anti-PrP Antibody Treated-Microglia with Neurons Leads to Neuronal Type 2-Like Hypersensitivity *In Vitro*

**Figure 4:**
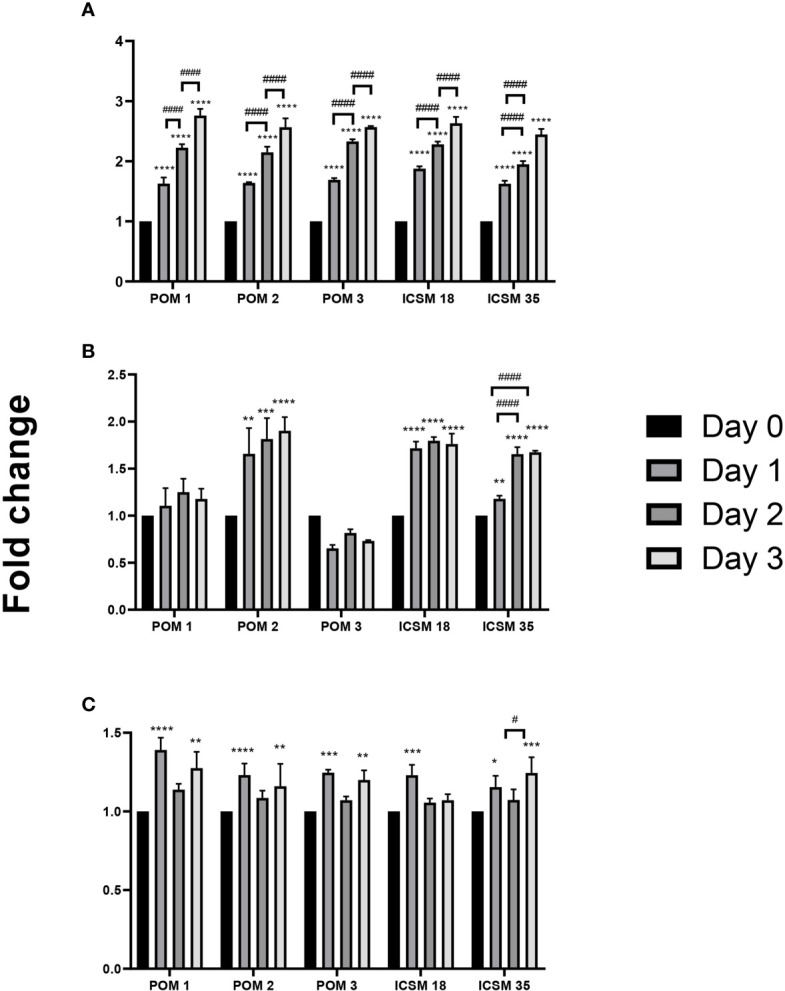
Activation of microglia cells (N11) following treatment with anti-PrP antibodies. Measurement of proinflammatory cytokines following direct anti-PrP antibody treatment of mouse microglia (N11) cell line in cell culture supernatant. Herein, fold change of NO **(A)**, IL-6 **(B)** and TNF-α **(C)** production on day 1, 2 and 3 post-antibody treatments compared to untreated cells (day 0). *p < 0.05, **p < 0.01, ***p < 0.001, ****p < 0.0001 *vs.* day 0, ^#^ *p* < 0.05, ^####^ *p* < 0.0001 compared between day 1, 2 and 3. P values were obtained by One-way ANNOVA followed by Tukey’s multiple comparisons test analysis by GraphPad Prism 8.

Initially, we measured levels of NO, IL6 and TNF-α in the supernatants of N11 following treatment with anti-PrP antibodies to confirm the activation status of the cells (**Figure 4**). Here, we show that treatment with POM1 and POM2 as well as ICSM18 and ICSM35 led to substantial accumulation of NO and IL6 in the cell culture media supernatant at day 1, 2 and 3, with exception of POM3 which led to increased synthesis of NO but not IL6. Moreover, TNF-α release into the supernatant was found to be marginally increased on day 1 and 3 post antibody treatment with POM1, POM2, POM3, ICSM18 and ICSM35.

Co-culture of N2a cells with anti-PrP antibody-treated N11 cells led to the identification of 2346 proteins (only *p* < 0.05) after LC-MS analysis when compared with co-culture of N2a cells with untreated-N11 cells. Out of the 2346 proteins, only the differentially expressed proteins were considered using maximum fold change ≥10, at least 2 identified unique peptides and a confidence score ≥ 40. The stringent parameters used here led to the identification of 113 proteins (**Table S4**). The 113 proteins were assessed for allergenicity, and 8 proteins were confirmed to be allergenic (**Table 9**), including IF rod domain-containing protein (VIM), peroxiredoxin-1 (PRDX1), Legumain (LGMN), cytoskeletal beta-actin (ACTB), V(D)J recombination-activating protein 1 (RAG1), L-lactate dehydrogenase (LDHA), Receptor-type tyrosine-protein phosphatase C (PTPRC), and TIR domain-containing protein (TLR3). Among the identified 8 allergenic-related proteins, 7 (VIM, LGMN, ACTB, RAG1, LDHA, TLR3, PTPRC) showed the highest mean for DMT when compared with N2a cultured with untreated-N11 cells (**Table 9**).

**Table 9:** Properties of the identified allergenic proteins following direct microglia treatment (DMT) on the neuroblastoma cell line.

Accession	Gene ID	Protein Name	Anova (p)	Max Fold Change	Confidence Score	Peptides	Unique Peptides	Highest Mean	Lowest Mean
**Q3TWV0**	Vim	IF rod domain-containing protein	0.000478	221	865	61	2	DMT	Control
**B1AXW5**	Prdx1	Peroxiredoxin-1 (Fragment)	0.010465	10.7	280	28	3	DMT	DMT
**A2RTI3**	Lgmn	Legumain	3.27E-07	443	51.3	8	2	DMT	DMT
**O89054**	Actb	Cytoskeletal beta-actin (Fragment)	2.63E-05	28.2	201	16	2	DMT	DMT
**Q78NA6**	Rag1	V(D)J recombination-activating protein 1	2.91E-13	13.5	95.2	16	5	DMT	DMT
**Q3UDU4**	Ldha	L-lactate dehydrogenase	7.9095E-05	13.5	474	41	6	DMT	DMT
**Q3TM31**	Tlr3	TIR domain-containing protein	0.043612	14.8	43.2	9	2	DMT	DMT
**P06800**	Ptprc	Receptor-type tyrosine-protein phosphatase C	6.11E-07	12.5	41.1	6	2	DMT	DMT

The properties were identified by Progenesis Software after the LC-MS analysis.

**Figure 5:**
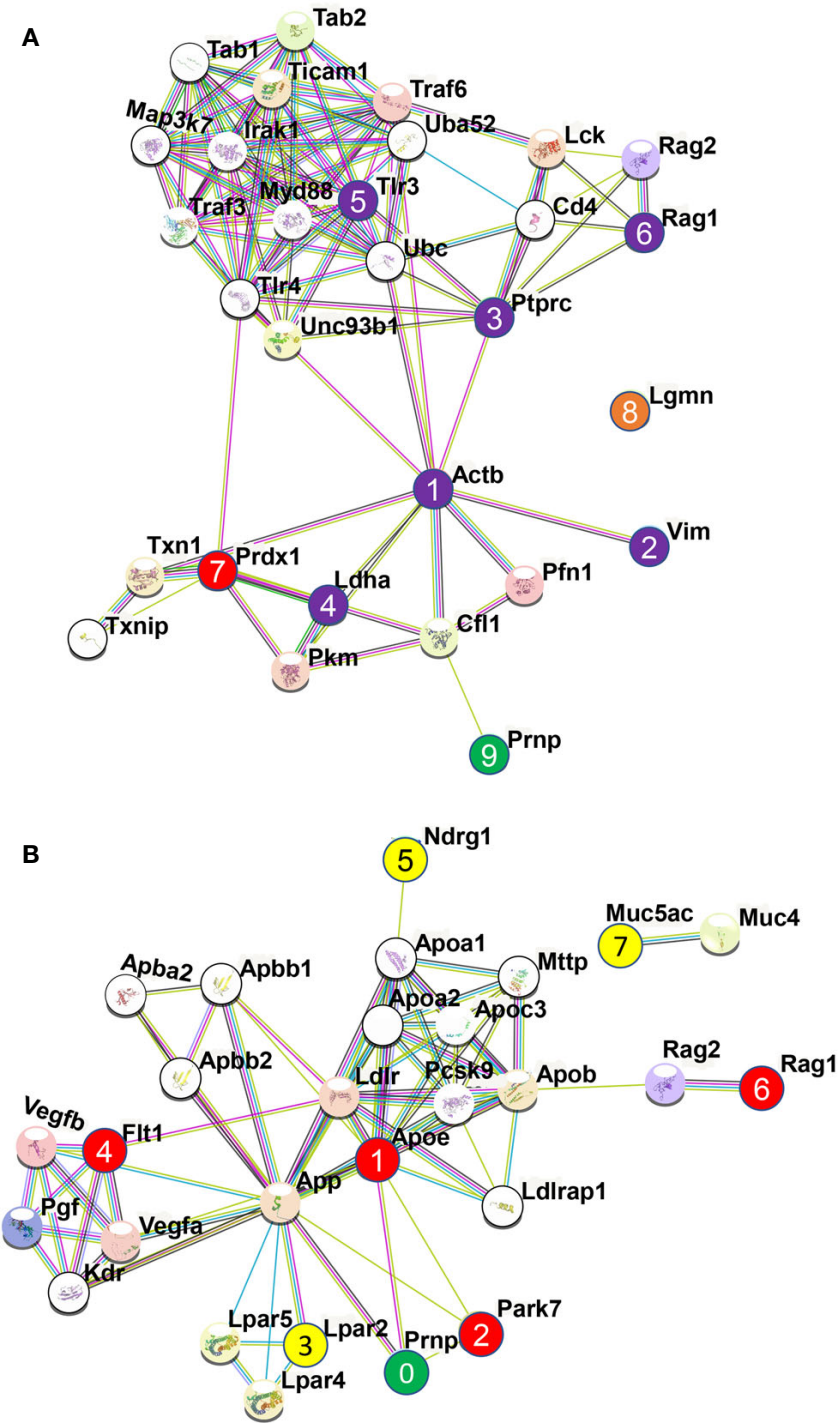
Protein-protein interaction analysis among the identified allergenic genes following direct microglial treatment (DMT) with anti-PrP antibodies in cell line and mouse primary neuron. The analysis was performed using STRING v11.0 server. The *Mus Musculus* database was used as the host organism for characterising the protein interactions. **(A)** Protein-protein interaction among the identified 8 allergenic genes following direct microglial treatment (DMT) with anti-PrP antibodies in mouse neuroblastoma cell line. Herein, interaction among the allergenic proteins and with PrP^C^ where the numbered purple circles (numbered 1, 2, 3, 4, 5, 6) and numbered red circle refer to allergenic-related proteins identified following DMT and shown here to be part of the same interactome. The red circle (numbered 7) was shown to be downregulated and the orange circle (numbered 8) is not part of the interactome with other allergenic-related proteins. Finally, the green circle (numbered 9) represents the prion protein, PrP^C^. **(B)** Protein-protein interaction among the 7 identified allergenic genes following direct microglial treatment (DMT) with anti-PrP antibodies in mouse primary neuron (MPN). Herein, Interaction among the allergenic proteins (numbered 1, 2, 3, 4, 5, 6 & 7) and with PrP^C^ (numbered 0) following direct microglia treatment where red and yellow color indicate the downregulated and upregulated genes, respectively and green color represents the cellular prion protein PrP^C^.

Protein-protein interaction of the identified 8 allergenic-related proteins showed that PrP interacts with ACTB *via* Cofilin-1 (CFL1), while VIM, PTPRC, and LDHA directly interacts with ACTB (node 1, 2, 3, 4 in **Figure 5A**). It was previously shown that overexpression of PrP^C^ itself activates the NADPH oxidase (NOS) for reactive oxygen species (ROS) production that initiates the cofilin activation and finally induce cofilin-actin rods in hippocampal neurons (53). Protein-protein interaction revealed that both RAG1 (node 6 in **Figure 5A**) and TLR3 (node 5 in **Figure 5A**) indirectly interact with ACTB (node 1 in **Figure 5A**) *via* PTPRC (node 3 in **Figure 5A**) while PRDX1 (node 7 in **Figure 5A**) indirectly interacts with ACTB *via* LDHA (node 4 in **Figure 5A**). A study by Wagner and co-workers showed that PRDX6 was upregulated in scrapie-infected mice and neuronal cell lines (54). However, LGMN (node 8 in **Figure 5A**) did not interact with any of the identified allergenic-related proteins as well as with PrP^C^ protein (node 9 in **Figure 5A**). Among the identified allergenic-related proteins, VIM was found to be involved in the progression of allergic diseases *via* inflammasome (55, 56) and VIM-P38MAPK complex facilitates mast cell activation *via* FcεRI/CCR1 activation (57). LDHA was identified as a potential marker in allergic alveolitis, airway inflammation, allergic encephalomyelitis, asthma disease (58–61). PRDX1 was found as a negative regulator of inflammation (62), Th2-type airway inflammation, and allergen-related hyperresponsiveness (63). PTPRC was found to be associated with asthma related phenotypes in a microarray analysis (64). LGMN was found to be involved in allergic reaction by potentiating antigen processing (65). A study by Sehra et al. showed that RAG1-deficient mice exhibited reduced mast cell infiltration when it was used as a chronic model of allergic inflammation (66). TLR3 activation in an established experimental allergic asthma mice model increased the release of proinflammatory cytokines and mucus production which was also associated with the increased production of interleukin 17 (IL-17A) by natural killer (NK) cells (67).

The highest versus lowest mean of the allergenic-related protein expression by individual antibody treatments when compared to untreated control is shown in **Table 10**. Here, we show that ICSM18, ICSM35, and SAF70 share 62.5% allergy related proteins (5 proteins: VIM, LGMN, RAG1, LDHA, and PTPRC). POM2 and SAF32 showed 75% effect with 6 proteins, but the proteins were found to be different for both POM2 (VIM, ACTB, LGMN, RAG1, LDHA, and TLR3) and SAF32 (VIM, ACTB, LGMN, RAG1, LDHA, and PTPRC). However, the lowest effect of antibody was observed for both POM1 (4 proteins; VIM, ACTB, RAG1, and TLR3) and POM3 (4 proteins; VIM, ACTB, LGMN, and LDHA) with 50% effect.

**Table 10:** Identification of antibody-specific allergenic proteins following direct microglia treatment (DMT).

Accession ID	Gene ID	ICSM Antibodies				POM Antibodies						SAF Antibodies			
		ICSM18	CTL	ICSM35	CTL	POM1	CTL	POM2	CTL	POM3	CTL	SAF32	CTL	SAF70	CTL
**Q3TWV0**	Vim	√	-	√	-	√	-	√	-	√	-	√	-	√	-
**B1AXW5**	Prdx1	-	√	-	√	-	-	-	√	-	-	-	√	-	√
**A2RTI3**	Lgmn	√	-	√	-	-	-	√	-	√	-	√	-	√	-
**O89054**	Actb	-	-	-	-	√	-	√	-	√	-	√	-	-	-
**Q78NA6**	Rag1	√	-	√	-	√	-	√	-	-	-	√	-	√	-
**Q3UDU4**	Ldha	√	-	√	-	-	-	√	-	√	-	√	-	√	-
**Q3TM31**	Tlr3	-	-	-	-	√	-	√	-	-	-	-	-	-	-
**P06800**	Ptprc	√	-	√	-	-	-	-	-	-	-	√	-	√	-

(√) Upregulated and (-) Downregulated.

**Figure 6:**
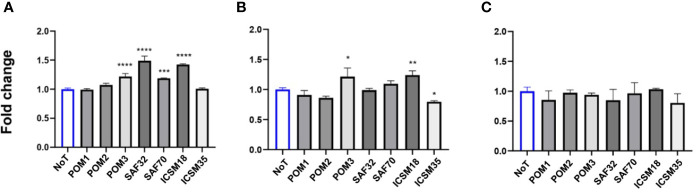
Production of proinflammatory cytokines following direct microglial treatment (DMT) with anti-PrP antibody treatment of mouse neuroblastoma cell line. Herein, fold change of NO **(A)**, IL-6 **(B)** and TNF-α **(C)** production in N2a following DMT. *p < 0.05, **p < 0.01, ***p < 0.001, ****p < 0.0001 *vs.* no treatment (NoT). P values were obtained by One-way ANNOVA followed by Tukey’s multiple comparisons test analysis by GraphPad Prism 8.

Co-culture of N2a cells with anti-PrP antibody-treated N11 cells led to substantial accumulation of NO and IL6 but not TNF-α in the cell culture media supernatant at day 3 (**Figure 6**). More specifically, POM3, SAF32, SAF70 and ICSM18 led to a substantial increase of NO in the cell culture media supernatant; however, POM1, POM2 and ICSM35 did not show similar effect (**Figure 6A**). Moreover, POM3, ICSM18 and ICSM35 but not POM1, POM2 SAF32 and SAF70 also led to a substantial increase of IL6 in the cell culture media supernatants (**Figure 6B**). Interestingly, none of the antibodies had an effect on TNF-α release (**Figure 6C**). Of note, String analysis showed that IL6 interacts directly with 4 out of 8 allergenic-related proteins (ACTB, RAG1, TLR3, PTPRC) and indirectly with 2 out of 8 allergenic-related proteins *via* ACTB (VIM, LDHA) (data not shown).

Co-culture of untreated MPNs with ICSM18 or ICSM35-treated N11 led to the identification of 161 proteins (*p <*0.05) when compared with co-culture with MPN co-cultured with untreated N11. Furthermore, 77 proteins (*p <*0.05) were identified when compared with co-culture of MPN with 3F4-treated N11. Out of the 161 proteins and 77 proteins, only the differentially expressed proteins were considered using the *p* value < 0.05 and confidence score ≥15. After applying those parameters, we found 88 proteins (*vs* untreated) (**Table S5**) and 49 proteins (*vs* 3F4 treated) (**Table S6**). The allergenicity assessment of the 88 proteins (*vs* untreated) through identified 7 allergenic-related proteins, including FLT1, uncharacterized protein (NDRG1), RAG1, mucin (MUC5AC), LPAR2, uncharacterized protein (APOE), and protein/nucleic acid deglycase DJ-1 (PARK7) (**Table 5**). Moreover, Analysis of the 49 proteins (*vs* 3F4 treated) identified 4 allergenic-related proteins including, RAG1, serum albumin (ALB), galectin-1 (LGALS1) and APOE (**Table 6**). Protein-protein interaction analysis of the 7 allergenic-related proteins (*vs* untreated) revealed that *PRNP* directly interacts with APOE and PARK7 while FLT1, RAG1, LPAR2, NDRG1 are part of the same interactome (**Figure 5B**). Protein-protein interaction among the identified 4 allergenic-related proteins (*vs* 3F4) showed direct interaction between PRNP, APOE and ALB (**Figure S3**).

The individual effect of anti-PrP antibody treatment on MPN when compared with untreated control is shown in **Table 7**. Herein, NDRG1was upregulated while FLT1, RAG1 and APOE were downregulated after ICSM18 treatment (**Table 7**). Moreover, ICSM35 treatment upregulated NDRG1, MUC5AC and LPAR2 and downregulated FLT1, RAG1 and APOE (**Table 7**).

The individual effect of anti-PrP antibody treatment on MPN when compared with 3F4 treated control is shown in **Table 8**. No protein upregulation occurred after ICSM18 treatment, however downregulation of RAG1 was observed. ICSM 35 led to upregulation of ALB while both RAG1 and APOE were downregulated (**Table 8**).

In order to verify whether the allergenic-related proteins identified in MPN following DMT were specifically stimulated in neurons (and not in both neurons and microglia), we compared the proteome of the anti-PrP antibody-treated microglia without co-culture with neurons and found that anti-PrP antibody-treated microglia only did not display any common allergy-related proteins with DMT (**Table S7**) indicating that our identified allergy-related proteins were specifically activated in neurons.

**Figure 7:**
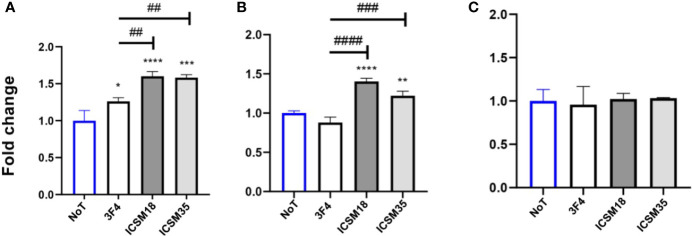
Production of proinflammatory cytokines following direct microglial treatment (DMT) with anti-PrP antibody treatment of mouse primary neuron (MPN). Herein, fold change of NO **(A)**, IL-6 **(B)** and TNF-α **(C)** production in MPN following DMT. *p < 0.05, **p < 0.01, ***p < 0.001, ****p < 0.0001 *vs.* no treatment (NoT). ^##^ *p* < 0.01, ^###^ *p <* 0.001, ^####^ *p* < 0.0001 *vs.* 3F4 antibody treatment. *P* values were obtained by One-way ANNOVA followed by Tukey’s multiple comparisons test analysis by GraphPad Prism 8.

Co-culture of MPN with ICSM18 or ICSM35 anti-PrP antibody-treated N11 cells led to substantial accumulation of NO and IL6 (**Figures 7A, B**) but not TNF-α (**Figure 7C**) in the cell culture media supernatant at day 3 when compared with both untreated and 3F4-treated cells. String analysis showed that IL6 interacts directly with 5 out of 7 allergenic-related proteins *vs* untreated (APOE, RAG1, MUC5AC, FLT1, PARK7) and directly with 4 out of 4 allergenic-related proteins *vs* 3F4-treated control (RAG1, ALB, LGALS1, APOE) (data not shown).

### Contactless Co-Culture of Anti-PrP Antibody Treated-Microglia with Neurons Leads to Neuronal Type 2-Like Hypersensitivity *In Vitro*

Contactless co-culture of anti-PrP antibody treated microglia N11 and N2a cells was designed to verify whether the allergenic effects caused by DMT were due to a direct cognate interaction of N2a and N11 or *via* indirect release of microglial factors which in turn might have led to hypersensitivity. N11 cells were initially treated with anti-PrP antibodies, including ICSM18, ICSM35, POM1, POM2, POM3, SAF32 or SAF70 on cell culture inserts before placing the inserts containing antibody-treated microglia on cell culture plate containing untreated N2a cells (IMT). IMT resulted in an initial dataset of 292 proteins (*p* < 0.05) after LC-MS analysis. Differentially expressed proteins (*p* < 0.05) were considered with a maximum fold change ≥10 and at least 2 identified unique peptides and a confidence score ≥ 10 and identified a total of 11 proteins (**Table S8**). Out of the 11 proteins, AllergGAtlas database only identified Integrin beta-4 (ITGB4) (upregulated, *p*=0.034, maximum fold change 45, confidence score 33.6, peptide 6, unique peptide 3) as being allergenic. The protein-protein interaction analysis showed that ITGB4 indirectly interacts with PRNP *via* ITGB6 and NCAM 1 (**Figure S4**).

### Anti-PrP Antibody Treatment of Neurons Leads to Differential Expression of FcϵR1a

**Figure 8:**
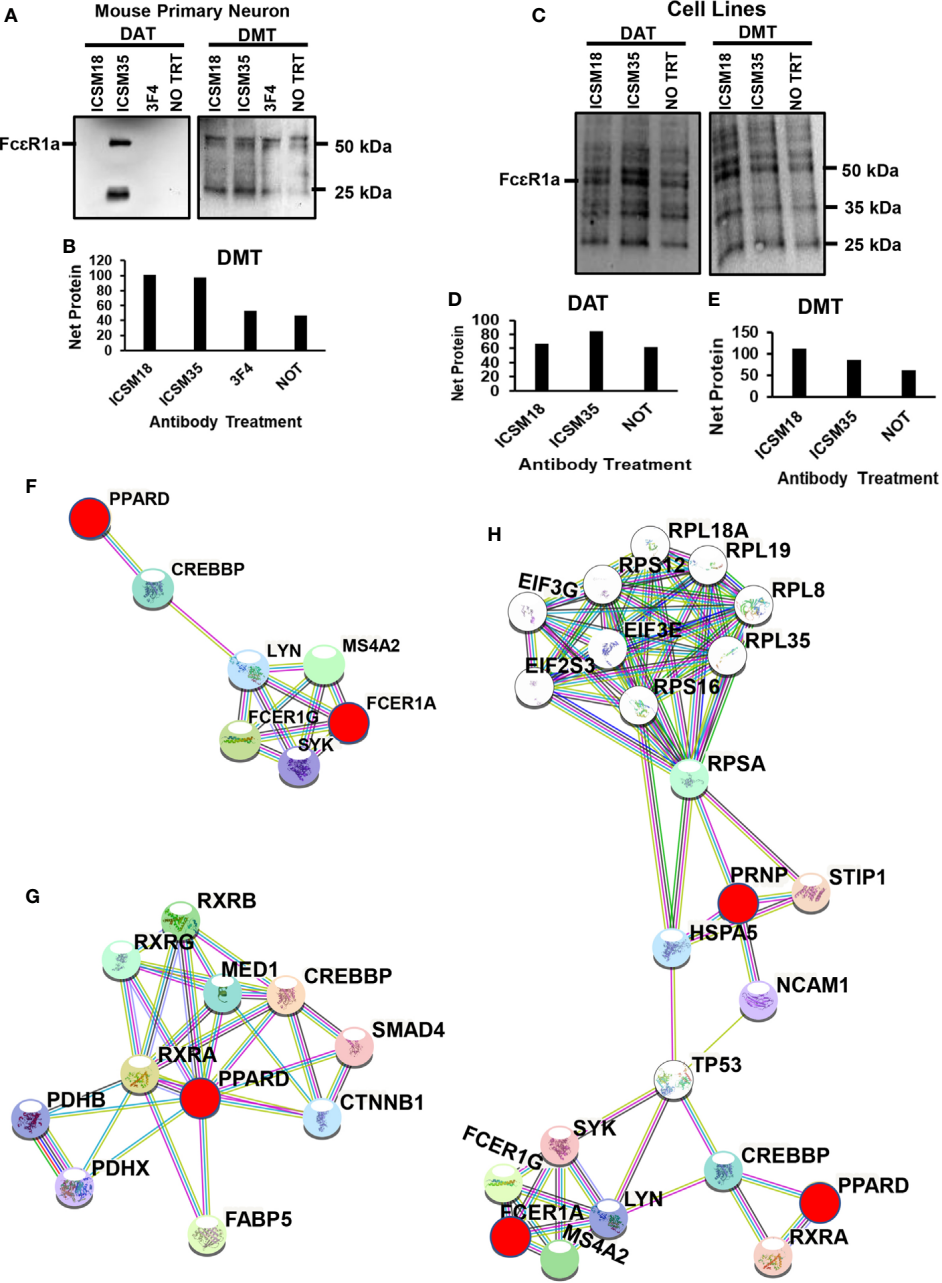
Differential expression of FcεR receptors following anti-PrP antibody treatment to mouse primary neuron cells and mouse neuroblastoma cell line. Western blotting of **(A)** direct antibody treatment (DAT) and direct microglia treatment (DMT) of mouse primary neuron cells **(B)** densitometry analysis of **(A)**. **(C)** DAT and DMT of mouse neuroblastoma cells **(D, E)** densitometry analysis of **(F)**. **(F)** protein-protein interaction of PPARD with FcεR1a **(G)** Illustration of protein interactome with PPARD **(H)** Illustration of protein-protein interactions between PPARD, FcεR1a and PrP^C^.

The high affinity IgE receptor (FcϵRI) is a tetrameric receptor complex which is composed of one α-subunit (FcϵRIα), one β-subunit (FcϵRIβ) (68) and two γ-subunits (FcϵRIγ) (69–71). During a classic IgE-mediated allergic immune response, FcϵRIα directly binds IgE with high affinity, while FcϵRIβ and FcϵRIγ are responsible for mediating intracellular signals (68, 71–73). Furthermore, binding of the IgG receptors FcγRII and FcγRIII receptors to the IgE-immune complexes contribute to the induction of cell activation (74). Ujike and colleagues also showed that FcγRII and FcγRIII not only modulates IgG-mediated hypersensitivity responses but also acts as an effective regulators of IgE-mediated reactions (75). The IgG/FcγR pathway was shown to induce an anaphylactic reaction following activation of basophils, macrophages, and neutrophils (76–78). The IgG FcγR and IgE FcϵRI share a common γ subunit and comparable signaling pathways. Falanga and colleagues showed that the Lyn and Fyn kinases were activated after FcγR stimulation (79). Of importance, the authors demonstrated that both Fyn and Lyn regulate FcγR-mediated degranulation and cytokine and chemokine and histamine release was regulated by Fyn and Lyn. Moreover, clustering of PrP^C^ was shown to activate Fyn kinase as both localize lipid rafts (14). Finally, PrP^C^-dependent signal transduction revealed that PrP^C^ coupled with Fyn kinase following anti-PrP antibody-mediated cross-linking (15). Herein, we wanted to verify whether cross-linking neuronal/microglial PrP^C^ with anti-PrP antibody alters the expression of FcϵRI. Western blot analysis displayed a ~50 kDa and ~25 kDa band corresponding to FcϵRIα and FcϵRIγ subunits respectively, in MPN after DAT with ICSM35 (**Figure 8A**). DAT of MPN with ICSM18, 3F4 and untreated cells did not display any band (**Figure 8A**). However, Western blotting and densitometry analysis following DMT with ICSM18 and ICSM35 of MPN displayed substantially higher ~50 kDa and ~25 kDa band intensity corresponding to FcϵRIα and FcϵRIγ respectively when compared with DMT with 3F4 and untreated cells (**Figures 8A, B**). Similarly, Western blotting and densitometry analysis following DMT with ICSM18 and ICSM35 of co-cultured N2a with ICSM18 or ICSM35-treated N11 displayed higher ~50 kDa, ~35 kDa and ~25 kDa band intensity corresponding to FcϵRIα, FcϵRIβ and FcϵRIγ respectively when compared with untreated cells (**Figures 8C, E**). In contrast, DAT with ICSM35 of N2a displayed higher ~50 kDa, ~35 kDa and ~25 kDa band intensity corresponding to FcϵRIα, FcϵRIβ and FcϵRIγ respectively when compared with ICSM18 treated and untreated cells (**Figures 8C, D**).

**Figure 9:**
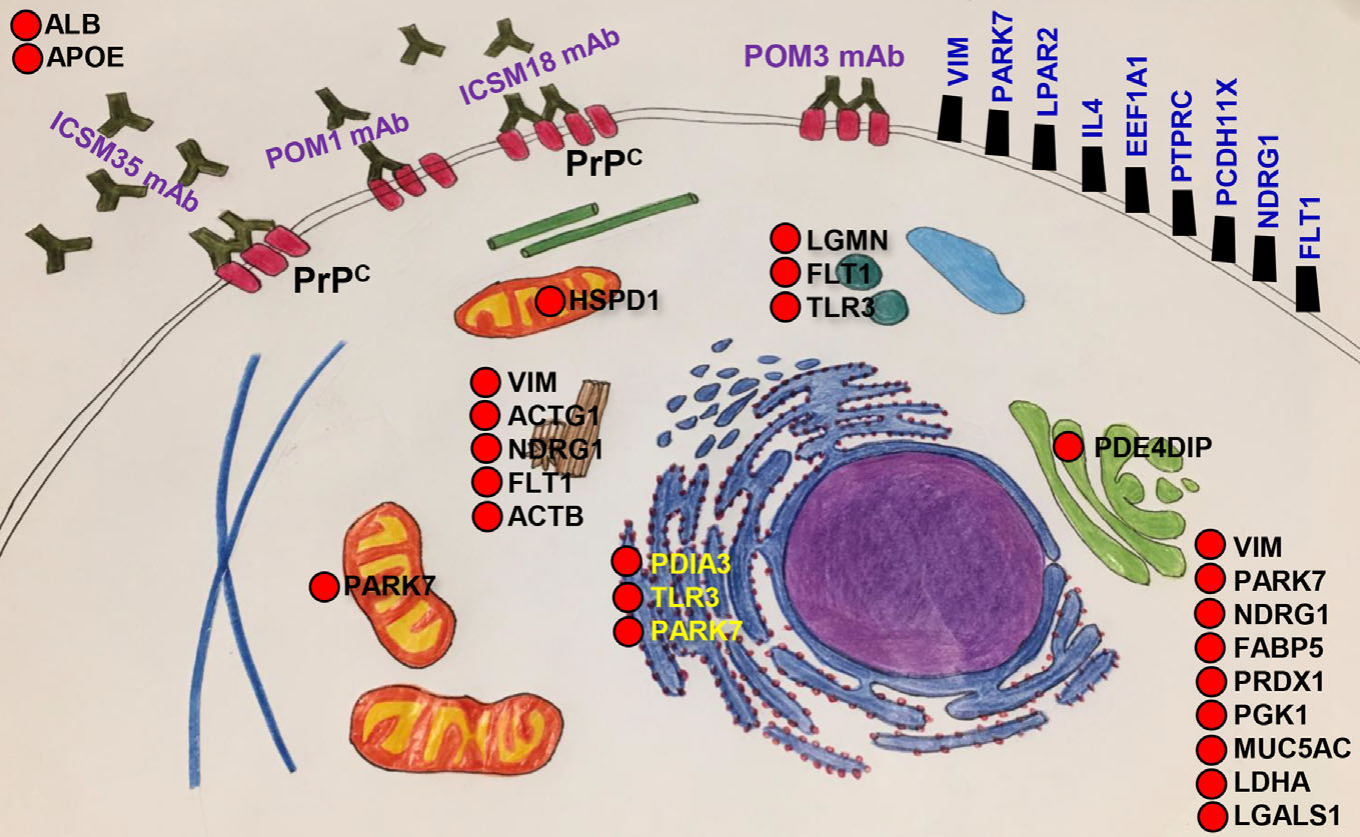
Cellular and subcellular localisation of allergenic-related proteins. Allergenic related proteins activated following direct anti-PrP antibody and microglia treatments of neuroblastoma cell line and mouse primary neurons are grouped according to cellular location.

In order to identify the protein interactors with FcϵRIα following DAT with ICSM35 of MPN, the corresponding gel bands were trypsin digested prior to LC-MS analysis. Here, 12 proteins were identified, of which peroxisome proliferator-activated receptor delta (PPARD) was recognized as an allergenic-related protein (**Table S9**). Protein-protein interaction of PPARD and FcϵRIα showed that they are located in the same protein network and interacting through two signaling molecules, namely CREB-binding protein (CREBBP) and Tyrosine-protein kinase Lyn (LYN) (**Figure 8F**). Further protein network analysis showed that PPARD directly interacts with several allergy and inflammation proteins such as fatty acid-binding protein (FABP5) (80, 81), retinoic acid receptor RXR-alpha (RXRA) (82), pyruvate dehydrogenase E1 component (PDHX) (83), catenin beta-1 (CTNNB1) (84–86), mothers against decapentaplegic homolog 4 (SMAD4) (87, 88) and retinoic acid receptor RXR-beta (RXRB) (89) (**Figure 8G**) confirming its association with allergy. This interaction analysis also revealed that PRNP interacts with PPARD and FcεR1a *via* a network of other proteins as part of the same interactome (**Figure 8H**). As summarized in **Figure 9**, most of the proteins associated with all treatments with anti-PrP antibodies have their primary location in the plasma membrane and cytoplasm, whereas only 3 were seen in the nucleus, 2 in the endoplasmic reticulum, 1 in the Golgi, 1 in the mitochondria, and 1 in the lysosome. NDRG1, PARK7 and VIM proteins have a ubiquitous cellular location. Proteins involved in negative regulation of reactive oxygen species, of neuronal apoptosis and development of neuronal projections were highly represented.

## Discussion

Hypersensitivity reactions are triggered by the immune response. Little is known about the so-called IgG-mediated neuronal hypersensitivity, however, a body of new emerging studies suggest that hypersensitivity is an important feature in response to IgG immunotherapy or disease-associated auto-antibodies (90–92). Fcγ receptors (FcγRs) are known to mediate protective immune functions *via* binding of IgG molecules to the Fc domain in addition to modulating the adaptive immune response. FcγRs have been implicated in hypersensitivity reactions; for instance, Fcγ-chain-deficient mice were protected against a number of autoimmune disorders [reviewed in (93)], suggesting an important role for FcγRI in hypersensitivity reactions. Furthermore, functional polymorphism in FCγRs genes was shown to play an important role in the pathogenesis of allergy (94). A number of *in vitro* and *in vivo* studies have demonstrated the presence of FcγRs in neurons. Previous reports implicated IgG in inducing a neuronal hypersensitivity reaction (91). Of importance, Fuller et al. highlighted the importance of increased expression and ligation of FcγRs in the CNS as a result of administration of therapeutic antibodies or by endogenous IgG which resulted in vascular damage and exacerbation of neurodegeneration (95). Furthermore, experimental treatment with anti-PrP antibodies directed against PrP^C^ led to neuronal apoptosis based on microscopic assessments (23, 24, 26). Upon further analysis, some of these studies also revealed that anti-PrP antibodies induced activation of allergenic-related proteins identified by the AllerGAtlas database (21, 27, 28). We therefore sought molecular confirmation of a neuronal type 2-like hypersensitivity process associated with anti-PrP antibody treatment of neurons and microglia *in vitro*. Initially, we performed *in silico* analysis to predict the most antigenic epitopes from the huPrP 3D structure and to verify whether some of the predicted motifs overlap with those recognized by the reported ‘neurotoxic’ anti-PrP antibodies such as ICSM and POM antibodies (40, 43). The *in-silico* analysis revealed a set of antigenic B cell linear epitopes located on the flexible tail (FT) region. Interestingly, epitopes L2 and L9/L10 were mapped to the ‘neurotoxic’ antibodies ICSM35 (26, 40) and POM3 (23, 24, 27, 43). We also confirmed that L2, L9 and L10 were toxic following assessment with the ToxinPred server (50) by quantitative matrix based method (QM method). The *in-silico* analysis also revealed a set of antigenic B cell linear epitopes located on the globular (GD) region of which L4 was mapped to the ‘neurotoxic’ antibodies ICSM18 (24, 26, 40) and POM1 (24, 27, 43). The ToxinPred server also identified L4 as being toxic. While performing *in-silico* analysis to assess antigenicity and toxicity of epitopes located on the huPrP 3D structure, we also noticed that some antigenic epitopes were predicted to be allergenic by AllergenFP (51) and AllerTop (52) allergenicity prediction servers. A total of 7 B cell linear epitopes were identified as allergenic. Of interest, L4 was mapped to the neurotoxic antibodies POM1 and ICSM18 and L2 was mapped to ICSM35 and POM3 while L9 and L10 were both mapped to POM2. POM1, a similar antibody to ICSM18 and mapped to the L4 epitope was previously shown to induce neurotoxicity *via* calpain (27). Our LC-MS data also shows that calpain 1 is activated by ICSM18 treatment, but on the contrary led to inhibition of calpain 3 (data not shown), probably *via* negative feedback following cross-linking PrP^C^. In addition to its newly characterized role in antibody-induced neuronal apoptosis, calpains have a well-established role in allergy (96–98). For instance, a study by Wu et al. showed that calpain 1 contributes to mast cell degranulation (96). Furthermore, inhibition of mGluRs, known to regulate histamine (34), abolished the anti-PrP antibody toxic effects (28). Taken together and in addition to the allergenic-related proteins associated with ICSM35 treatment reported by Tayebi and colleagues (21), this provides sufficient evidence to investigate the allergenic pathways potentially induced by treatment with anti-PrP antibodies which we refer to as “*IgG-Mediated Neuronal Hypersensitivity*”. To that end, we treated mouse primary neurons (MPN), mouse neuroblastoma (N2a) and microglia (N11) cell lines with anti-PrP antibodies then assessed the neuronal allergenic proteome following mass spectrometry analysis as well as expression of neuronal FcεR1a. ACTB was found to be activated in both MPN and N2a shown to interact with PrP^C^ *via* cofilin 1. Walsh and co-workers demonstrated that overexpression of PrP^C^ activates the NADPH oxidase (NOS) for reactive oxygen species (ROS) production which initiates cofilin activation and finally induce cofilin-actin rods in hippocampal neurons (53). Among the 10 allergenic-related protein identified following DAT of MPN, FLT1 was found to be directly interacting with IL4 in protein-protein interaction analysis. FLT1 was previously shown to be highly expressed in asthmatic patients (99), associated with allergic inflammation as an inducer that increases the allergic sensitization and also played a significant role in Th2 type inflammatory responses (100). IL4 identified following DAT of MPN directly interacting with both HSPD1 and ACTB. IL4 is a cytokine that plays a crucial role in the progression or pathogenesis of allergic diseases since it has a key role in the development of helper T-cell and the production of IgE (101–106). Shi and colleagues characterised the positive role of IL-4 in airway responsiveness in patients with allergic bronchial asthma (103). Further, Ryan and co-workers confirmed the inhibitory role of IL-4 on mouse bone marrow and fetal liver-derived mast cell FcεRI expression through a STAT6 transcription factor-dependent mechanism (107). A study by Niggemann et al. showed that IL-4 increases histamine release from mast cells and peripheral blood basophils in both *in vitro* and *in vivo* study where IL-4 plays priming effects on histamine release (108). In another study on IL-4 primed human cultured mast cells, enhanced IL-13 production was observed by cross-linking of FcεRI (109). Both IL-10 and IL-4 were detected in CSF of patients with Creutzfeldt-Jakob disease (CJD) (110).

HSPD1 has been found to be involved in the alveolar macrophages immune functions in relation to allergic asthma (111). Zanin-Zhorov et al. showed that HSPD1 can inhibit the Th1-mediated immune responses through the suppression of tumour necrosis factor α (TNF-α) (112). HSPD1 was found to be associated with PrP^C^ as a major interactor (113, 114).

PDIA3 identified following from DAT of MPN was found to be directly interacting with PRDX1 (found in both DAT of MPN and DMT of neuroblastoma cells), ACTB (found in DAT of MPN and both in DAT and DMT of neuroblastoma cells), and HSPD1. Recently, Krajewski and co-workers showed that protein disulfide isomerase catalytic activity plays an important role in the regulation of mast cell activation (115). They also showed that the inhibition of protein disulfide isomerase activity *via* pre-treatment of mast cells with curcumin or other protein disulfide isomerase inhibitor such as PACMA-31 are able to supress the IgE mediated activation and various cytokines secretion (115). Inouue et al. showed that PRDX1 protects against allergen-related hyperresponsiveness and Th2-type airway inflammation and involved in the inhibition of allergen-specific T-cell proliferation through immunological synapse (63). PPARD identified following DAT of MPN was found to be interacting with FABP5 (found in DAT of neuroblastoma cells). Expression of PRAR is altered during inflammatory responses such as airway inflammation, indicating that PPAR is involved in the pathogenesis of allergic asthma (116–120). PPARbeta/delta was found to be act modulators in many mediators of inflammation (118, 121–124) while the activation of PPARbeta/delta also play an important roles in inflammation by inducing expression of transforming growth factor-β1 (TGF-β1) (125) and sIL-1ra (126). Several studies have confirmed the inhibitory effect of PPAR agonists in immune and inflammatory responses, especially in the central nervous system inflammation and demyelination in experimental autoimmune encephalomyelitis (127–130). Kanakasabai and co-workers showed that PPARD agonist (GW501516 and L165041) ameliorates the experimental autoimmune encephalomyelitis in C57BL/6 mice by inhibiting T helper type 1 (Th1) and Th17 responses (131). The inhibitory effect of PPARD agonist was also found to be associated with an increase of cytokine IL-4 and IL-10 expression and decreased IL-12 and IL-23 expression in the CNS confirming the modulatory effect of PPARD agonist in experimental autoimmune encephalomyelitis (131).

Due to the important role played by microglia in the exacerbation of neuropathology in the prion and other related disorders, we sought to verify whether co-culture of N2a/MPN with anti-PrP antibody-treated N11 (DMT) leads to a neuronal hypersensitivity reaction.

A study by Toda et al. revealed that VIM-P38MAPK complex facilitates mast cell activation *via* FcϵRI/CCR1 activation (57). Amongst the DMT associated allergenic-related proteins, LDHA was found to interact with PRDX1. Interestingly, PRDX6, a protein that directly networks with our downregulated PRDX1, was shown to be upregulated in scrapie-infected mice and neuronal cell lines and controls expression of PrP^C^ and PrP^Sc^ in neuronal cells (54). PTPRC previously shown to be upregulated in brain of mice following infection of prion (132), is associated with asthma related phenotypes (64) and its ligation enhances the frequency of constitutive apoptosis in human eosinophils (133). RAG1 plays an important regulatory role in the reorganization and recombination of T cell receptor (TCR) and immunoglobulin (Ig) genes (134). A study by Sehra et al. showed that Rag1-deficient mice exhibited reduced mast cell infiltration when it was used as a chronic model of allergic inflammation (66). Of importance, RAG1 was a common protein identified in DMT with N2a and MPN (*vs* untreated and 3F4 treated) and was shown to directly interact with IL6 in String analysis. Further, we show that IL-6 was substantially increased in all DMTs, including co-cultures N11 with N2a (*vs* untreated) and N11 with MPN (*vs* untreated and *vs* 3F4 treated). Previous studies demonstrated the effect of treatment of toxic PrP peptide in inducing microglia activation and cytokine release (135). Garcao and colleagues showed that treatment of microglia led to increased IL-6 secretion, responsible for increased neuronal death (136, 137). Interestingly, none of the antibody treatment following DMT were able to affect the production and increase of TNF-α. Previous studies have demonstrated that IL6 antagonizes the action of TNF-α (138, 139).

Another allergenic protein identified following DMT is TLR3 that directly interact with PTPRC. TLR3 is a membrane protein which act as a pathogen recognition receptor and is expressed in the CNS and other cell types (140). TLR3 activation by poly (inosinic-cytidylic) acid in an established experimental allergic asthma mice model increased the release of proinflammatory cytokines and mucus production which was also associated with increased production of IL-17A by NK cells (67). Starkhammar and colleagues showed that combined stimulation of TLR3 and TLR4 causes airway hyperresponsiveness which is increased during an ongoing allergic inflammation (141). ALB was identified following DMT of MPN when compared with 3F4 treated cells. ALB is a multifunctional protein which is broadly distributed in the body and is used as a sensitizing mediator in various immunological pathways and also acts as a carrier of several drugs (142). The involvement of ALB in allergy has been described in several studies (143–146).

Analysis of individual anti-PrP antibody treatment identified that the highest allergenic effect, as assessed by the number of allergenic-related proteins, was associated with the GD and FT targeting antibodies ICSM18/SAF70 and ICSM35 which shared 5 common proteins. ICSM18 and 35 were produced in PrP-null mice against truncated hPrP^91-231^ while SAF70 was raised in hamsters using SAF preparation. However, both ICSM18 and SAF70 bind to a similar epitope on the GD but ICSM35 epitope is located on the FT domain. ICSM35 and SAF70 are of the same Ig isotype (IgG2b). It remains a challenge to pinpoint which of these antibody characteristics led to activation of the common proteins, however, there is strong indication that inherent antibody properties (e.g. epitope; isotype etc.) trigger similar allergenic pathways. POM2 and SAF32, two antibodies that bind to an epitope on the octa-repeat activated 6 proteins separately where 5 proteins were common and the protein TLR3 and PTPRC was found to be activated by POM2 and SAF32, respectively. SAF32, similar to SAF70 was raised in hamsters using SAF preparation and is an IgG2b, while POM2 was raised in PrP-null mice against full-length mPrP^23-231^ and is an IgG1. In this case, the common octa-repeat epitope appears to be playing a key role in triggering similar allergenic pathways by these 2 antibodies, however, all molecular aspects should also be considered, including antibody affinity for instance. It is noteworthy that among the 5 common proteins shared by POM2 and SAF32, 4 proteins were also common with ICSM18, ICSM35 and SAF70 treatments, possibly reflecting the involvement of several antibody properties. Finally, POM1 and POM3, raised in PrP-null mice against full-length mPrP^23-231^ and of IgG1 isotype with binding motifs located on GD and FT domains respectively activated only 4 proteins with very little commonality with the other antibody treatments.

Differential expression of FcεR1a was observed following DAT and DMT with ICSM35 antibody treatment of MPN and mouse neuroblastoma cells. However, FcεR1a overexpression was only observed following DAT of MPN with ICSM35 but not ICSM18, suggesting that ICSM35 binding to PrP^C^ FT domain stimulates FcεR1 probably *via* binding to FcγR. Co-localisation of PPARD with FcεR1 following treatment with ICSM35 further confirms the allergenic phenomenon induced by the anti-PrP antibody treatment. A recent study by Moon and co-workers have shown that a PPARγ agonist (troglitazone) is able to weaken PrP−mediated neurotoxicity in primary neuronal cells *via* PPARγ signal activation and autophagy flux inhibition (147).

Antibody-mediated therapy for prions attracted intense debate and controversy as some of the reported were contradictory partly because these relied on a somewhat superficial assessment using microscopy and also due to the failure of investigating the fine molecular events caused by cross-lining PrP^C^ with anti-PrP antibodies. Luckily, this controversy related to prion antibody treatment did not lead to fatalities in humans affected with CJD. However, Alzheimer’s disease trials have led the death of individuals administered with therapeutic antibodies. This study led to a unique discovery showing that anti-PrP antibodies led to neuronal allergenicity *via* different pathways but also highlights the key role played by microglia in causing the allergenic reaction. This study also emphasizes the need to include a screening ‘allergenicity’ step during development of therapeutic antibodies to avoid potential side-effects.

## Data Availability Statement

The raw data supporting the conclusions of this article will be made available by the authors, without undue reservation.

## Author Contributions

UKA performed experiments and wrote draft manuscript, ES, XZ, UH, SK, MM and MS performed experiments; CGL, GJG, LO, MAD, SC, and TK. reviewed manuscript; MT designed, managed, wrote and revised manuscript. All authors contributed to the article and approved the submitted version.

## Funding

This work was supported by an Ainsworth Medical Research Innovation Fund Grant awarded to MT. TK received funding from two project grants from the National Health and Medical Research Council [#1102012 and #1141789] and the NHMRC dementia research team initiative [#1095215] as well as the Ainsworth Medical Research Innovation Fund and the Australian Research Council [#DP18010473].

## Conflict of Interest

The authors declare that the research was conducted in the absence of any commercial or financial relationships that could be construed as a potential conflict of interest.

## Publisher’s Note

All claims expressed in this article are solely those of the authors and do not necessarily represent those of their affiliated organizations, or those of the publisher, the editors and the reviewers. Any product that may be evaluated in this article, or claim that may be made by its manufacturer, is not guaranteed or endorsed by the publisher.
