# Supplementary material for: Cross-Linking Cellular Prion Protein Induces Neuronal Type 2-Like Hypersensitivity
Source: Front Immunol. 2021 Jul 30;12:639008. doi: 10.3389/fimmu.2021.639008 (PMC8361482; doi:10.3389/fimmu.2021.639008)
Supplement: Supplementary file 1 [file DataSheet_1.pdf]

Supplementary Files

S1. Supplementary methods: Methods related to the predictions of the 3-dimensional structure of the human PrP^{C}; prediction of linear and conformational B cell epitopes; and prediction of toxicity and allergenicity of the linear B cell epitopes.

Prediction and Validation of the Three-dimensional Structure of the Human Major Prion Protein: The I-TASSER server (https://zhanglab.ccmb.med.umich.edu/I-TASSER/) (1) was used to predict the three-dimensional (3D) structure of the full-length (253 amino acid) huPrP. I-TASSER is a hierarchical template-based protein three-dimensional structure prediction server which uses the query protein sequence and predicts the structure through multiple threading alignments, iterative structure assembly simulations, and comparative functional modelling approaches using state-of-art algorithms (1). The best protein model can be selected based on the template modelling score (TM-score), confidence score (C-score), and root mean square deviation (RMSD) score (1). The predicted structure was visualized using the PyMOL v2.3 (The PyMOL Molecular Graphics System, Version 2.3 Schrödinger, LLC.).
The predicted 3D structure was primarily evaluated based on the Ramachandran plot (2) in PROCHECK (3) that shows mainly most favoured region, additionally allowed regions, and the disallowed regions of the protein structure that was predicted by the PDBsum server (http://www.ebi.ac.uk/pdbsum) (3). The protein 3D model was further assessed by the ProSA (https://prosa.services.came.sbg.ac.at/prosa.php), a web server that can recognize the errors of the theoretical protein model and calculates the overall quality of the protein 3D model (4). The SAVES v5.0 server (https://servicesn.mbi.ucla.edu/SAVES/) was used for the Verify3D score of the protein model (5). Verify3D determines the compatibility of an atomic model (3D) with its own amino acid sequence (1D) by assigning a structural class based on its location and environment (alpha, beta, loop, polar, nonpolar) and comparing the results to good structures (5).

Prediction of Linear and conformational B Cell epitopes in the Human Major Prion Protein: The linear and conformational or discontinuous B Cell epitopes in the huPrP 3D structure were predicted using the Ellipro server (http//tools.iedb.org/ellipro/) (6). Ellipro is a web-server which uses the geometrical properties of the protein structure in combination with MODELLER program and residue clustering algorithm for the prediction of the B Cell epitopes in the protein region protruding from the protein's globular surface (6). We used the default parameters (minimum score 0.5 and maximum distance 0.6Å) of the Ellipro server for the prediction of both linear and conformational B Cell epitopes.

Prediction of the Toxicity and Allergenicity of the Linear B Cell epitopes: We used ToxinPred server (http://crdd.osdd.net/raghava/toxinpred/) (7) for the prediction of toxic/non-toxic nature of the linear B Cell epitopes identified the Ellipro server as described above. We used both the support vector machine (SVM) and quantitative matrix (QM) method in ToxinPred server for non-toxic epitope selection. The ToxinPred server has been developed based on the QM and machine learning technique using different properties of the peptides for the prediction of toxicity or non-toxicity of the peptides with 93.92% and 88.00% accuracy in SVM and QM methods, respectively. This server can also be used to identify the most toxic regions in the protein sequence (7).
For the allergenicity prediction, we used *k* nearest neighbours (kNN) method based server AllerTOP v2.0 (http://www.ddg-pharmfac.net/AllerTOP/) (8) and a novel alignment-free descriptor-based fingerprint approach server AllergenFP v1.0 (http://www.ddg-pharmfac.net/AllergenFP/) (9). The AllergenFP v1.0 server predicts the allergenicity and non-

allergenicity with an accuracy of 88.00% by analysing the key amino acid features such as β-strand, helix, hydrophobicity forming propensities, size, and relative abundance of the amino acids (9). Further, AllerTOP v2.0 server shows 88.7% overall accuracy and uses auto and cross-covariance, machine learning approach and a *k*NN method for the classification of allergen and non-allergen proteins and peptides (8).

References

1. Roy A, Kucukural A, Zhang Y. I-TASSER: a unified platform for automated protein structure and function prediction. *Nat Protoc* (2010) **5**:725—738. doi:10.1038/nprot.2010.5

2. RAMACHANDRAN GN, RAMAKRISHNAN C, SASISEKHARAN V. Stereochemistry of polypeptide chain configurations. *J Mol Biol* (1963) **7**:95–99. doi:10.1016/s0022-2836(63)80023-6

3. Laskowski RA, Jabłońska J, Pravda L, Vařeková RS, Thornton JM. PDBsum: Structural summaries of PDB entries. *Protein Sci* (2018) **27**:129–134. doi:10.1002/pro.3289

4. Wiederstein M, Sippl MJ. ProSA-web: interactive web service for the recognition of errors in three-dimensional structures of proteins. *Nucleic Acids Res* (2007) **35**:W407—10. doi:10.1093/nar/gkm290

5. Eisenberg D, Lüthy R, Bowie JU. VERIFY3D: assessment of protein models with three-dimensional profiles. *Methods Enzymol* (1997) **277**:396–404. doi:10.1016/S0076-6879(97)77022-8

6. Ponomarenko J, Bui H-H, Li W, Fusseder N, Bourne PE, Sette A, Peters B. ElliPro: a new structure-based tool for the prediction of antibody epitopes. *BMC Bioinformatics* (2008) **9**:514. doi:10.1186/1471-2105-9-514

7. Gupta S, Kapoor P, Chaudhary K, Gautam A, Kumar R, Open Source Drug Discovery Consortium, Raghava GPS. In silico approach for predicting toxicity of peptides and proteins. *PLoS One* (2013) **8**:e73957. doi:10.1371/journal.pone.0073957

8. Dimitrov I, Bangov I, Flower DR, Doytchinova I. AllerTOP v.2 - A server for in silico prediction of allergens. *J Mol Model* (2014) **20**: doi:10.1007/s00894-014-2278-5

9. Dimitrov I, Naneva L, Doytchinova I, Bangov I. AllergenFP: Allergenicity prediction by descriptor fingerprints. *Bioinformatics* (2014) **30**:846–851. doi:10.1093/bioinformatics/btt619

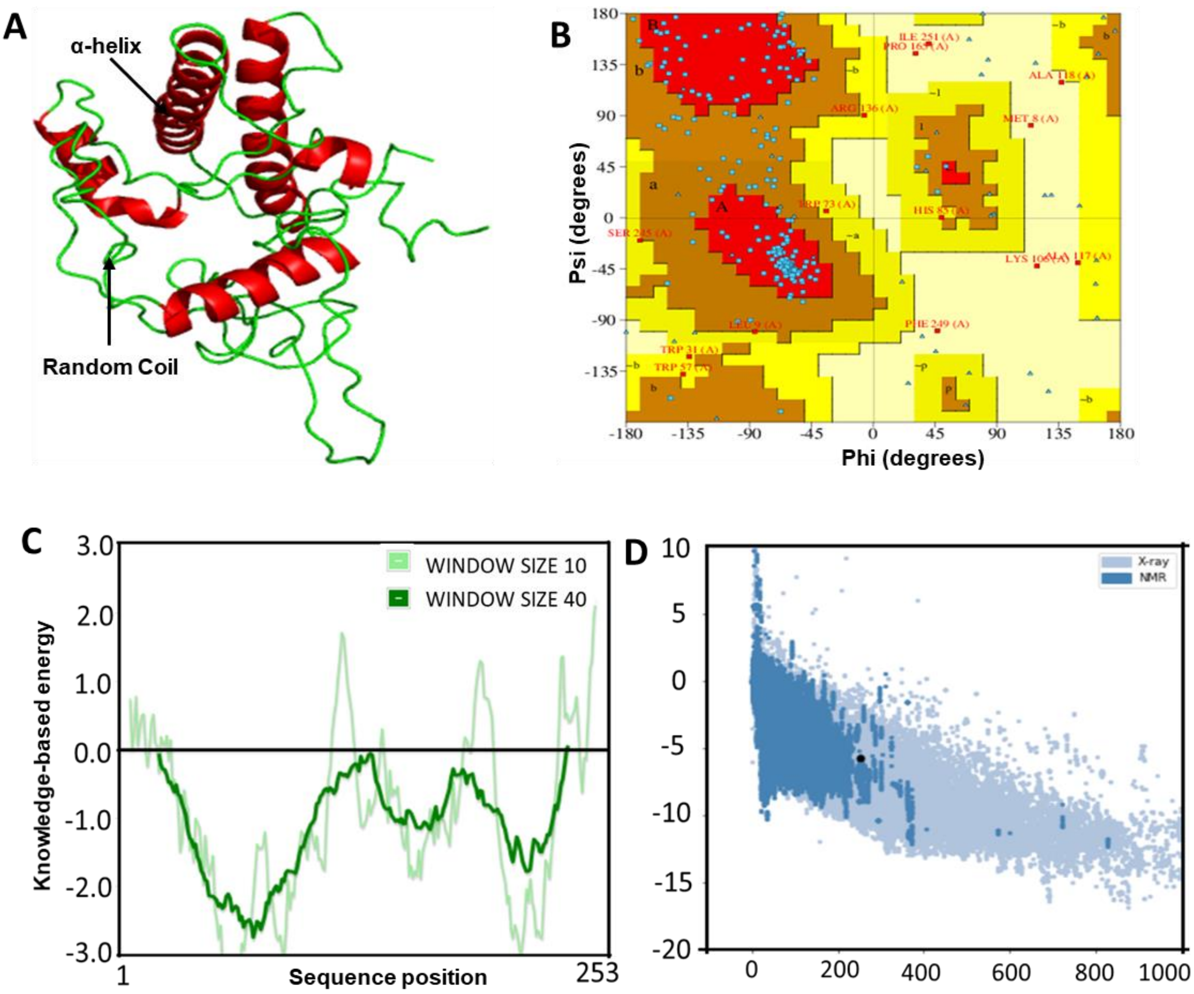

Figure S1: Evaluation of the predicted protein three-dimensional structure of the human major prion protein (huPrP). A) three-dimensional structure of the huPrP which is enriched with alpha-helix and random coils B) Ramachandran plot for the predicted three-dimensional structure of the huPrP (68.4% residues in most favoured region, 24.7% in allowed regions, 3.7% in generously allowed regions, and 3.2% residues in the disallowed regions) indicating the good quality of the protein structure C) ProSA energy plot for the predicted protein model. Herein 0.0 is the baseline, the sequence position under the baseline (0.0) indicates the good quality of the protein model. D) ProSA Z-score of the predicted huPrP three-dimensional structure where the black dot in that position indicates the good quality of the protein 3D structure.

Table S1: Properties of the identified proteins following direct antibody treatment (DAT) of the neuroblastoma cell line. The properties were identified by Progenesis Software after the LC-MS analysis. Selected 26 proteins have been highlighted as bold.

Accession	Peptides	Unique peptides	Confidence score	Anova (p)	q Value	Max fold change	Highest Mean	Lowest Mean	Description
A0A0A6YY04	5	1	31.8	0.0006	0.514327	α	CTL	DAT	Nuclear receptor subfamily 5 group A member 2 GN=Nr5a2
A0A0B4J1P1	1	1	5.19	0.00526	0.514327	545	CTL	DAT	T cell receptor beta_ variable 30 (Fragment) GN=Trbv30
A0A0G2JFJ8	1	1	5.79	0.021708	0.720313	38.8	CTL	DAT	SWI/SNF complex subunit SMARCC1 (Fragment) GN=Smarcc1
A0A1D5RLY9	2	1	11.5	0.040093	0.892186	α	CTL	DAT	Signal-induced proliferation-associated 1-like protein 2 GN=Sipa1l2
**A0A2I3BPI0**	**10**	**2**	**55.9**	**0.043282**	**0.923819**	**33.2**	**CTL**	**DAT**	**Coiled-coil domain-containing protein 187 GN=Ccdc187**
A0A140LHI9	1	1	5.5	0.006527	0.514327	42.5	CTL	DAT	Sodium- and chloride-dependent glycine transporter 2 GN=Slc6a5
A0A140LIX9	3	1	15.9	0.002447	0.514327	13.1	CTL	DAT	Cilia and flagella-associated protein 99 GN=Cfap99
A2AM95	1	1	5.31	0.035641	0.884384	12.6	CTL	DAT	Emerin GN=Emd
B0QZL5	6	1	40.8	0.000559	0.514327	α	CTL	DAT	Phosphatidylinositol 4_5-bisphosphate 3-kinase catalytic subunit GN=Pik3cd
**B2RUM8**	**10**	**4**	**57.5**	**0.008377**	**0.57741**	**20**	**CTL**	**DAT**	**RNA helicase GN=Ddx18**
B2RUQ2	3	1	14.1	0.040093	0.892186	α	CTL	DAT	Gene model 608_ (NCBI) GN=Usf3
B9EKD9	3	2	15.3	0.047213	0.942414	22.6	CTL	DAT	Unkempt-like (Drosophila) GN=Unkl
D3YZZ6	4	1	29.6	0.018967	0.714122	167	CTL	DAT	Eukaryotic translation initiation factor 2A (Fragment) GN=Eif2a
D3Z4N0	42	1	239	0.016418	0.712925	13.9	CTL	DAT	Pleckstrin homology-like domain family B member 1 GN=Phldb1
D3Z061	3	1	16.9	0.040093	0.892186	α	CTL	DAT	Ubiquitin-like modifier-activating enzyme 6 GN=Uba6
E0CY42	2	1	9.48	0.040093	0.892186	α	CTL	DAT	Chloride intracellular channel protein 6 (Fragment) GN=Clic6
E9PWB1	42	1	237	0.016417	0.712925	13.9	CTL	DAT	Pleckstrin homology-like domain family B member 1 GN=Phldb1
E9QK48	4	1	21.4	0.040093	0.892186	α	CTL	DAT	Echinoderm microtubule-associated protein-like 2 GN=Eml2
G3UXY0	8	1	56.6	0.040093	0.892186	α	CTL	DAT	Proteasome activator complex subunit 1 GN=Psme1
J3QPG5	27	1	213	0.033712	0.878143	16.5	CTL	DAT	Prosaposin GN=Psap
P05555	4	1	22.9	0.002871	0.514327	15	CTL	DAT	Integrin alpha-M GN=Itgam
**P19426**	**20**	**5**	**117**	**0.003643**	**0.514327**	**12.3**	**CTL**	**DAT**	**Negative elongation factor E GN=Nelfe**
P61027	10	1	79.2	0.012547	0.659912	11.2	CTL	DAT	Ras-related protein Rab-10 GN=Rab10
P70320	4	1	23	0.040242	0.892186	1.93E+04	CTL	DAT	Tousled-like kinase GN=Tlk2
P70404	2	1	17	0.000851	0.514327	4.63E+03	CTL	DAT	Isocitrate dehydrogenase [NAD] subunit gamma 1_ mitochondrial GN=Idh3g
Q3TDV6	2	1	10.8	0.017986	0.714122	79.1	CTL	DAT	Uncharacterized protein GN=Mad1l1
Q3TLH6	15	1	179	0.025065	0.770252	21.7	CTL	DAT	FABP domain-containing protein GN=Fabp5
Q3TNE8	1	1	6.01	0.010298	0.612225	12.9	CTL	DAT	D-aspartate oxidase GN=Ddo
Q3UEK5	8	1	56.1	0.014589	0.681384	138	CTL	DAT	Uncharacterized protein GN=Ahsg
Q5FW97	63	1	848	0.008469	0.57741	11.8	CTL	DAT	Enolase 1_ alpha non-neuron GN=EG433182
Q8BHS6	3	2	21.8	0.002646	0.514327	52.7	CTL	DAT	Armadillo repeat-containing X-linked protein 3 GN=Armcx3
Q8CDS3	4	1	28.6	0.040093	0.892186	α	CTL	DAT	DUF4629 domain-containing protein GN=4930433I11Rik
Q8CF78	34	1	421	0.008469	0.57741	11.8	CTL	DAT	Uncharacterized protein
Q9D4H2	5	1	24.1	0.042914	0.923819	27.4	CTL	DAT	GRIP and coiled-coil domain-containing protein 1 GN=Gcc1
Q9QY83	1	1	5.99	0.040093	0.892186	α	CTL	DAT	Actin-like protein 7B GN=Actl7b
Q78JN3	4	1	27.6	0.001244	0.514327	10.2	CTL	DAT	Enoyl-CoA delta isomerase 3_ peroxisomal GN=Eci3
Q91V48	3	2	13.6	0.00125	0.514327	23.3	CTL	DAT	Protocadherin beta 21 GN=Pcdhb21
**Q497U2**	**20**	**2**	**107**	**0.021997**	**0.720313**	**20.3**	**CTL**	**DAT**	**Pcnt protein (Fragment) GN=Pcnt**
**Q05816**	**22**	**3**	**260**	**0.01719**	**0.714122**	**16**	**CTL**	**DAT**	**Fatty acid-binding protein 5 GN=Fabp5**
Q60737	2	1	16.1	0.01737	0.714122	17.9	CTL	DAT	Casein kinase II subunit alpha GN=Csnk2a1
A0A0G2JDP0	1	1	4.41	0.044814	0.936178	46.6	DAT	CTL	HCLS1-associated protein X-1 GN=Hax1
**A0A0N4SVL0**	**15**	**3**	**88.3**	**0.004893**	**0.514327**	**14.1**	**DAT**	**CTL**	**Eukaryotic translation initiation factor 4 gamma 3 GN=Eif4g3**
A0A0N5E9G7	3	2	12.9	0.031157	0.846738	12.6	DAT	CTL	Replication factor C subunit 1 GN=Rfc1
A0A0R4J0G9	1	1	5.47	0.038889	0.892186	1.53E+03	DAT	CTL	Methionine synthase reductase GN=Mtrr
A0A0R4J046	4	1	21.8	0.032966	0.872172	15.7	DAT	CTL	CCAAT/enhancer-binding protein zeta GN=Cebpz
A0A1B0GSZ7	1	1	4.75	0.022596	0.722164	16.1	DAT	CTL	Tetraspanin GN=Cd37
A0A1C7ZMY0	1	1	4.75	0.006459	0.514327	14.6	DAT	CTL	Neuromedin-U receptor 1 GN=Nmur1 PE=3 SV=2
A0A1G5SJ32	1	1	10.9	0.017103	0.714122	α	DAT	CTL	NLR family_ pyrin domain containing 1A_ CAST/EiJ strain specific GN=OTTMUSCASG00059399
A0A2I3BRC7	1	1	5.98	0.002212	0.514327	470	DAT	CTL	Nuclear receptor coactivator 4 GN=Ncoa4
**A0A2R8VHP3**	**12**	**2**	**91.5**	**0.003933**	**0.514327**	**267**	**DAT**	**CTL**	**Predicted pseudogene 5478 GN=Gm5478**

A0A4X8	6	3	37.8	0.022592	0.722164	1.19E+03	DAT	CTL	Uncharacterized protein GN=Kirrel2
A0A087WPN2	1	1	9.78	0.01951	0.714122	1.05E+03	DAT	CTL	Zinc finger protein GN=Gm3604
A0A087WRM0	3	1	23.7	0.000956	0.514327	48.8	DAT	CTL	Protein CDV3 (Fragment) GN=Cdv3
A2A6Q8	2	1	11.1	0.040422	0.892186	11.8	DAT	CTL	Myosin light chain 4 (Fragment) GN=Myl4
**A2A8L5**	**18**	**5**	**102**	**0.006077**	**0.514327**	**11.9**	**DAT**	**CTL**	**Receptor-type tyrosine-protein phosphatase F GN=Ptprf**
A2AHM2	2	1	20.6	0.014154	0.681384	39.8	DAT	CTL	Zinc finger protein 931 GN=Zfp931
**A2RSV8**	**8**	**2**	**60.5**	**0.002291**	**0.514327**	**17.9**	**DAT**	**CTL**	**Cytochrome c oxidase subunit IV isoform 1 GN=Cox4i1**
**A2RSY1**	**11**	**3**	**74.2**	**0.030784**	**0.842352**	**13.7**	**DAT**	**CTL**	**KAT8 regulatory NSL complex subunit 3 GN=Kansl3**
**A4FUV6**	**20**	**7**	**130**	**0.003153**	**0.514327**	**23.4**	**DAT**	**CTL**	**Met protein (Fragment) GN=Met**
**A7E215**	**7**	**3**	**42.8**	**0.035183**	**0.883997**	**18**	**DAT**	**CTL**	**Rps6ka3 protein (Fragment) GN=Rps6ka3**
**B1AZR7**	**9**	**4**	**60.8**	**0.006661**	**0.514327**	**11.7**	**DAT**	**CTL**	**Protocadherin 11 X-linked GN=Pcdh11x**
B1GX81	8	3	37.7	0.022569	0.722164	11.2	DAT	CTL	PAK3cb protein GN=Pak3
B9EHZ5	3	1	15.8	0.002389	0.514327	13.5	DAT	CTL	Membrane protein_ palmitoylated 6 (MAGUK p55 subfamily member 6) GN=Mpp6
D3YYX0	3	1	15.2	0.014416	0.681384	10.4	DAT	CTL	Thioredoxin-related transmembrane protein 2 (Fragment) GN=Tmx2
D3YZ62	32	1	179	0.001589	0.514327	66.7	DAT	CTL	Unconventional myosin-Va GN=Myo5a
D3Z2G0	1	1	6.27	0.015175	0.69075	42.4	DAT	CTL	Ankyrin repeat domain 29 (Fragment) GN=Ankrd29
D3Z4S3	1	1	4.22	0.019286	0.714122	152	DAT	CTL	Putative peptidyl-tRNA hydrolase PTRHD1 GN=Ptrhd1
**E9QKD1**	**12**	**2**	**74.5**	**0.048564**	**0.942414**	**201**	**DAT**	**CTL**	**Nucleolar protein 8 GN=Nol8**
F6VQX8	13	1	61.9	0.015894	0.712925	α	DAT	CTL	Predicted gene 20547 (Fragment) GN=Gm20547
F6WAU7	2	1	9.57	0.019483	0.714122	α	DAT	CTL	Zinc finger protein 945 GN=Zfp945
F6XN97	1	1	9.82	0.018875	0.714122	22.4	DAT	CTL	RIKEN cDNA 0610010K14 gene GN=0610010K14Rik
F6YZ61	3	1	20.8	0.00734	0.553291	18.2	DAT	CTL	Coiled-coil and C2 domain-containing protein 2A (Fragment) GN=Cc2d2a
F7BTP0	1	1	5.38	0.006278	0.514327	α	DAT	CTL	Non-homologous end-joining factor 1 (Fragment) GN=Nhej1
G5E8E1	17	1	108	0.008067	0.57741	11.3	DAT	CTL	Leucine-rich repeat flightless-interacting protein 1 GN=Lrrfip1
G5E852	4	1	19.9	0.005488	0.514327	11.3	DAT	CTL	Tyrosine-protein kinase GN=Jak2
H7BWX6	1	1	5.87	0.028547	0.834895	α	DAT	CTL	CCR4-NOT transcription complex subunit 2 GN=Cnot2
**H7BX49**	**13**	**3**	**73.6**	**0.041295**	**0.901501**	**16.1**	**DAT**	**CTL**	**WD repeat-containing protein 90 GN=Wdr90**
J3S6Y1	2	1	16.5	0.036343	0.892186	32.2	DAT	CTL	Thrombospondin-type laminin G domain and EAR repeat-containing protein GN=Tspear
O55111	5	1	24.5	0.020868	0.714122	12	DAT	CTL	Desmoglein-2 GN=Dsg2
P13439	2	1	17.4	0.029442	0.840688	10.1	DAT	CTL	Uridine 5'-monophosphate synthase GN=Umps
P19157	8	1	86	0.047941	0.942414	α	DAT	CTL	Glutathione S-transferase P 1 GN=Gstp1
P61460	6	2	32.5	0.019541	0.714122	18.5	DAT	CTL	GATOR complex protein DEPDC5 GN=Depdc5
P70313	4	2	23.4	0.013434	0.680528	260	DAT	CTL	Nitric oxide synthase_ endothelial GN=Nos3
Q0VBN2	6	1	33.6	0.012107	0.659912	38.9	DAT	CTL	Dermatan-sulfate epimerase-like protein GN=Dsel
**Q3TW28**	**31**	**9**	**171**	**0.004071**	**0.514327**	**111**	**DAT**	**CTL**	**Uncharacterized protein GN=Tpp2**
Q3U0E6	1	1	10.3	0.048831	0.942414	54.8	DAT	CTL	Uncharacterized protein
Q3UAF1	9	1	73.4	0.037821	0.892186	17.5	DAT	CTL	Uncharacterized protein GN=Ddx39
**Q3UBP6**	**58**	**3**	**802**	**0.018806**	**0.714122**	**12.3**	**DAT**	**CTL**	**Uncharacterized protein GN=Actb**
**Q3UR03**	**7**	**3**	**49.1**	**0.020842**	**0.714122**	**29.9**	**DAT**	**CTL**	**Myomegalin (Fragment) GN=Pde4dip**
Q3V277	6	1	34.5	0.018635	0.714122	16	DAT	CTL	Uncharacterized protein GN=Zbtb1
**Q5U430**	**10**	**3**	**53.5**	**0.005487**	**0.514327**	**65.4**	**DAT**	**CTL**	**E3 ubiquitin-protein ligase UBR3 GN=Ubr3**
Q6P5U7	6	3	32.2	0.005966	0.514327	96.1	DAT	CTL	NACHT and WD repeat domain-containing protein 2 GN=Nwd2
**Q6PDI5**	**25**	**6**	**142**	**0.007494**	**0.554431**	**11.1**	**DAT**	**CTL**	**Proteasome adapter and scaffold protein ECM29 GN=Ecpas**
Q6PE66	2	1	10	0.048515	0.942414	α	DAT	CTL	Phosphorylase b kinase regulatory subunit GN=Phka2
Q8BJL0	3	2	14.4	0.021422	0.719171	11.1	DAT	CTL	SWI/SNF-related matrix-associated actin-dependent regulator of chromatin subfamily A-like protein 1 GN=Smarcal1
Q8BN58	7	1	35	0.042906	0.923819	243	DAT	CTL	Rho GTPase-activating protein 28 GN=Arhgap28
Q8BV20	3	1	23.4	0.014183	0.681384	13.3	DAT	CTL	Uncharacterized protein GN=Rhobtb2
Q8K368	4	2	20.4	0.006121	0.514327	α	DAT	CTL	Fanconi anemia group I protein homolog GN=Fanci
**Q8K442**	**10**	**2**	**60**	**0.004472**	**0.514327**	**12.7**	**DAT**	**CTL**	**ATP-binding cassette sub-family A member 8-A GN=Abca8a**
Q8R464	3	2	22.2	0.018245	0.714122	22.1	DAT	CTL	Cell adhesion molecule 4 GN=Cadm4
Q8VDK4	3	1	16	0.00398	0.514327	15.4	DAT	CTL	Cadherin 13 GN=Cdh13
Q9CZJ2	4	1	21.8	0.026437	0.805263	14.9	DAT	CTL	Heat shock 70 kDa protein 12B GN=Hspa12b
Q9ERC8	8	4	35.3	0.001969	0.514327	73.8	DAT	CTL	Down syndrome cell adhesion molecule homolog GN=Dscam
**Q9WUM5**	**12**	**4**	**68.1**	**0.04593**	**0.936178**	**17.1**	**DAT**	**CTL**	**Succinate--CoA ligase [ADP/GDP-forming] subunit alpha_ mitochondrial GN=Suclg1**
Q80T69	5	1	24.2	0.020869	0.714122	23	DAT	CTL	Lysine-specific demethylase 9 GN=Rsbn1
**Q80XC6**	**9**	**2**	**46.5**	**0.048742**	**0.942414**	**28.8**	**DAT**	**CTL**	**Nuclear exosome regulator NRDE2 GN=Nrde2**
Q91VN1	2	1	11.8	0.01209	0.659912	295	DAT	CTL	Zinc finger protein 24 GN=Znf24
Q91XY5	4	1	19.9	0.03448	0.879244	12.2	DAT	CTL	Protocadherin gamma A3 GN=Pcdhga3
**Q99JW2**	**10**	**2**	**62.7**	**0.021118**	**0.714964**	**α**	**DAT**	**CTL**	**Aminoacylase-1 GN=Acy1**
**Q499M4**	**6**	**2**	**45.2**	**0.010421**	**0.612225**	**10.7**	**DAT**	**CTL**	**Tigger transposable element derived 5 GN=Tigd5**

Q544Y3	4	1	21.6	0.028631	0.834895	12.3	DAT	CTL	CRAL-TRIO domain-containing protein GN=Rlbp1
**Q02819**	**10**	**4**	**52**	**0.044479**	**0.935234**	**33.1**	**DAT**	**CTL**	**Nucleobindin-1 GN=Nucb1**

Table S2: Properties of the identified proteins following direct antibody treatment (DAT) of the mouse primary neuron (MPN) in comparison with untreated cells. The properties were identified by Progenesis Software after the LC-MS analysis.

Accession	Gene ID	Peptides	Unique peptides	Confidence score	Anova (p)	q Value	Max fold change	Highest Mean	Lowest Mean	Description
O70291	Grk4	3	1	19.1	1.28E-05	0.001518	1.42 E+03	NOT	DAT	G protein-coupled receptor kinase 4 GN=Grk4
A6H5U5	Lrrfip1	5	2	26.6	2.07E-05	0.001931	3.03	NOT	DAT	Lrrfip1 protein GN=Lrrfip1
B2RU82	Slc30a9	4	2	23.5	3.57E-05	0.002914	7.03	NOT	DAT	Solute carrier family 30 (Zinc transporter)_ member 9 GN=Slc30a9
Q8BNV9	Hoxc8	2	2	15.7	4.46E-05	0.003232	14.5	NOT	DAT	Homeobox domain-containing protein (Fragment) GN=Hoxc8
A0A5F8MPF0	Kif1b	5	1	31.4	5.10E-05	0.003328	106	DAT	NOT	Kinesin-like protein KIF1B GN=Kif1b
P56480	Atp5f1b	26	19	213	5.81E-05	0.003442	6.63	NOT	DAT	ATP synthase subunit beta_ mitochondrial GN=Atp5f1b
A2A4A6	Plcg1	8	8	42.4	7.54E-05	0.003611	1.86	DAT	NOT	1-phosphatidylinositol 4_5-bisphosphate phosphodiesterase gamma GN=Plcg1
A0A0R4J0A4	Flt1	4	4	15.6	7.59E-05	0.003611	5.69	NOT	DAT	Vascular endothelial growth factor receptor 1 GN=Flt1
A2RTV8	Rln1	3	2	21.6	8.31E-05	0.003611	5.22	NOT	DAT	Prorelaxin 1 GN=Rln1
B1AXW5	Prdx1	8	6	60.2	9.35E-05	0.003809	2.44	NOT	DAT	Peroxiredoxin-1 (Fragment) GN=Prdx1
Q8VC94	Rpl11	4	1	24	0.000106	0.004072	5.93	NOT	DAT	Rpl11 protein GN=Rpl11
A0A571BEW5	Lpar2	3	2	19.3	0.000114	0.004136	10.6	DAT	NOT	Lysophosphatidic acid receptor 2 GN=Lpar2
A0A5F8MPM7	-	3	2	15.4	0.000152	0.005107	4.32	NOT	DAT	Cingulin GN=Cgn
Q99KI0	Aco2	13	11	74	0.000167	0.005107	2.15	DAT	NOT	Aconitate hydratase_ mitochondrial GN=Aco2
A0A571BEG7	Patj	3	2	17.9	0.000176	0.005107	2.6	NOT	DAT	InaD-like protein GN=Patj
Q8VHU9	-	4	1	28.4	0.000204	0.005552	193	NOT	DAT	Eukaryotic translation initiation factor 5A (Fragment)
P03995	Gfap	6	4	44.9	0.00024	0.005963	4	NOT	DAT	Glial fibrillary acidic protein GN=Gfap
P15864	H1-2	8	1	98	0.000247	0.005963	4.84	NOT	DAT	Histone H1.2 GN=H1-2
Q8VDD5	Myh9	16	10	94.9	0.000437	0.008407	2.28	NOT	DAT	Myosin-9 GN=Myh9
A0A0U1RNK7	Dock7	8	4	58.2	0.000438	0.008407	12.3	NOT	DAT	Dedicator of cytokinesis protein 7 GN=Dock7
A0A0R4J1E2	Eef1d	4	2	27.4	0.000509	0.009159	4.78	NOT	DAT	Elongation factor 1-delta GN=Eef1d
B9EIW5	Ncoa7	13	9	96.5	0.00052	0.009159	1.96	DAT	NOT	Ncoa7 protein GN=Ncoa7
P43274	H1-4	10	3	115	0.000597	0.00973	2.73	NOT	DAT	Histone H1.4 GN=H1-4
Q8BVV3	Pknox2	3	2	14.9	0.00062	0.009856	11.2	NOT	DAT	Meis_PKNOX_N domain-containing protein GN=Pknox2
Q920P5	Ak5	3	3	17.2	0.000903	0.01306	1.61	DAT	NOT	Adenylate kinase isoenzyme 5 GN=Ak5
Q8BK67	Rcc2	5	3	28.4	0.000918	0.01306	4.56	NOT	DAT	Protein RCC2 GN=Rcc2
E9Q3I8	Itsn1	14	1	81.7	0.000921	0.01306	1.7	DAT	NOT	Intersectin-1 GN=Itsn1
Q5I2A0	Serpina3g	5	5	21.9	0.001085	0.014152	8.49	DAT	NOT	Serine protease inhibitor A3G GN=Serpina3g
B1ARR7	Eno1	15	2	188	0.001199	0.015032	2.09	NOT	DAT	Alpha-enolase (Fragment) GN=Eno1
P62918	Rpl8	5	5	49.1	0.001225	0.015065	8.33	NOT	DAT	60S ribosomal protein L8 GN=Rpl8
Q571K1	Golgb1	7	4	30	0.001304	0.015223	11.2	NOT	DAT	MKIAA4151 protein (Fragment) GN=Golgb1
Q58E64	Eef1a1	22	2	253	0.001306	0.015223	6.07	NOT	DAT	Elongation factor 1-alpha GN=Eef1a1
A0A2R8VHH1	Nup155	5	2	31.3	0.001364	0.015603	4.68	NOT	DAT	Nuclear pore complex protein Nup155 GN=Nup155
Q52KG5	Kif26a	4	1	27.1	0.001557	0.017387	13.6	NOT	DAT	Kinesin-like protein KIF26A GN=Kif26a
Q6KAM8	Flna	5	2	28.2	0.001573	0.017387	4.25	NOT	DAT	MFLJ00343 protein (Fragment) GN=Flna
B7ZP22	Hnrnpa2b1	11	10	74.8	0.001645	0.017875	2.03	DAT	NOT	Heterogeneous nuclear ribonucleoprotein A2/B1 GN=Hnrnpa2b1
P40142	Tkt	24	6	199	0.001711	0.018215	2.02	DAT	NOT	Transketolase GN=Tkt
A0A1S6GWJ8	Hnrnpm	2	1	15.3	0.001839	0.018732	21	DAT	NOT	Uncharacterized protein GN=Hnrnpm
S4R2B0	Rbm25	17	10	91.1	0.00206	0.020553	1.2	DAT	NOT	RNA-binding protein 25 (Fragment) GN=Rbm25

Q6DFZ2	Syne2	3	2	23.3	0.002138	0.020553	4.13	NOT	DAT	Nesprin-2 (Fragment) GN=Syne2
Q9D9Y2		15	5	106	0.002336	0.021117	11.6	NOT	DAT	Uncharacterized protein
Q3UEM8	Hspa5	22	1	208	0.002391	0.021117	1.93	NOT	DAT	Uncharacterized protein (Fragment) GN=Hspa5
Q3TI47	Hspa5	25	2	226	0.002391	0.021117	1.93	NOT	DAT	Uncharacterized protein GN=Hspa5
Q3UHN1	Slit3	4	3	17	0.002397	0.021117	4.55	DAT	NOT	Uncharacterized protein GN=Slit3
Q149Z9	H1f3	12	3	121	0.00255	0.021875	1.61	NOT	DAT	Histone cluster 1_ H1d GN=H1f3
Q6P1E8	Efcab6	7	5	41.6	0.002651	0.022159	1.62	NOT	DAT	EF-hand calcium-binding domain-containing protein 6 GN=Efcab6
Q3U900	Rpn1	5	4	36	0.002952	0.024362	7.23	NOT	DAT	Dolichyl-diphosphooligosaccharide--protein glycosyltransferase subunit 1 GN=Rpn1
F6ZSB7	Phgdh	9	3	62.7	0.003123	0.025453	2.07	NOT	DAT	D-3-phosphoglycerate dehydrogenase (Fragment) GN=Phgdh
A2A841	Epb41	5	4	35.5	0.003273	0.025595	2.28	DAT	NOT	Protein 4.1 GN=Epb41
Q8BFZ3	Actbl2	16	5	158	0.003283	0.025595	3.21	DAT	NOT	Beta-actin-like protein 2 GN=Actbl2
Q3U741	Ddx17	6	4	33.9	0.003315	0.025595	1.4	DAT	NOT	Probable ATP-dependent RNA helicase DDX17 GN=Ddx17
Q3UZQ3	Eef1a1	23	3	238	0.003475	0.025924	5.08	NOT	DAT	Elongation factor 1-alpha GN=Eef1a1
P19426	Nelfe	19	5	97.7	0.003499	0.025924	1.49	DAT	NOT	Negative elongation factor E GN=Nelfe
Q6PCM4	Zfp157	3	2	17.8	0.003979	0.028699	1.62	NOT	DAT	Zinc finger protein 157 GN=Zfp157
A0A140LI36	Adgb	14	13	62.3	0.004005	0.028699	1.52	NOT	DAT	Androglobin GN=Adgb
A4GZ26	Iqsec2	5	4	26.8	0.004831	0.032866	35.3	NOT	DAT	ARF6 guanine nucleotide exchange factor IQArfGEF GN=Iqsec2
B2RY51	Trip11	14	9	76	0.004839	0.032866	2.34	DAT	NOT	Thyroid hormone receptor interactor 11 GN=Trip11
Q4KL76	Hspe1	7	7	79.7	0.00557	0.036686	1.77	NOT	DAT	Heat shock protein 1 (Chaperonin 10) GN=Hspe1
G3V011	Myo1f	7	7	33.8	0.005817	0.037927	1.85	NOT	DAT	Unconventional myosin-If GN=Myo1f
P21550	Eno3	10	3	98.1	0.005908	0.038141	3.21	NOT	DAT	Beta-enolase GN=Eno3
A0A2R8W6H9	Amhr2	3	3	15.6	0.006062	0.038715	4.38	NOT	DAT	Receptor protein serine/threonine kinase GN=Amhr2
A2BH06	Rpl11	5	1	26.9	0.006508	0.040804	3.82	NOT	DAT	60S ribosomal protein L11 (Fragment) GN=Rpl11
Q3UV17	Krt76	2	2	15	0.006681	0.040875	2.39	DAT	NOT	Keratin_ type II cytoskeletal 2 oral GN=Krt76
Q810Y8	Pramel7	6	4	35	0.006682	0.040875	1.49	DAT	NOT	Preferentially expressed antigen in melanoma-like protein 7 GN=Pramel7
B9EIX2	Cep170b	4	2	26.8	0.006841	0.041044	2.85	NOT	DAT	AW555464 protein GN=Cep170b
Q3UZK5	Akap13	3	2	19.9	0.006861	0.041044	5.03	NOT	DAT	Uncharacterized protein (Fragment) GN=Akap13
E9QQ38	Ddhd1	3	3	20.4	0.007007	0.041537	2.11	NOT	DAT	Phospholipase DDHD1 GN=Ddhd1
Q0VAV0	Zfp947	2	2	15.9	0.007199	0.042289	2	DAT	NOT	Predicted gene_ EG210853 GN=Zfp947
A0A0R4J0F6	Gak	5	4	33.5	0.007796	0.044988	2.84	DAT	NOT	Cyclin-G-associated kinase GN=Gak
G3UZR1	Tubb5	4	1	41.2	0.008171	0.046738	3.85	NOT	DAT	Tubulin beta-5 chain (Fragment) GN=Tubb5
G3X9G2	Mink1	6	3	31.1	0.008453	0.04753	1.46	DAT	NOT	Misshapen-like kinase 1 GN=Mink1
P63038	Hspd1	48	43	479	0.008456	0.04753	1.76	DAT	NOT	60 kDa heat shock protein_ mitochondrial GN=Hspd1
F6Q404	Pdia3	6	1	34.6	0.00903	0.049811	1.79	NOT	DAT	Protein disulfide-isomerase A3 (Fragment) GN=Pdia3
Q9CVR0	Tubb4b	10	1	77.3	0.009108	0.049811	1.88	NOT	DAT	Tubulin beta chain (Fragment) GN=Tubb4b
Q69ZK1	N4bp2	5	3	22.5	0.009179	0.049811	1.67	DAT	NOT	MKIAA1413 protein (Fragment) GN=N4bp2
E9QA15	Cald1	8	6	41.7	0.009482	0.050679	2.12	DAT	NOT	Caldesmon 1 GN=Cald1
P99024	Tubb5	13	1	108	0.010344	0.053959	2.2	NOT	DAT	Tubulin beta-5 chain GN=Tubb5
F8VQ72	Map3k1	4	3	25.3	0.011311	0.058469	7.01	NOT	DAT	Mitogen-activated protein kinase kinase kinase 1 GN=Map3k1
Q922A0	Eno2	8	1	71.6	0.01148	0.058469	3.02	NOT	DAT	Eno2 protein (Fragment) GN=Eno2
Q3UDU4	Ldha	14	1	122	0.011567	0.058469	3.83	NOT	DAT	L-lactate dehydrogenase GN=Ldha
Q3TCI7	Ldha	15	4	126	0.011567	0.058469	3.83	NOT	DAT	L-lactate dehydrogenase GN=Ldha
A0A5F8MPR1	Epb41l3	4	2	17.1	0.01235	0.060812	1.99	NOT	DAT	Band 4.1-like protein 3 GN=Epb41l3
K3W4R2	Myh14	14	10	70.5	0.012789	0.062183	1.83	NOT	DAT	Myosin-14 GN=Myh14
A0A0R4J1M7	Scn5a	9	5	44.4	0.012895	0.062183	1.96	DAT	NOT	Sodium channel protein GN=Scn5a
E9QKC6	Trim2	3	1	22.3	0.013071	0.06221	2.55	DAT	NOT	Tripartite motif-containing protein 2 GN=Trim2
E9PWX1	Dpp6	4	2	18.6	0.014166	0.065976	12	NOT	DAT	Dipeptidyl aminopeptidase-like protein 6 GN=Dpp6
Q545V3	Eno2	12	4	88.8	0.015634	0.070505	2.45	NOT	DAT	Uncharacterized protein GN=Eno2
Q91Y50	Il4	3	1	27	0.015679	0.070505	2.02	NOT	DAT	Interleukin-4 GN=Il4
P05214	Tuba3a; Tuba3b	11	1	82.7	0.016349	0.072638	132	NOT	DAT	Tubulin alpha-3 chain GN=Tuba3a

B1ASE2	Atp5h	4	3	23.8	0.016376	0.072638	1.41	NOT	DAT	ATP synthase_ H+-transporting_ mitochondrial F0 complex_ subunit D (Fragment) GN=Atp5h
A0A1W2P6F6	Myl6	4	3	31.1	0.016804	0.073537	1.81	DAT	NOT	Myosin light polypeptide 6 GN=Myl6
B2RSU7	Gcc2	6	2	44.1	0.017369	0.075001	1.91	DAT	NOT	GRIP and coiled-coil domain containing 2 GN=Gcc2
S4R2M7	Pgk1	12	1	115	0.018003	0.076325	1.54	DAT	NOT	Phosphoglycerate kinase GN=Pgk1
O08553	Dpysl2	7	4	42	0.018016	0.076325	1.8	DAT	NOT	Dihydropyrimidinase-related protein 2 GN=Dpysl2
A0A2R8VHL8	Pcbp2	2	1	20	0.018043	0.076325	18.5	NOT	DAT	Poly(rC)-binding protein 2 (Fragment) GN=Pcbp2
Q504P4	Hspa8	24	1	203	0.01828	0.076325	1.79	DAT	NOT	Heat shock cognate 71 kDa protein GN=Hspa8
Q921L4	LOC665622	6	6	51.4	0.018405	0.076325	2.8	NOT	DAT	Histone H2B GN=LOC665622
B7ZCQ3	Hmgn2	3	3	33	0.018578	0.076325	2.54	NOT	DAT	Non-histone chromosomal protein HMG-17 GN=Hmgn2
P48678	Lmna	15	9	109	0.019392	0.078255	1.66	DAT	NOT	Prelamin-A/C GN=Lmna
A0A087WP85	Ktn1	5	3	30.2	0.019442	0.078255	2.01	DAT	NOT	Kinectin GN=Ktn1
Q6NZC6	Zim1	4	1	22.1	0.020482	0.08094	4.42	NOT	DAT	Zinc finger_ imprinted 1 GN=Zim1
Q8VCF1	Cant1	3	1	15.2	0.021307	0.083194	2.91	DAT	NOT	Soluble calcium-activated nucleotidase 1 GN=Cant1
Q6PDG0	Nup205	6	3	23.6	0.022523	0.086648	1.78	DAT	NOT	Nup205 protein (Fragment) GN=Nup205
E9PV14	Epb41l1	6	6	26.5	0.022649	0.086648	1.57	DAT	NOT	Band 4.1-like protein 1 GN=Epb41l1
Q3U3C9	Gse1	11	7	67.4	0.024252	0.090363	1.54	DAT	NOT	Genetic suppressor element 1 GN=Gse1
F8WIJ0	Slc12a4	5	3	38.9	0.024559	0.090509	2.88	DAT	NOT	Solute carrier family 12 member 4 GN=Slc12a4
Q6ZPW1	Zfp512b	4	4	18.5	0.026979	0.098277	1.38	DAT	NOT	MKIAA1196 protein (Fragment) GN=Zfp512b
S4R257	Gapdh	26	4	259	0.028422	0.102957	1.55	NOT	DAT	Glyceraldehyde-3-phosphate dehydrogenase (Fragment) GN=Gapdh
A0A5F8MPP4	Usp25	3	2	19.1	0.028778	0.103672	1.72	DAT	NOT	Ubiquitin carboxyl-terminal hydrolase 25 GN=Usp25
A0A0R4J0F3	Ppp1r32	3	1	28.3	0.029546	0.104702	2.91	NOT	DAT	Protein phosphatase 1 regulatory subunit 32 GN=Ppp1r32
D3Z2L1	Gtf3c2	3	1	16.7	0.032089	0.111294	2.15	NOT	DAT	General transcription factor 3C polypeptide 2 (Fragment) GN=Gtf3c2
B2RY59	Neurl4	5	3	32.3	0.032941	0.113049	1.8	NOT	DAT	Neurl4 protein GN=Neurl4
D3YYN7	Atp1a2	6	1	30.5	0.03366	0.114313	1.93	NOT	DAT	Sodium/potassium-transporting ATPase subunit alpha GN=Atp1a2
A2AFS0	Sars	4	3	24.1	0.033934	0.114646	1.81	DAT	NOT	Serine--tRNA ligase_ cytoplasmic (Fragment) GN=Sars
Q8C7V5	Foxm1	3	2	16.3	0.034456	0.115808	4.81	NOT	DAT	Fork-head domain-containing protein GN=Foxm1
Q3TXL0	Ero1a	4	4	22.8	0.035909	0.120075	3.4	NOT	DAT	Uncharacterized protein (Fragment) GN=Ero1l
A0A286YCM2	Hnrnpk	3	3	19.6	0.036144	0.120244	9.63	NOT	DAT	Heterogeneous nuclear ribonucleoprotein K (Fragment) GN=Hnrnpk
A0A3B2W7W2	Ppard	19	17	121	0.036966	0.120519	1.43	DAT	NOT	Peroxisome proliferator-activated receptor delta GN=Ppard
Q9R167	Zfp108	5	4	21.4	0.038144	0.12204	2.58	NOT	DAT	Zinc finger protein ZFP108 GN=Zfp108
Q3UQ07	Gm12359	2	1	15.3	0.03821	0.12204	1.77	DAT	NOT	Uncharacterized protein (Fragment) GN=Gm12359
Q3UGQ6	Zbtb43	4	2	23	0.038643	0.12204	2.82	DAT	NOT	Uncharacterized protein GN=Zbtb43
Q3TRH3	Hspa8	24	1	185	0.038649	0.12204	1.62	DAT	NOT	Uncharacterized protein GN=Hspa8
Q3T9F2	Gm10183	3	2	21.5	0.038743	0.12204	1.33	DAT	NOT	Uncharacterized protein GN=Gm10183
Q3UZG3	Hnrnpa3	12	11	87	0.039177	0.122813	1.71	DAT	NOT	Uncharacterized protein GN=Hnrnpa3
Q3UBP6	Actb	36	4	356	0.040245	0.124255	1.39	DAT	NOT	Uncharacterized protein GN=Actb
Q3U7D2	Rpl15	2	1	29	0.040268	0.124255	1.81	NOT	DAT	Ribosomal protein L15 GN=Rpl15
P62900	Rpl31	2	1	20.1	0.040399	0.124255	1.52	DAT	NOT	60S ribosomal protein L31 GN=Rpl31
Q5DTN5	Dhx8	4	2	20	0.041758	0.126428	2.4	DAT	NOT	MKIAA4096 protein (Fragment) GN=Dhx8
A0A1S6GWJ9	Tsfm	2	1	22.2	0.041783	0.126428	4.35	DAT	NOT	Elongation factor Ts_ mitochondrial GN=Tsfm
A0A0A6YXM1	Nphp3	6	2	27.1	0.041843	0.126428	1.72	DAT	NOT	Nephrocystin-3 (Fragment) GN=Nphp3
A0A087WRQ1	1700017N19Rik	5	3	23.4	0.044042	0.13234	2.12	DAT	NOT	RIKEN cDNA 1700017N19 gene GN=1700017N19Rik
A0A1Y7VLS3	Togaram1	5	3	29.9	0.045049	0.13386	2.03	NOT	DAT	TOG array regulator of axonemal microtubules protein 1 GN=Togaram1
P60335	Pcbp1	3	2	20.9	0.04645	0.136367	2.62	DAT	NOT	Poly(rC)-binding protein 1 GN=Pcbp1
Q8BG87	Tet3	4	4	20.1	0.046638	0.136367	1.91	DAT	NOT	Methylcytosine dioxygenase TET3 GN=Tet3
Q8QZY6	Tspan14	6	4	36	0.047878	0.139181	1.75	DAT	NOT	Tetraspanin-14 GN=Tspan14

Table S3: Properties of the identified proteins following direct antibody treatment (DAT) of the mouse primary neuron (MPN) in comparison with 3F4 antibody treated cells. The properties were identified by Progenesis Software after the LC-MS analysis.

Accession	Gene ID	Peptides	Unique peptides	Confidence score	Anova (p)	q Value	Max fold change	Highest Mean	Lowest Mean	Description
O70291	Grk4	3	1	19.1	2.08E-06	0.000538	1.07 E+03	3F4	DAT	G protein-coupled receptor kinase 4 GN=Grk4
A0A2R8VH H1	Nup155	5	2	31.3	6.29E-06	0.000978	69.3	3F4	DAT	Nuclear pore complex protein Nup155 GN=Nup155
B2RRA8	Zfp114	3	3	18	1.07E-05	0.001245	7.32	3F4	DAT	Zfp114 protein GN=Zfp114
Q3TCI7	Ldha	15	4	126	1.31E-05	0.001245	14.9	3F4	DAT	L-lactate dehydrogenase GN=Ldha
Q3UDU4	Ldha	14	1	122	1.31E-05	0.001245	14.9	3F4	DAT	L-lactate dehydrogenase GN=Ldha
Q8BI84	Mia3	2	1	16.4	1.44E-05	0.001245	19.5	3F4	DAT	Transport and Golgi organization protein 1 homolog GN=Mia3
A0A571BEW 5	Lpar2	3	2	19.3	1.65E-05	0.001283	32.4	DAT	3F4	Lysophosphatidic acid receptor 2 GN=Lpar2
D3YVU4	Kdm2b	3	2	17.1	2.29E-05	0.001619	4.98	3F4	DAT	Lysine-specific demethylase 2B GN=Kdm2b
Q66JU1	Eif3c	2	1	16.5	2.65E-05	0.001716	14.8	3F4	DAT	Eif3c protein (Fragment) GN=Eif3c
A2A931	Prdm16	4	4	20.3	4.17E-05	0.002492	44.5	3F4	DAT	Histone-lysine N-methyltransferase PRDM16 GN=Prdm16
O70475	Ugdh	4	3	19.7	0.000137	0.005909	6.12	3F4	DAT	UDP-glucose 6-dehydrogenase GN=Ugdh
A2AEP2	Ccnb3	6	6	35	0.00015	0.006139	2.42	DAT	3F4	Cyclin B3 GN=Ccnb3
Q9JL04	Fmn2	3	2	29.6	0.000248	0.008766	4.29	3F4	DAT	Formin-2 GN=Fmn2
P80316	Cct5	8	4	49.9	0.000393	0.012708	7.08	3F4	DAT	T-complex protein 1 subunit epsilon GN=Cct5
B2RY59	Neurl4	5	3	32.3	0.000431	0.013384	2.26	3F4	DAT	Neurl4 protein GN=Neurl4
E7CHD7	Rpgrip1	3	2	17.5	0.000517	0.014884	85.3	3F4	DAT	RPGRIP1 variant-11aa GN=Rpgrip1
Q58E64	Eef1a1	22	2	253	0.000541	0.014884	9.08	3F4	DAT	Elongation factor 1-alpha GN=Eef1a1
A0A0R4J0A4	Flt1	4	4	15.6	0.000542	0.014884	5.12	3F4	DAT	Vascular endothelial growth factor receptor 1 GN=Flt1
Q5I2A0	Serpina3g	5	5	21.9	0.000556	0.014884	10.9	DAT	3F4	Serine protease inhibitor A3G GN=Serpina3g
Q3TYF2	Pacsin1	4	2	26.8	0.000728	0.018256	2.4	3F4	DAT	Uncharacterized protein GN=Pacsin1
P40142	Tkt	24	6	199	0.001049	0.024705	2.06	DAT	3F4	Transketolase GN=Tkt
Q91YN0	D6Wsu163 e	3	2	21.4	0.001087	0.024837	3.1	3F4	DAT	Protein C12orf4 homolog GN=D6Wsu163e
A2A841	Epb41	5	4	35.5	0.001155	0.025531	2.42	DAT	3F4	Protein 4.1 GN=Epb41
Q6P1E8	Efcab6	7	5	41.6	0.001826	0.035477	1.38	3F4	DAT	EF-hand calcium-binding domain-containing protein 6 GN=Efcab6
B9EIX2	Cep170b	4	2	26.8	0.002388	0.045254	3.35	3F4	DAT	AW555464 protein GN=Cep170b
P48678	Lmna	15	9	109	0.003897	0.068809	1.59	3F4	DAT	Prelamin-A/C GN=Lmna
A2AUM9	Cep152	8	4	43.4	0.004152	0.0717	2.03	3F4	DAT	Centrosomal protein of 152 kDa GN=Cep152
P56480	Atp5f1b	26	19	213	0.004416	0.074595	2.33	3F4	DAT	ATP synthase subunit beta_ mitochondrial GN=Atp5f1b
E9Q039	Npepps	4	3	24	0.006189	0.100177	2.87	DAT	3F4	Aminopeptidase GN=Npepps
Q8VHU9		4	1	28.4	0.006467	0.100496	12.3	3F4	DAT	Eukaryotic translation initiation factor 5A (Fragment)
Q99KI0	Aco2	13	11	74	0.007022	0.102946	1.28	3F4	DAT	Aconitate hydratase_ mitochondrial GN=Aco2
P03995	Gfap	6	4	44.9	0.007408	0.104651	2.09	3F4	DAT	Glial fibrillary acidic protein GN=Gfap
A0A0U1RN K7	Dock7	8	4	58.2	0.008247	0.112418	3.67	3F4	DAT	Dedicator of cytokinesis protein 7 GN=Dock7
Q8BNV9	Hoxc8	2	2	15.7	0.00874	0.114883	2.61	3F4	DAT	Homeobox domain-containing protein (Fragment) GN=Hoxc8
Q3U741	Ddx17	6	4	33.9	0.009554	0.116711	1.28	DAT	3F4	Probable ATP-dependent RNA helicase DDX17 GN=Ddx17
P15864	H1-2	8	1	98	0.009624	0.116711	1.92	3F4	DAT	Histone H1.2 GN=H1-2
G3UW90	Trps1	2	1	20.5	0.009914	0.116711	2.37	3F4	DAT	Zinc finger transcription factor Trps1 GN=Trps1
G5E898	Ppl	3	1	15.6	0.010981	0.127351	1.78	3F4	DAT	Periplakin GN=Ppl
E9Q0H6	Fabp7	2	2	16	0.012505	0.13832	2.08	3F4	DAT	Fatty acid-binding protein_ brain GN=Fabp7

A0A0R4J0F3	Ppp1r32	3	1	28.3	0.012639	0.13832	3.77	3F4	DAT	Protein phosphatase 1 regulatory subunit 32 GN=Ppp1r32
Q3UYB8	Bfsp2	2	2	16.4	0.012875	0.138939	2.5	3F4	DAT	IF rod domain-containing protein GN=Bfsp2
Q3TXL0	Ero1a	4	4	22.8	0.013641	0.145188	2.54	3F4	DAT	Uncharacterized protein (Fragment) GN=Ero1l
Q3TRH3	Hspa8	24	1	185	0.014146	0.148432	1.7	3F4	DAT	Uncharacterized protein GN=Hspa8
B2RY51	Trip11	14	9	76	0.014327	0.148432	1.47	3F4	DAT	Thyroid hormone receptor interactor 11 GN=Trip11
A2RTV8	Rln1	3	2	21.6	0.014583	0.149097	1.87	3F4	DAT	Prorelaxin 1 GN=Rln1
Q504P4	Hspa8	24	1	203	0.014974	0.150078	1.7	3F4	DAT	Heat shock cognate 71 kDa protein GN=Hspa8
A0A5F8MPM7		3	2	15.4	0.015727	0.154081	2.58	3F4	DAT	Cingulin GN=Cgn
A0A0A0MQE8	Arhgap21	4	3	14.9	0.015864	0.154081	1.62	3F4	DAT	Rho GTPase-activating protein 21 GN=Arhgap21
Q3TH56	Hspa8	27	2	212	0.018351	0.169838	1.37	3F4	DAT	Uncharacterized protein GN=Hspa8
Q8BI87	Zfp536	5	2	29.1	0.019704	0.178019	2.14	DAT	3F4	Uncharacterized protein GN=Zfp536
Q9QZ83	Actg1	25	1	267	0.020739	0.185218	2.44	DAT	3F4	Gamma actin-like protein GN=Actg1
D3Z6I8	Tpm3	4	4	43.6	0.022731	0.193879	1.78	3F4	DAT	Tropomyosin alpha-3 chain GN=Tpm3
S4R1I4	Cep112	6	4	34.7	0.024967	0.206379	2.09	DAT	3F4	Centrosomal protein of 112 kDa GN=Cep112
Q7M6X7	Zfp457	3	2	16.4	0.02509	0.206379	1.89	DAT	3F4	Zinc finger protein 457 GN=Zfp457
Q8VDD5	Myh9	16	10	94.9	0.025409	0.206379	1.47	3F4	DAT	Myosin-9 GN=Myh9
E9Q9Y2	Ncor1	5	3	23.7	0.025812	0.206765	1.59	3F4	DAT	Nuclear receptor corepressor 1 GN=Ncor1
A0A0R4J1L9	Mtus1	3	1	15.8	0.029234	0.231786	1.42	3F4	DAT	Microtubule-associated tumor suppressor 1 homolog GN=Mtus1
Q8VC94	Rpl11	4	1	24	0.030881	0.241476	1.95	3F4	DAT	Rpl11 protein GN=Rpl11
Q80U88	mKIAA0038	2	1	15.2	0.031268	0.241476	2.13	3F4	DAT	MKIAA0038 protein (Fragment) GN=mKIAA0038
A0A1W2P6F6	Myl6	4	3	31.1	0.031389	0.241476	1.67	DAT	3F4	Myosin light polypeptide 6 GN=Myl6
Q8CF60	Znf263	9	8	61.5	0.031949	0.242375	1.55	DAT	3F4	Zinc finger protein 263 GN=Zfp263
Q8BMK4	Ckap4	3	2	20	0.032518	0.242945	1.76	DAT	3F4	Cytoskeleton-associated protein 4 GN=Ckap4
A0A0R4J0F6	Gak	5	4	33.5	0.03711	0.267908	2.67	DAT	3F4	Cyclin-G-associated kinase GN=Gak
B2RSN3	Tubb2b	17	4	129	0.037686	0.267908	1.15	3F4	DAT	Tubulin beta chain GN=Tubb2b
E9Q3V9	Adgrl1	6	6	26.3	0.037928	0.267908	1.33	DAT	3F4	Adhesion G protein-coupled receptor L1 GN=Adgrl1
Q6NZC6	Zim1	4	1	22.1	0.039303	0.272664	3.26	3F4	DAT	Zinc finger_ imprinted 1 GN=Zim1
A0A0R4J1E2	Eef1d	4	2	27.4	0.041579	0.283392	1.67	3F4	DAT	Elongation factor 1-delta GN=Eef1d
Q9WUF3	Casp8ap2	16	10	98.3	0.042856	0.286423	2.09	DAT	3F4	CASP8-associated protein 2 GN=Casp8ap2
B1ASE2	Atp5h	4	3	23.8	0.044441	0.292633	1.24	3F4	DAT	ATP synthase_ H+-transporting_ mitochondrial F0 complex_ subunit D (Fragment) GN=Atp5h
C3S7Q5	Ofcc1	3	2	17.9	0.047028	0.302589	2.15	3F4	DAT	Ojoplano variant A GN=Ofcc1
A0A5F8MPH5	Sytl2	6	4	28.2	0.048267	0.304908	1.65	3F4	DAT	Synaptotagmin-like protein 2 GN=Sytl2
Q9CYV4	Zfp329	3	3	18.2	0.049219	0.306061	1.29	3F4	DAT	Uncharacterized protein GN=Zfp329
Q8BK67	Rcc2	5	3	28.4	0.049238	0.306061	1.81	3F4	DAT	Protein RCC2 GN=Rcc2

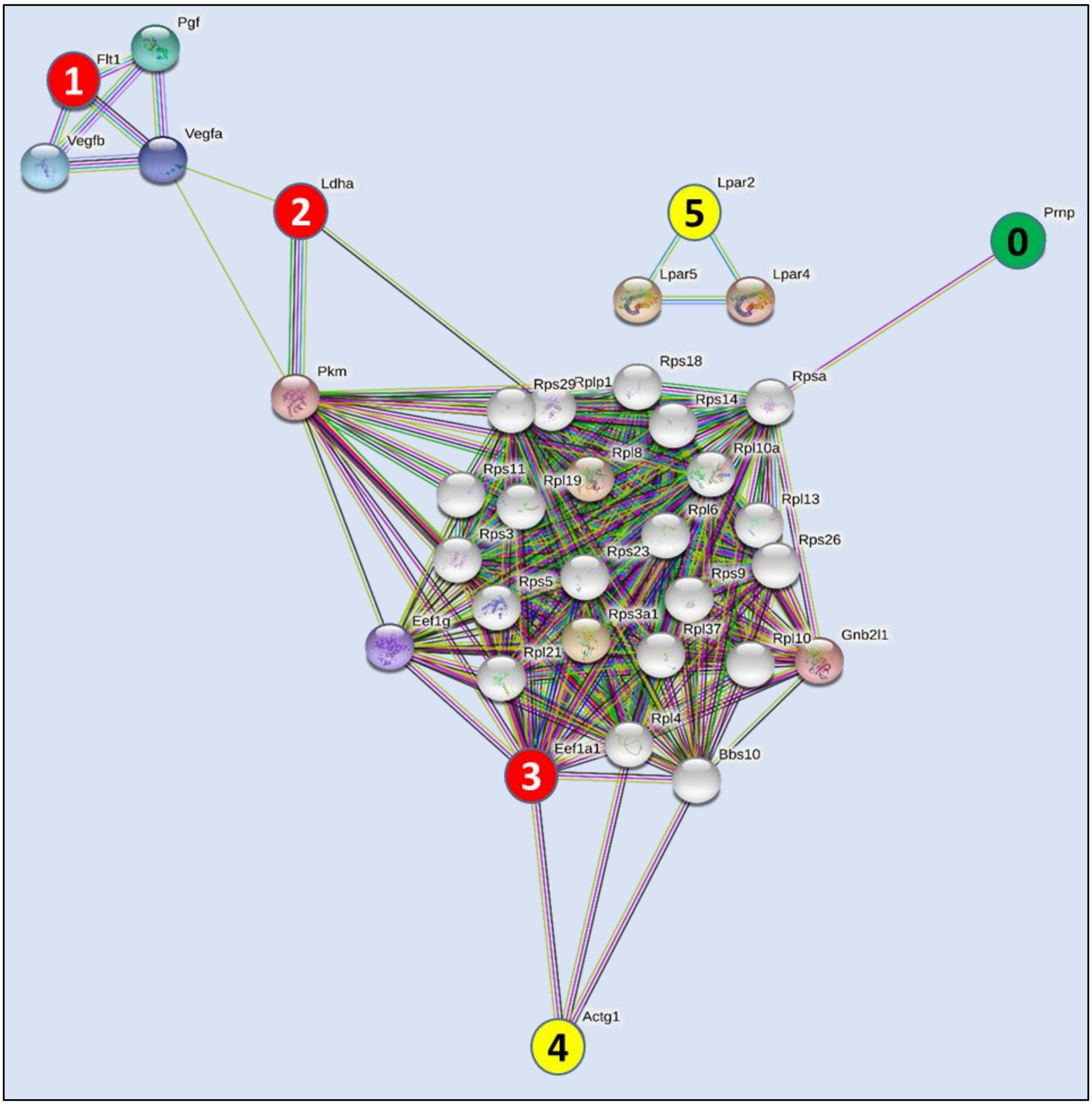

Figure S2: Protein-protein interaction of the identified 5 allergenic genes following direct antibody treatment (DAT) from mouse primary neuron (MPN) in comparison with 3F4 antibody treated cells. Protein-protein interaction analysis was performed using STRING v11.0 server. The *Mus Musculus* database was used as the host organism for the identification of protein interactions. Interaction among the allergenic proteins (numbered 1, 2, 3, 4 & 5) and with PrPC (numbered 0) following direct antibody treatment, where red and yellow color indicate the downregulated and upregulated genes, respectively and green color represents the cellular prion protein PrPC.

Table S4: Properties of the identified proteins following direct microglia treatment (DMT). The properties were identified by Progenesis Software after the LC-MS analysis. Selected 113 proteins have been highlighted as bold.

Accession	Peptides	Unique peptides	Confidence score	Anova (p)	q Value	Max fold change	Highest Mean	Lowest Mean	Description
**Q62108**	**7**	**2**	**42.9**	**6.61E-11**	**6.85E-10**	**α**	**DMT**	**CTL**	**Disks large homolog 4 GN=Dlg4**
Q921I1	4	2	28.8	0.081746	0.044888	α	DMT	CTL	Serotransferrin GN=Tf
Q3UFZ6	6	2	36.3	0	0	α	DMT	CTL	Caprin-1_dimer domain-containing protein (Fragment) GN=Caprin1
Q0VGM9	7	3	37	2.22E-16	1.16E-14	α	DMT	CTL	Regulator of telomere elongation helicase 1 GN=Rtel1
J3QQ40	2	2	10.6	0.225732	0.104893	α	DMT	CTL	Torsin-1A-interacting protein 1 GN=Tor1aip1
D3Z601	6	2	34.4	0.596358	0.223713	α	DMT	CTL	Calsyntenin-3 GN=Clstn3
B7ZNM3	5	3	31.3	1.14E-06	2.94E-06	α	DMT	CTL	NAD(P)(+)--arginine ADP-ribosyltransferase GN=Art3
A0A140LIX0	4	2	20	3.71E-08	1.49E-07	α	DMT	CTL	A-kinase anchor protein 13 (Fragment) GN=Akap13
A0A087WP13	5	2	24.9	0	0	α	DMT	CTL	Pleckstrin homology domain-containing family A member 6 (Fragment) GN=Plekha6
F8VQF9	3	2	13.4	8.88E-16	4.45E-14	5510	DMT	CTL	Trafficking protein particle complex subunit 10 GN=Trappc10
**A0A1L1SSA4**	**20**	**8**	**100**	**3.06E-08**	**1.26E-07**	**3580**	**CTL**	**DMT**	**Centrosomal protein of 164 kDa GN=Cep164**
Q8C5G4	2	2	11.1	1.66E-10	1.46E-09	1400	DMT	CTL	Uncharacterized protein GN=4930451G09Rik
**A9C491**	**12**	**4**	**56.7**	**5E-08**	**1.92E-07**	**1210**	**CTL**	**DMT**	**Cullin 7 GN=Cul7**
**A2AST1**	**17**	**6**	**110**	**6.66E-15**	**2.55E-13**	**1180**	**DMT**	**CTL**	**Coiled-coil domain-containing 141 GN=Ccdc141**
Q8BLR6	2	2	10.4	3.08E-10	2.54E-09	530	DMT	CTL	Uncharacterized protein (Fragment)
**A2RTI3**	**8**	**2**	**51.3**	**3.27E-07**	**1.01E-06**	**443**	**DMT**	**CTL**	**Legumain GN=Lgmn**
Q99JF8	5	2	30.7	2.47E-12	4.05E-11	353	DMT	CTL	PC4 and SFRS1-interacting protein GN=Psip1
Q3UPH7	7	2	36.2	1.36E-11	1.81E-10	292	DMT	CTL	Rho guanine nucleotide exchange factor 40 GN=Arhgef40
Q6ZPK7	5	2	26.8	1E-08	4.92E-08	275	DMT	CTL	Lateral signaling target protein 2 homolog GN=Zfyve28
**Q9QXL1**	**36**	**2**	**200**	**1.48E-09**	**9.64E-09**	**252**	**DMT**	**CTL**	**Kinesin-like protein KIF21B GN=Kif21b**
**F8WHT3**	**16**	**3**	**96**	**5.32E-10**	**3.88E-09**	**249**	**DMT**	**CTL**	**Protein PRRC2B GN=Prrc2b**
**Q9CVR0**	**32**	**2**	**386**	**1.68E-06**	**4.1E-06**	**237**	**CTL**	**DMT**	**Tubulin beta chain (Fragment) GN=Tubb4b**
**Q3TWV0**	**61**	**2**	**865**	**0.000478**	**0.000553**	**221**	**DMT**	**CTL**	**IF rod domain-containing protein GN=Vim**
**P62717**	**14**	**3**	**93.6**	**1.86E-12**	**3.24E-11**	**206**	**DMT**	**CTL**	**60S ribosomal protein L18a GN=Rpl18a**
**Q8CHC7**	**9**	**2**	**54.1**	**2.13E-09**	**1.31E-08**	**200**	**DMT**	**CTL**	**MKIAA0864 protein (Fragment) GN=Mprip**
**E9PZM4**	**9**	**2**	**46.6**	**2.93E-09**	**1.69E-08**	**189**	**DMT**	**CTL**	**Chromodomain-helicase-DNA-binding protein 2 GN=Chd2**
E9Q5P0	3	2	16.9	0.00079	0.000852	158	DMT	CTL	Serine protease hepsin GN=Hpn
A1L2Z3	5	2	29.3	0.000681	0.000751	153	DMT	CTL	C230096C10Rik protein GN=Emc1
O54775	2	2	16.6	7.78E-11	7.86E-10	152	DMT	CTL	WNT1-inducible-signaling pathway protein 1 GN=Wisp1
B7ZNG2	3	3	14.7	1.81E-08	8.16E-08	135	DMT	CTL	Ttc18 protein GN=Cfap70
B1AVK5	5	3	34.2	1.6E-06	3.95E-06	131	DMT	CTL	Collagen_ type IV_ alpha 6 GN=Col4a6
**Q91WD7**	**11**	**2**	**65.9**	**3.8E-05**	**6.26E-05**	**124**	**DMT**	**CTL**	**Kinesin-like protein KIF18A GN=Kif18a**
**D3YTQ3**	**13**	**2**	**131**	**6E-15**	**2.37E-13**	**117**	**DMT**	**CTL**	**Heterogeneous nuclear ribonucleoprotein D-like GN=Hnrnpdl**
**Q8VDC1**	**10**	**2**	**64.5**	**9.25E-07**	**2.5E-06**	**115**	**CTL**	**DMT**	**FYVE and coiled-coil domain-containing protein 1 GN=Fyco1**
E5RKG4	6	3	34.2	0.011904	0.008784	109	DMT	CTL	Scavenger receptor FEEL-2e GN=Stab2
E9JGN0	4	2	21.6	0.000109	0.000154	105	DMT	CTL	DAP-kinase-related protein 1 beta isoform GN=Dapk2
Q3TEA8	6	2	32.3	4.78E-10	3.58E-09	97.8	DMT	CTL	Heterochromatin protein 1-binding protein 3 GN=Hp1bp3
A0A097BW18	3	2	16.3	1.19E-05	2.28E-05	90.6	DMT	CTL	Periostin isoform M1 GN=Postn
Q3ZAT5	3	2	15.8	4.1E-11	4.55E-10	83.4	DMT	CTL	One cut domain family member GN=Onecut2
**Q3UKW2**	**12**	**2**	**107**	**2.01E-06**	**4.85E-06**	**80.5**	**DMT**	**CTL**	**Calmodulin-1 GN=Calm1**
Q0PD40	4	2	27	2.9E-10	2.42E-09	72.8	DMT	CTL	Rab15 GN=Rab15
**A0A1L1SSN6**	**33**	**5**	**363**	**4.84E-12**	**7.17E-11**	**71.5**	**CTL**	**DMT**	**Pyruvate kinase PKM (Fragment) GN=Pkm**
**P41230**	**8**	**2**	**42.7**	**5.23E-10**	**3.83E-09**	**66.5**	**DMT**	**CTL**	**Lysine-specific demethylase 5C GN=Kdm5c**
**A0A1S6GWJ7**	**26**	**10**	**157**	**4.85E-11**	**5.31E-10**	**66**	**DMT**	**CTL**	**Uncharacterized protein GN=Pi4ka**
P45952	4	2	22.1	2.22E-16	1.16E-14	63.1	DMT	CTL	Medium-chain specific acyl-CoA dehydrogenase_ mitochondrial GN=Acadm
Q91YL3	3	2	15.8	1.1E-14	3.98E-13	60.4	DMT	CTL	Uridine-cytidine kinase-like 1 GN=Uckl1

Q9CUU3	3	2	15.8	3.99E-09	2.16E-08	58.7	DMT	CTL	Synaptonemal complex protein 2 GN=Sycp2
Q31129	3	2	21.4	8.39E-05	0.000123	56.4	CTL	DMT	MHC class I cell surface glycoprotein GN=H2-T24
A6H619	7	2	37.1	0.000541	0.000618	55.1	DMT	CTL	PHD and RING finger domain-containing protein 1 GN=Phrf1
**Q9ES28**	**10**	**2**	**63.1**	**6.76E-07**	**1.87E-06**	**50.8**	**DMT**	**CTL**	**Rho guanine nucleotide exchange factor 7 GN=Arhgef7**
Q8C6E1	4	2	21.3	2.22E-16	1.16E-14	49.6	DMT	CTL	Apolipoprotein L 7c GN=Apol7c
**Q8R1B4**	**15**	**2**	**95**	**0.000999**	**0.00103**	**48.3**	**CTL**	**DMT**	**Eukaryotic translation initiation factor 3 subunit C GN=Eif3c**
Q6XBG3	6	3	29	3.92E-07	1.18E-06	47	CTL	DMT	ATP-binding cassette transporter sub-family A member 14 GN=Abca14
**A2AFF6**	**12**	**3**	**65.2**	**1.15E-09**	**7.8E-09**	**46.2**	**DMT**	**CTL**	**Cohesin subunit SA-2 GN=Stag2**
A0A2R8VJU7	4	2	27.8	9.93E-05	0.000142	45.9	DMT	CTL	Septin 12 GN=Sept12
Q8C4B1	2	2	11.1	1.98E-08	8.86E-08	44.7	DMT	CTL	Uncharacterized protein GN=Larp1b
**A0A0N4SUT9**	**11**	**3**	**62.3**	**3.8E-10**	**2.98E-09**	**44.7**	**DMT**	**CTL**	**Peregrin GN=Brpf1**
F6UAC8	2	2	9.4	0.001663	0.001613	44.4	CTL	DMT	Prolow-density lipoprotein receptor-related protein 1 (Fragment) GN=Lrp1
**P03995**	**20**	**2**	**153**	**2.35E-09**	**1.41E-08**	**44.3**	**DMT**	**CTL**	**Glial fibrillary acidic protein GN=Gfap**
**S4R2E2**	**9**	**3**	**60.1**	**3.37E-10**	**2.73E-09**	**42.3**	**DMT**	**CTL**	**Protein PRRC2C (Fragment) GN=Prrc2c**
**A0A2I3BQG2**	**14**	**2**	**71.4**	**8E-05**	**0.000119**	**41.5**	**DMT**	**CTL**	**Crystallin beta-gamma domain-containing 2 GN=Crybg2**
**K3W4P2**	**43**	**8**	**228**	**9.03E-09**	**4.55E-08**	**41.3**	**DMT**	**CTL**	**Integrator complex subunit 1 GN=Ints1**
**Q3T9T5**	**8**	**2**	**51.3**	**0.02772**	**0.017929**	**41**	**DMT**	**CTL**	**Uncharacterized protein (Fragment) GN=Cd22**
**D3YWM8**	**10**	**3**	**56.5**	**4.86E-07**	**1.41E-06**	**40.8**	**DMT**	**CTL**	**Semaphorin-6A GN=Sema6a**
Q32NZ6	2	2	11	2.49E-13	6.13E-12	40.2	CTL	DMT	Transmembrane channel-like protein 5 GN=Tmc5
Q3U0X6	4	2	20.3	0.000921	0.000968	39.5	DMT	CTL	Zinc finger protein 217 GN=Zfp217
**P14152**	**28**	**2**	**248**	**9.76E-10**	**6.73E-09**	**38.2**	**DMT**	**CTL**	**Malate dehydrogenase_ cytoplasmic GN=Mdh1**
**A0A498WGD8**	**11**	**3**	**92.4**	**1.15E-07**	**3.98E-07**	**37.9**	**CTL**	**DMT**	**Thioredoxin-like protein 1 GN=Txnl1**
**Q3TLE5**	**33**	**3**	**255**	**1.01E-13**	**2.69E-12**	**36.6**	**DMT**	**CTL**	**S5 DRBM domain-containing protein GN=Rps2**
F6Z6C6	3	2	16.3	3.15E-07	9.74E-07	36.1	CTL	DMT	Interferon regulatory factor 5 GN=Irf5
Q6P5U7	6	3	32.2	0.002113	0.001984	35.9	DMT	CTL	NACHT and WD repeat domain-containing protein 2 GN=Nwd2
Q8C4X2	3	2	16.6	2.36E-10	2.01E-09	35.7	DMT	CTL	Casein kinase I isoform gamma-3 GN=Csnk1g3
Q9Z2F2	4	2	22.8	2.19E-13	5.49E-12	35.2	DMT	CTL	2'-5'-oligoadenylate synthase-like protein 2 GN=Oasl2
A0A0E4HJ81	3	2	21.5	0.000272	0.000339	35.1	DMT	CTL	Katanin p60 ATPase-containing subunit A-like 2 GN=Katnal2
**Q3V2C6**	**18**	**3**	**173**	**1.11E-08**	**5.32E-08**	**34.5**	**DMT**	**CTL**	**IF rod domain-containing protein GN=Des**
B2RUH4	4	2	26.7	0.000361	0.000432	34.5	DMT	CTL	Pcdhb5 protein GN=Pcdhb5
G3UW86	5	2	26.7	7.14E-08	2.68E-07	34.4	DMT	CTL	Protein regulator of cytokinesis 1 GN=Prc1
**A2RSQ0**	**11**	**4**	**57.1**	**5.61E-10**	**4.04E-09**	**33.9**	**CTL**	**DMT**	**DENN domain-containing protein 5B GN=Dennd5b**
**Q3U094**	**16**	**2**	**133**	**1.32E-06**	**3.32E-06**	**32.7**	**DMT**	**CTL**	**Actin-related protein 2/3 complex subunit GN=Arpc1b**
**Q62240**	**11**	**3**	**63.9**	**8.71E-08**	**3.18E-07**	**31.6**	**DMT**	**CTL**	**Lysine-specific demethylase 5D GN=Kdm5d**
Q6ZQA5	3	2	15.6	2.48E-08	1.06E-07	31.5	DMT	CTL	MKIAA0467 protein (Fragment) GN=mKIAA0467
**A0A286YD12**	**5**	**2**	**40.5**	**0.019522**	**0.013368**	**31.4**	**DMT**	**CTL**	**Neurolysin_ mitochondrial GN=Nln**
**Q8C555**	**14**	**2**	**77.5**	**1.74E-09**	**1.08E-08**	**31.2**	**DMT**	**CTL**	**Uncharacterized protein**
E9Q7D5	6	3	31.3	7.61E-10	5.3E-09	30.8	DMT	CTL	Rho guanine nucleotide exchange factor 5 GN=Arhgef5
A2BI45	3	2	23	0.010774	0.00806	30.6	DMT	CTL	X-linked lymphocyte-regulated 5A GN=Xlr5a
**E9Q0J5**	**9**	**2**	**50.7**	**0.013141**	**0.009519**	**30.1**	**CTL**	**DMT**	**Kinesin-like protein KIF21A GN=Kif21a**
Q148T5	5	2	26.6	0.005851	0.004786	30	CTL	DMT	Tnc protein GN=Tnc
**A0A1W2P711**	**8**	**2**	**45.6**	**5.95E-05**	**9.32E-05**	**29.2**	**DMT**	**CTL**	**Melanoma inhibitory activity protein 2 GN=Mia2**
**D3Z1N4**	**21**	**9**	**112**	**3.83E-07**	**1.16E-06**	**28.3**	**CTL**	**DMT**	**Protein polybromo-1 GN=Pbrm1**
**O89054**	**16**	**2**	**201**	**2.63E-05**	**4.58E-05**	**28.2**	**DMT**	**CTL**	**Cytoskeletal beta-actin (Fragment) GN=Actb**
Q0PD15	5	3	30.4	0.005907	0.004825	27.9	CTL	DMT	Rab39A GN=Rab39
E9QAC9	4	2	17.7	1.31E-05	2.47E-05	27.8	DMT	CTL	Calcium-independent phospholipase A2-gamma GN=Pnpla8
A0A0R4J1H1	4	4	20	0.122918	0.063149	27.1	DMT	CTL	RAD51-associated protein 1 GN=Rad51ap1
G3UWG2	4	2	21.3	1.49E-07	5.05E-07	27.1	DMT	CTL	T-lymphoma invasion and metastasis-inducing protein 1 GN=Tiam1
**A0A0A0MQE8**	**21**	**7**	**122**	**2.77E-07**	**8.78E-07**	**26.6**	**DMT**	**CTL**	**Rho GTPase-activating protein 21 GN=Arhgap21**
**J9S314**	**16**	**5**	**85.7**	**2.63E-09**	**1.54E-08**	**25.7**	**CTL**	**DMT**	**Transient receptor potential cation channel_ subfamily M_ member 3 GN=Trpm3**
E9Q6E5	5	2	32.5	9.45E-07	2.54E-06	25.6	DMT	CTL	Serine and arginine-rich-splicing factor 11 GN=Srsf11
Q3KN99	5	2	34.6	2.64E-09	1.55E-08	25.1	CTL	DMT	Synapsin III GN=Syn3

**A0A087WPL5**	**36**	**11**	**226**	**0**	**0**	**25**	**CTL**	**DMT**	**ATP-dependent RNA helicase A GN=Dhx9**
**Q61481**	**10**	**2**	**60.6**	**4.35E-14**	**1.26E-12**	**24.7**	**CTL**	**DMT**	**Calcium/calmodulin-dependent 3'_5'-cyclic nucleotide phosphodiesterase 1A GN=Pde1a**
**B2RSV4**	**11**	**2**	**57.4**	**3.62E-10**	**2.86E-09**	**24.1**	**DMT**	**CTL**	**Splicing factor 3b_ subunit 3 GN=Sf3b3**
**E9QK89**	**10**	**3**	**52.9**	**1.07E-12**	**2.18E-11**	**23.9**	**DMT**	**CTL**	**Mediator of DNA damage checkpoint protein 1 GN=Mdc1**
P30415	3	2	16.9	0.803512	0.278568	23.6	DMT	CTL	NK-tumor recognition protein GN=Nktr
Q8K485	2	2	16.8	2.54E-07	8.13E-07	23.3	DMT	CTL	BTB/POZ domain-containing protein KCTD11 GN=Kctd11
**A2AL50**	**9**	**2**	**59.6**	**0.001093**	**0.001114**	**23**	**DMT**	**CTL**	**Alkylglycerone-phosphate synthase GN=Agps**
**O35129**	**18**	**3**	**160**	**1.45E-09**	**9.48E-09**	**22.8**	**CTL**	**DMT**	**Prohibitin-2 GN=Phb2**
Q8C0V9	4	3	22.6	0.005808	0.004757	22.6	DMT	CTL	FERM domain-containing protein 6 GN=Frmd6
Q80TB9	2	2	10.9	4.26E-06	9.29E-06	22.4	DMT	CTL	MKIAA1569 protein (Fragment) GN=mKIAA1569
H7BX69	4	2	23.1	0.004424	0.00375	22.4	CTL	DMT	Zinc finger and BTB domain-containing protein 18 GN=Zbtb18
A2A9L3	2	2	10	2.02E-06	4.85E-06	22.2	DMT	CTL	Serine/threonine-protein kinase PDIK1L GN=Pdik1l
**O08605**	**7**	**3**	**61.6**	**2.22E-05**	**3.93E-05**	**21.5**	**DMT**	**CTL**	**MAP kinase-interacting serine/threonine-protein kinase 1 GN=Mknk1**
**G3X932**	**7**	**4**	**45.6**	**0.000202**	**0.000261**	**21.3**	**DMT**	**CTL**	**Rho GTPase-activating protein 39 GN=Arhgap39**
Q9Z2Q6	7	2	36.7	2.04E-08	9E-08	21	CTL	DMT	Septin-5 GN=Septin5
B2RXY1	5	2	36.7	1.74E-08	7.86E-08	21	DMT	CTL	Spock1 protein GN=Spock1
**A2AP18**	**17**	**6**	**99.8**	**0.00152**	**0.001494**	**20.9**	**DMT**	**CTL**	**1-phosphatidylinositol 4_5-bisphosphate phosphodiesterase eta-2 GN=Plch2**
**A0A0N4SVP8**	**25**	**2**	**214**	**6.74E-05**	**0.000103**	**20.4**	**DMT**	**CTL**	**Eukaryotic translation initiation factor 4A3-like 2 GN=Eif4a3l2**
**P70691**	**9**	**3**	**60.6**	**5.71E-11**	**6.06E-10**	**20.3**	**DMT**	**CTL**	**UDP-glucuronosyltransferase 1-2 GN=Ugt1a2**
**F8VPQ4**	**8**	**3**	**49.6**	**0.00027**	**0.000337**	**19.8**	**DMT**	**CTL**	**SLIT-ROBO Rho GTPase-activating protein 3 GN=Srgap3**
A0A338P6D4	3	2	16.5	1.25E-06	3.18E-06	19.6	DMT	CTL	Protein HEG homolog 1 GN=Heg1
B7ZBV8	3	2	16.6	1.91E-06	4.64E-06	19.6	DMT	CTL	Potassium voltage-gated channel subfamily KQT member 2 GN=Kcnq2
**Q8K441**	**7**	**5**	**41.9**	**0.001531**	**0.001503**	**19.3**	**DMT**	**CTL**	**ATP-binding cassette sub-family A member 6 GN=Abca6**
**Q99JW2**	**10**	**2**	**62.7**	**0.015458**	**0.010943**	**18.9**	**CTL**	**DMT**	**Aminoacylase-1 GN=Acy1**
**A0A571BEC4**	**14**	**3**	**96.5**	**2.95E-05**	**5.05E-05**	**18.8**	**DMT**	**CTL**	**Ligand-dependent nuclear receptor corepressor-like protein GN=Lcorl**
B1AZQ9	5	2	26.6	0.000653	0.000729	18.5	DMT	CTL	Kelch-like 4 GN=Klhl4
F6QKE4	6	3	30	3.06E-07	9.55E-07	18.3	DMT	CTL	Immunoglobulin-like and fibronectin type III domain-containing protein 1 (Fragment) GN=Igfn1
A0A0N4SUM8	4	3	29.4	0.000215	0.000276	18.1	DMT	CTL	Predicted gene 45140 GN=Gm45140
**G3V011**	**7**	**2**	**46**	**0.319898**	**0.13853**	**18**	**DMT**	**CTL**	**Unconventional myosin-If GN=Myo1f**
Q3ULB1	2	2	10.1	0.113033	0.059	18	DMT	CTL	Testin GN=Tes
P59114	6	4	39.6	0.009768	0.007409	17.8	DMT	CTL	mRNA (2'-O-methyladenosine-N(6)-)-methyltransferase GN=Pcif1
G5E8L2	3	2	16.7	2.34E-11	2.83E-10	17.7	DMT	CTL	Nucleolysin TIA-1 GN=Tia1
**Q5CZY9**	**15**	**4**	**122**	**2.83E-09**	**1.64E-08**	**17.6**	**CTL**	**DMT**	**Rps16 protein GN=Rps16**
P16882	4	2	27.7	2.72E-09	1.58E-08	17.5	CTL	DMT	Growth hormone receptor GN=Ghr
Q3UFM6	2	2	10.3	2.26E-05	3.99E-05	17	CTL	DMT	Splicing factor ESS-2 homolog GN=Ess2
**E9Q9F7**	**19**	**2**	**119**	**6.02E-07**	**1.7E-06**	**17**	**DMT**	**CTL**	**Coiled-coil domain-containing 146 GN=Ccdc146**
**Q3UJU3**	**14**	**3**	**84.3**	**0.000331**	**0.000402**	**16.9**	**DMT**	**CTL**	**E3 ubiquitin-protein ligase GN=Wwp2**
Q9D2H5	4	2	25.6	2.63E-13	6.34E-12	16.9	DMT	CTL	Tripartite motif-containing protein 42 GN=Trim42
**A0A1L1SS82**	**8**	**2**	**45.2**	**4.83E-06**	**1.03E-05**	**16.5**	**DMT**	**CTL**	**Coiled-coil domain-containing 7A GN=Ccdc7a**
**A6PWD2**	**40**	**10**	**238**	**1.17E-10**	**1.08E-09**	**16.5**	**CTL**	**DMT**	**Forkhead-associated domain-containing protein 1 GN=Fhad1**
**E9Q043**	**13**	**7**	**67.9**	**1.66E-09**	**1.06E-08**	**16.4**	**CTL**	**DMT**	**Fibronectin type III domain-containing 1 GN=Fndc1**
**A2RSY1**	**11**	**3**	**74.2**	**2.89E-05**	**4.96E-05**	**16.3**	**DMT**	**CTL**	**KAT8 regulatory NSL complex subunit 3 GN=Kansl3**
P35821	4	2	20.1	1.46E-05	2.73E-05	16.1	DMT	CTL	Tyrosine-protein phosphatase non-receptor type 1 GN=Ptpn1
**P48722**	**19**	**5**	**123**	**4.2E-12**	**6.29E-11**	**16.1**	**CTL**	**DMT**	**Heat shock 70 kDa protein 4L GN=Hspa4l**
**Q3UZ18**	**8**	**2**	**50.9**	**2E-05**	**3.59E-05**	**15.6**	**DMT**	**CTL**	**Little elongation complex subunit 2 GN=Ice2**
Q9R1L5	3	2	13	0.000267	0.000335	15.6	CTL	DMT	Microtubule-associated serine/threonine-protein kinase 1 GN=Mast1
D3Z5P0	3	2	16.3	1.85E-07	6.15E-07	15.3	DMT	CTL	Serine/threonine-protein kinase BRSK1 GN=Brsk1
P28828	6	3	30.2	2.48E-12	4.05E-11	15.2	DMT	CTL	Receptor-type tyrosine-protein phosphatase mu GN=Ptprm
**G3UX23**	**8**	**3**	**62.1**	**9.7E-05**	**0.000139**	**15.1**	**DMT**	**CTL**	**Bifunctional UDP-N-acetylglucosamine 2-epimerase/N-acetylmannosamine kinase GN=Gne**
Q3UJM1	6	2	32.8	2.6E-06	6.03E-06	15.1	DMT	CTL	Uncharacterized protein GN=Trmt2a
**Q3TM31**	**9**	**2**	**43.2**	**0.043612**	**0.026424**	**14.8**	**DMT**	**CTL**	**TIR domain-containing protein GN=Tlr3**
D3YWJ9	3	2	20.7	0.0001	0.000143	14.6	DMT	CTL	Ras-associating and dilute domain-containing protein GN=Radil

E9Q980	6	2	29.6	0.003349	0.00295	14.5	CTL	DMT	Zinc finger protein 788 GN=Zfp788
A0A1Y7VN40	3	2	15	6.57E-05	0.000101	14.5	DMT	CTL	Predicted gene_ 30302 GN=Gm30302
**Q4VAA2**	**11**	**3**	**86.8**	**0.000251**	**0.000317**	**14.4**	**DMT**	**CTL**	**Protein CDV3 GN=Cdv3**
Q9JL35	8	2	37.8	0.004234	0.003618	14.3	CTL	DMT	High mobility group nucleosome-binding domain-containing protein 5 GN=Hmgn5
Q9QZS0	6	2	33.2	1.17E-06	3E-06	14.3	DMT	CTL	Collagen alpha-3(IV) chain GN=Col4a3
**P11031**	**8**	**2**	**74.5**	**0.000382**	**0.000454**	**14.2**	**CTL**	**DMT**	**Activated RNA polymerase II transcriptional coactivator p15 GN=Sub1**
A2AWP6	7	4	33.6	0.061162	0.035085	14.1	DMT	CTL	Rho guanine nucleotide exchange factor 10-like protein GN=Arhgef10l
**Q6P5D8**	**36**	**15**	**219**	**2.07E-08**	**9.1E-08**	**14.1**	**CTL**	**DMT**	**Structural maintenance of chromosomes flexible hinge domain-containing protein 1 GN=Smchd1**
**A4GZ26**	**18**	**3**	**113**	**4.3E-06**	**9.34E-06**	**14**	**CTL**	**DMT**	**ARF6 guanine nucleotide exchange factor IQArfGEF GN=Iqsec2**
**A0A140T8S1**	**9**	**2**	**59.4**	**3.99E-05**	**6.55E-05**	**14**	**CTL**	**DMT**	**A-kinase anchor protein 9 (Fragment) GN=Akap9**
**G3UWH9**	**7**	**3**	**43.8**	**4.27E-08**	**1.68E-07**	**13.9**	**DMT**	**CTL**	**Conserved oligomeric Golgi complex subunit 4 (Fragment) GN=Cog4**
**Q8BUR4**	**11**	**5**	**60.1**	**2.23E-08**	**9.74E-08**	**13.8**	**CTL**	**DMT**	**Dedicator of cytokinesis protein 1 GN=Dock1**
**Q3UTF4**	**8**	**2**	**58.2**	**0.028192**	**0.018189**	**13.8**	**DMT**	**CTL**	**Zinc finger protein 114 GN=Zfp114**
B2RUH7	6	2	32.5	0.014046	0.010085	13.8	CTL	DMT	Protocadherin beta 2 GN=Pcdhb2
**A2AT37**	**21**	**5**	**127**	**1.21E-10**	**1.1E-09**	**13.8**	**DMT**	**CTL**	**Regulator of nonsense transcripts 2 GN=Upf2**
A0A5F8MPP1	7	2	36.8	0.049383	0.029308	13.7	DMT	CTL	RIKEN cDNA C530008M17 gene GN=C530008M17Rik
**Q78NA6**	**16**	**5**	**95.2**	**2.91E-13**	**6.9E-12**	**13.5**	**DMT**	**CTL**	**V(D)J recombination-activating protein 1 GN=Rag1**
**Q3UDU4**	**41**	**6**	**474**	**7.91E-05**	**0.000118**	**13.5**	**CTL**	**DMT**	**L-lactate dehydrogenase GN=Ldha**
**Q7TS74**	**11**	**3**	**72.6**	**0.037331**	**0.023225**	**13.4**	**DMT**	**CTL**	**Cytoskeleton-associated protein 2-like GN=Ckap2l**
E0CXC2	4	2	17.5	1.83E-12	3.24E-11	13.3	DMT	CTL	E3 ubiquitin-protein ligase RNF8 GN=Gm28043
**B1ASZ3**	**9**	**2**	**45.8**	**0.037514**	**0.023304**	**13.2**	**DMT**	**CTL**	**Glycerol kinase GN=Gk**
Q5FWI3	5	2	29.4	2.25E-07	7.36E-07	13.1	DMT	CTL	Cell surface hyaluronidase GN=Cemip2
J3QML2	5	2	27	0	0	13	DMT	CTL	Canalicular multispecific organic anion transporter 2 GN=Abcc3
**P02802**	**7**	**3**	**66.1**	**0.004504**	**0.003806**	**12.8**	**CTL**	**DMT**	**Metallothionein-1 GN=Mt1**
Q8CFL8	5	2	27.1	2.74E-05	4.74E-05	12.8	CTL	DMT	Zinc finger SWIM domain-containing protein 3 GN=Zswim3
**A0A1Y7VME9**	**15**	**4**	**93.5**	**4.62E-10**	**3.48E-09**	**12.8**	**CTL**	**DMT**	**Kinase D-interacting substrate 220 GN=Kidins220**
**Q499F8**	**9**	**2**	**63**	**9.76E-06**	**1.93E-05**	**12.8**	**DMT**	**CTL**	**Zinc finger protein 445 GN=Zfp445**
G3XA14	3	2	17.3	4.99E-06	1.06E-05	12.7	DMT	CTL	Aldo-keto reductase family 1_ member C-like GN=Akr1cl
**A2A7F4**	**10**	**3**	**51.1**	**0.000105**	**0.000148**	**12.6**	**DMT**	**CTL**	**Rearranged L-myc fusion sequence GN=Rlf**
**P06800**	**6**	**2**	**41.1**	**6.11E-07**	**1.72E-06**	**12.5**	**DMT**	**CTL**	**Receptor-type tyrosine-protein phosphatase C GN=Ptprc**
**E9Q0A4**	**39**	**4**	**216**	**0.000263**	**0.000331**	**12.4**	**CTL**	**DMT**	**Kinesin-like protein KIF21B GN=Kif21b**
**B7ZCQ3**	**5**	**3**	**49**	**5.67E-05**	**8.94E-05**	**12.4**	**DMT**	**CTL**	**Non-histone chromosomal protein HMG-17 GN=Hmgn2**
**Q499M4**	**6**	**2**	**45.2**	**1.08E-09**	**7.36E-09**	**12.4**	**DMT**	**CTL**	**Tigger transposable element derived 5 GN=Tigd5**
A0A571BE33	3	2	15	6.72E-14	1.9E-12	12.4	DMT	CTL	von Willebrand factor A domain containing 3B GN=Vwa3b
F6S7U1	7	4	35.7	1.59E-06	3.95E-06	12.3	DMT	CTL	RAB6A-GEF complex partner protein 1 (Fragment) GN=Ric1
E9PZJ4	6	2	34.3	1.13E-07	3.94E-07	12.3	DMT	CTL	Bifunctional heparan sulfate N-deacetylase/N-sulfotransferase 3 GN=Ndst3
**Q9QWI6**	**15**	**2**	**93.8**	**2.31E-10**	**1.98E-09**	**12.2**	**DMT**	**CTL**	**SRC kinase signaling inhibitor 1 GN=Srcin1**
**Q9Z1Z0**	**9**	**4**	**50.5**	**2.63E-11**	**3.09E-10**	**12.2**	**DMT**	**CTL**	**General vesicular transport factor p115 GN=Uso1**
**S4R2T7**	**11**	**6**	**75.2**	**3.53E-11**	**4.03E-10**	**12.2**	**DMT**	**CTL**	**Ankyrin-2 (Fragment) GN=Ank2**
**B1AWQ3**	**8**	**3**	**43.1**	**1.2E-10**	**1.1E-09**	**12.2**	**DMT**	**CTL**	**Espin GN=Espn**
Q5NCH9	2	2	12.9	4.26E-05	6.93E-05	12.1	DMT	CTL	Metabotropic glutamate receptor 6 GN=Grm6
D3YZ32	2	2	10.1	2.77E-08	1.17E-07	12.1	CTL	DMT	Mitochondrial fission 1 protein GN=Fis1
A2AQJ8	3	2	14.5	4.12E-11	4.55E-10	12	DMT	CTL	Neutral alpha-glucosidase C GN=Ganc
**Q9CX34**	**13**	**3**	**99.2**	**1.01E-10**	**9.5E-10**	**12**	**DMT**	**CTL**	**Protein SGT1 homolog GN=Sugt1**
Q9WUF3	28	10	171	4.59E-07	1.34E-06	11.9	DMT	CTL	CASP8-associated protein 2 GN=Casp8ap2
**A0A3B2WCI5**	**5**	**2**	**41.7**	**9.53E-13**	**2.04E-11**	**11.8**	**CTL**	**DMT**	**BICD family-like cargo adapter 2 GN=Bicdl2**
**I7HJQ9**	**8**	**5**	**43.6**	**4.71E-06**	**1.01E-05**	**11.5**	**DMT**	**CTL**	**Myotubularin-related protein 1 GN=Mtmr1**
**B2RWU3**	**16**	**3**	**96.1**	**2.2E-07**	**7.2E-07**	**11.5**	**CTL**	**DMT**	**Fanconi anemia_ complementation group A GN=Fanca**
**A2A977**	**12**	**2**	**79.5**	**8.16E-07**	**2.22E-06**	**11.5**	**DMT**	**CTL**	**Cytochrome P450 4A10 GN=Cyp4a10**
**A0A0R4J2C1**	**9**	**5**	**52.1**	**0.002787**	**0.002517**	**11.5**	**DMT**	**CTL**	**Amyloid-beta A4 precursor protein-binding family B member 1 GN=Apbb1**
**Q05DR6**	**9**	**3**	**46.7**	**1.06E-13**	**2.76E-12**	**11.4**	**DMT**	**CTL**	**Cul1 protein (Fragment) GN=Cul1**
D3YTU0	2	2	10.9	8.34E-11	8.32E-10	11.3	DMT	CTL	Vesicle-associated membrane protein 1 GN=Vamp1

**E9PXZ7**	**15**	**6**	**90.8**	**2.11E-15**	**9.48E-14**	**11.1**	**CTL**	**DMT**	**Integrin alpha-D GN=Itgad**
Q2TBA4	6	3	32.2	3.02E-05	5.14E-05	11.1	DMT	CTL	Bnc2 protein GN=Bnc2
**A0A2I3BR72**	**14**	**3**	**105**	**9.3E-13**	**2.02E-11**	**11**	**CTL**	**DMT**	**Roundabout homolog 2 GN=Robo2**
**P70355**	**59**	**2**	**392**	**9.11E-11**	**8.74E-10**	**10.9**	**CTL**	**DMT**	**Gag-Pol polyprotein GN=Mela**
**A7XUZ6**	**28**	**2**	**158**	**6.52E-12**	**9.14E-11**	**10.9**	**CTL**	**DMT**	**Selection and upkeep of intraepithelial T-cells protein 6 GN=Skint6**
Q9D2D7	3	3	14.3	0.016019	0.011303	10.9	CTL	DMT	Zinc finger protein 687 GN=Znf687
**B9EJ54**	**26**	**2**	**141**	**0.000214**	**0.000276**	**10.9**	**DMT**	**CTL**	**Nucleoporin 205 GN=Nup205**
Q148S3	2	2	16.9	0.03231	0.020511	10.8	DMT	CTL	RNA-binding protein NOB1 GN=Nob1
**Q9CZU6**	**22**	**3**	**188**	**8.81E-11**	**8.58E-10**	**10.8**	**CTL**	**DMT**	**Citrate synthase_ mitochondrial GN=Cs**
Q059Y8	4	2	20.6	2.27E-10	1.96E-09	10.8	CTL	DMT	E3 ubiquitin-protein ligase DCST1 GN=Dcst1
**B1AXW5**	**28**	**3**	**280**	**0.010465**	**0.007873**	**10.7**	**CTL**	**DMT**	**Peroxiredoxin-1 (Fragment) GN=Prdx1**
**A0A0R4IZX1**	**10**	**2**	**66.5**	**0.00019**	**0.000247**	**10.6**	**CTL**	**DMT**	**Differentially-expressed in FDCP 6 GN=Def6**
**P62900**	**6**	**2**	**58.7**	**1.57E-05**	**2.9E-05**	**10.5**	**DMT**	**CTL**	**60S ribosomal protein L31 GN=Rpl31**
Q62460	3	3	16.5	5.88E-10	4.21E-09	10.5	DMT	CTL	(clone BALB3N) GN=Gm20865
**Q3UK56**	**21**	**2**	**201**	**1.04E-08**	**5.05E-08**	**10.5**	**DMT**	**CTL**	**KH type-2 domain-containing protein GN=Rps3**
**Q6ZPY5**	**9**	**2**	**64.4**	**0.021006**	**0.014206**	**10.4**	**DMT**	**CTL**	**Zinc finger protein 507 GN=Znf507**
**E9QNV2**	**6**	**2**	**47**	**1.04E-06**	**2.73E-06**	**10.4**	**DMT**	**CTL**	**Mediator of RNA polymerase II transcription subunit 23 GN=Med23**
D3Z6X4	2	2	10.9	4.19E-05	6.83E-05	10.4	DMT	CTL	Peroxisomal biogenesis factor 3 GN=Pex3
Q8CDU5	6	3	38.2	9.45E-09	4.7E-08	10.2	DMT	CTL	EF-hand domain-containing family member B GN=Efhb
**Q91W86**	**9**	**3**	**51**	**1.14E-07**	**3.96E-07**	**10.2**	**DMT**	**CTL**	**Vacuolar protein sorting-associated protein 11 homolog GN=Vps11**
P43688	3	2	16.4	0.013657	0.009838	10.2	DMT	CTL	Homeobox protein Nkx-2.6 GN=Nkx2-6
F6UFZ5	5	3	25.7	0.002199	0.002048	10.1	DMT	CTL	PCF11 cleavage and polyadenylation factor subunit (Fragment) GN=Pcf11

Table S5: Properties of the identified proteins following direct microglia treatment (DMT) in mouse primary neuron (MPN) in comparison with untreated cells. The properties were identified by Progenesis Software after the LC-MS analysis.

Accession	Peptides	Unique peptides	Confidence score	Anova (p)	q Value	Max fold change	Highest Mean	Lowest Mean	Description
P63276	3	1	16.5	6.18E-10	2.50E-07	α	NOT	DMT	40S ribosomal protein S17 GN=Rps17
A0A0R4J288	4	2	34.6	6.43E-06	0.000371	2.95	DMT	NOT	Decapping and exoribonuclease protein GN=Dxo
E9PUA2	6	3	33.3	8.19E-06	0.000414	2.34	DMT	NOT	Terminal uridylyltransferase 7 GN=Tut7
A0A286YE28	14	1	116	2.49E-05	0.000876	1.97	NOT	DMT	Transketolase (Fragment) GN=Tkt
E9Q3V9	6	6	26.3	3.65E-05	0.001053	3.9	NOT	DMT	Adhesion G protein-coupled receptor L1 GN=Adgrl1
A0A5F8MPU7	5	3	29.3	4.08E-05	0.0011	1.81	DMT	NOT	[F-actin]-monooxygenase MICAL3 GN=Mical3
A0A0R4J0A4	4	4	15.6	8.51E-05	0.001912	10.3	NOT	DMT	Vascular endothelial growth factor receptor 1 GN=Flt1
Q6ZQ14	3	3	27	0.000163	0.00275	11.4	DMT	NOT	MKIAA0971 protein (Fragment) GN=mKIAA0971
P97872	2	2	15.6	0.000257	0.004162	32.4	DMT	NOT	Dimethylaniline monooxygenase [N-oxide-forming] 5 GN=Fmo5
Q810Y8	6	4	35	0.000443	0.006395	5.27	DMT	NOT	Preferentially expressed antigen in melanoma-like protein 7 GN=Pramel7
A0A0R4J0F6	5	4	33.5	0.000555	0.007477	2.81	DMT	NOT	Cyclin-G-associated kinase GN=Gak
B2RU82	4	2	23.5	0.000855	0.010518	3.85	DMT	NOT	Solute carrier family 30 (Zinc transporter)_ member 9 GN=Slc30a9
A2AUM9	8	4	43.4	0.001114	0.013246	2.71	DMT	NOT	Centrosomal protein of 152 kDa GN=Cep152
A2RTV8	3	2	21.6	0.001189	0.013736	3.4	DMT	NOT	Prorelaxin 1 GN=Rln1
A0A286YCM2	3	3	19.6	0.001271	0.014274	6	NOT	DMT	Heterogeneous nuclear ribonucleoprotein K (Fragment) GN=Hnrnpk
Q3URY5	3	2	17.7	0.001413	0.014738	3.49	NOT	DMT	Zinc finger protein 941 GN=Zfp941
A2AVR6	4	2	24.7	0.00191	0.019076	5.13	DMT	NOT	Acyl-coenzyme A thioesterase 11 GN=Acot11
Q3TD08	6	6	32	0.001982	0.019076	2.86	DMT	NOT	Uncharacterized protein GN=Ndrg1
Q3U9P7	4	3	29.2	0.002041	0.019181	4.45	NOT	DMT	Succinyl-CoA:3-ketoacid-coenzyme A transferase GN=Oxct1
Q78NA6	5	3	22.3	0.002166	0.019566	2.98	NOT	DMT	V(D)J recombination-activating protein 1 GN=Rag1
Q69ZK1	5	3	22.5	0.002666	0.023421	2.7	DMT	NOT	MKIAA1413 protein (Fragment) GN=N4bp2
P62900	2	1	20.1	0.002978	0.024101	3.65	NOT	DMT	60S ribosomal protein L31 GN=Rpl31
Q7M6X7	3	2	16.4	0.003041	0.024101	1.93	DMT	NOT	Zinc finger protein 457 GN=Zfp457
Q3U430	4	3	21.6	0.003273	0.025437	7.92	DMT	NOT	Uncharacterized protein GN=Anapc2
F8WIC0	5	3	23.8	0.003479	0.026535	3.76	DMT	NOT	Adenylate kinase 7 GN=Ak7
B2RSU7	6	2	44.1	0.004193	0.030322	2.44	DMT	NOT	GRIP and coiled-coil domain containing 2 GN=Gcc2
S4R257	26	4	259	0.004201	0.030322	1.55	DMT	NOT	Glyceraldehyde-3-phosphate dehydrogenase (Fragment) GN=Gapdh
A2A9K7	3	2	15	0.005229	0.035822	3.72	DMT	NOT	Connector enhancer of kinase suppressor of Ras 1 GN=Cnksr1
P19426	19	5	97.7	0.006039	0.038778	2.62	DMT	NOT	Negative elongation factor E GN=Nelfe
P80316	8	4	49.9	0.006107	0.038778	2.14	NOT	DMT	T-complex protein 1 subunit epsilon GN=Cct5
Q3TST4	6	4	30.4	0.006236	0.038778	3.25	DMT	NOT	G protein-coupled receptor kinase GN=Grk5
F6ZSB7	9	3	62.7	0.006772	0.04085	2.29	DMT	NOT	D-3-phosphoglycerate dehydrogenase (Fragment) GN=Phgdh
Q9CSE2	2	2	15.2	0.007384	0.043341	3.17	DMT	NOT	Uncharacterized protein (Fragment) GN=Zfp397
A0A2I3BPI0	4	1	21.9	0.007465	0.043341	9.02	DMT	NOT	Coiled-coil domain-containing protein 187 GN=Ccdc187
F8WI35	5	5	39.8	0.007605	0.043341	1.85	DMT	NOT	Histone H3 GN=H3f3a
O08553	7	4	42	0.007613	0.043341	4.04	DMT	NOT	Dihydropyrimidinase-related protein 2 GN=Dpysl2
B1ARR7	15	2	188	0.009072	0.049519	1.38	NOT	DMT	Alpha-enolase (Fragment) GN=Eno1
G5E8Y1	7	6	35.2	0.009434	0.049519	1.72	DMT	NOT	Zinc finger protein 426 GN=Zfp426
Q80Z20	4	2	28.1	0.009598	0.049738	1.52	DMT	NOT	Secreted gel-forming mucin (Fragment)
E7CHD7	3	2	17.5	0.009869	0.050494	10.8	NOT	DMT	RPGRIP1 variant-11aa GN=Rpgrip1
Q3UV17	2	2	15	0.010234	0.051065	2.92	NOT	DMT	Keratin_ type II cytoskeletal 2 oral GN=Krt76

A0A140LI36	14	13	62.3	0.010597	0.052231	1.53	DMT	NOT	Androglobin GN=Adgb
P62918	5	5	49.1	0.010732	0.052263	1.92	DMT	NOT	60S ribosomal protein L8 GN=Rpl8
B1ASE2	4	3	23.8	0.012482	0.058663	2.46	DMT	NOT	ATP synthase_ H+-transporting_ mitochondrial F0 complex_ subunit D (Fragment) GN=Atp5h
Q3URQ0	3	3	19.9	0.012724	0.059111	2.55	DMT	NOT	Testis-expressed protein 10 GN=Tex10
Q61916	3	1	24.4	0.013162	0.059773	1.5	DMT	NOT	Mucin (Fragment) GN=Muc5ac
A0A571BEW5	3	2	19.3	0.013879	0.061478	2.94	DMT	NOT	Lysophosphatidic acid receptor 2 GN=Lpar2
B7ZP22	11	10	74.8	0.014216	0.061478	1.41	DMT	NOT	Heterogeneous nuclear ribonucleoprotein A2/B1 GN=Hnrnpa2b1
Q3TWE3	4	2	24.3	0.01455	0.061478	5.09	NOT	DMT	Protein disulfide-isomerase GN=P4hb
A0A1W2P6F6	4	3	31.1	0.014586	0.061478	1.63	DMT	NOT	Myosin light polypeptide 6 GN=Myl6
Q6DI58	4	3	27.9	0.014602	0.061478	3.48	NOT	DMT	Rpl12 protein (Fragment) GN=Rpl12
Q3UBS0	7	5	38.8	0.015268	0.062969	2.15	NOT	DMT	Uncharacterized protein GN=Apoe
C3S7Q5	3	2	17.9	0.017038	0.068865	2.09	DMT	NOT	Ojoplano variant A GN=Ofcc1
A2ALW2	6	5	32	0.018014	0.07138	3.33	DMT	NOT	Zinc finger with KRAB and SCAN domains 16 GN=Zkscan16
Q6P9L6	4	2	25.6	0.018714	0.072728	3.65	NOT	DMT	Kinesin-like protein KIF15 GN=Kif15
P03995	6	4	44.9	0.020878	0.077312	1.61	DMT	NOT	Glial fibrillary acidic protein GN=Gfap
A0A0A6YWM5	4	2	16.2	0.021041	0.077312	3.06	DMT	NOT	Rab3 GTPase-activating protein non-catalytic subunit GN=Rab3gap2
Q571K1	7	4	30	0.02129	0.077478	7.47	NOT	DMT	MKIAA4151 protein (Fragment) GN=Golgb1
P17751	11	11	83.4	0.021638	0.077478	2.2	NOT	DMT	Triosephosphate isomerase GN=Tpi1
Q4FJT0	18	9	128	0.021661	0.077478	1.68	DMT	NOT	Matr3 protein GN=Matr3
Q3U741	6	4	33.9	0.023329	0.081287	1.65	NOT	DMT	Probable ATP-dependent RNA helicase DDX17 GN=Ddx17
Q6ZPZ4	4	2	15.5	0.024029	0.082914	3.07	DMT	NOT	MKIAA1062 protein (Fragment) GN=Abca2
P98203	3	1	21.5	0.024655	0.082914	8.23	DMT	NOT	Armadillo repeat protein deleted in velo-cardio-facial syndrome homolog GN=Arvcf
G3UW90	2	1	20.5	0.024814	0.082914	4.22	NOT	DMT	Zinc finger transcription factor Trps1 GN=Trps1
Z4YJU8	5	4	31.4	0.026247	0.086248	2.56	DMT	NOT	Golgin subfamily A member 2 (Fragment) GN=Golga2
B9EIX2	4	2	26.8	0.027392	0.089284	4.35	DMT	NOT	AW555464 protein GN=Cep170b
Q91YP3	3	2	22.5	0.029202	0.092936	1.7	DMT	NOT	Deoxyribose-phosphate aldolase GN=Dera
V9GXQ2	3	2	26.9	0.029677	0.092984	2.44	DMT	NOT	Predicted gene 17087 GN=Gm17087
A0A0R4J036	18	13	105	0.032106	0.097609	2.8	DMT	NOT	Neurofilament medium polypeptide GN=Nefm
A1L340	9	8	49.5	0.032336	0.097609	1.32	DMT	NOT	Retinitis pigmentosa 1 homolog (Human)-like 1 GN=Rp1l1
Q8BK57	3	2	19.9	0.032927	0.098581	4.02	DMT	NOT	Uncharacterized protein GN=Eif2b2
E9Q3I8	14	1	81.7	0.033554	0.099721	1.54	DMT	NOT	Intersectin-1 GN=Itsn1
G3X923	3	1	20.2	0.034842	0.102792	22.9	DMT	NOT	Transmembrane protein 184A GN=Tmem184a
P60843	3	2	17.3	0.035202	0.103101	24.9	NOT	DMT	Eukaryotic initiation factor 4A-I GN=Eif4a1
A0A1S6GWJ9	2	1	22.2	0.036047	0.104228	94.4	DMT	NOT	Elongation factor Ts_ mitochondrial GN=Tsfm
Q922A0	8	1	71.6	0.036102	0.104228	2.02	DMT	NOT	Eno2 protein (Fragment) GN=Eno2
Q3TXL0	4	4	22.8	0.036968	0.105223	2.64	NOT	DMT	Uncharacterized protein (Fragment) GN=Ero1l
F8WIJ0	5	3	38.9	0.037833	0.106933	1.95	DMT	NOT	Solute carrier family 12 member 4 GN=Slc12a4
A0A1W2P712	5	5	33.8	0.040362	0.110108	1.72	NOT	DMT	Ral GTPase-activating protein subunit alpha-1 GN=Ralgapa1
B2RWH3	4	1	43.7	0.04043	0.110108	1.95	DMT	NOT	Histone H2A GN=Hist2h2aa1
A0A2R8VHP3	4	2	32	0.040982	0.110108	2.72	NOT	DMT	Predicted pseudogene 5478 GN=Gm5478
Q99LX0	7	7	58.3	0.044158	0.117369	1.36	NOT	DMT	Protein/nucleic acid deglycase DJ-1 GN=Park7
Q8CF78	23	2	289	0.044429	0.117369	1.58	DMT	NOT	Uncharacterized protein
Q8CGF4	4	3	22.3	0.045309	0.118153	2.59	NOT	DMT	Sec24 related gene family_ member C (S. cerevisiae) GN=Sec24c
Q66JU1	2	1	16.5	0.04531	0.118153	1.98	DMT	NOT	Eif3c protein (Fragment) GN=Eif3c
F8VQJ3	6	4	25.9	0.04833	0.122539	2.36	DMT	NOT	Laminin subunit gamma-1 GN=Lamc1
Q9D6X8	2	1	20.5	0.048508	0.122539	3.66	DMT	NOT	Zinc finger with KRAB and SCAN domains 14 GN=Zkscan14
P99024	13	1	108	0.049876	0.12521	2.37	NOT	DMT	Tubulin beta-5 chain GN=Tubb5

Table S6: Properties of the identified proteins following direct microglia treatment (DMT) in mouse primary neuron (MPN) in comparison with 3F4 antibody treated cells. The properties were identified by Progenesis Software after the LC-MS analysis.

Accession	Peptides	Unique peptides	Confidence score	Anova (p)	q Value	Max fold change	Highest Mean	Lowest Mean	Description
G3V011	7	7	33.8	0.000555	0.105665	2.48	3F4	DMT	Unconventional myosin-If GN=Myo1f
Q78NA6	5	3	22.3	0.000645	0.105665	4.79	3F4	DMT	V(D)J recombination-activating protein 1 GN=Rag1
P03995	6	4	44.9	0.000789	0.105665	1.67	DMT	3F4	Glial fibrillary acidic protein GN=Gfap
E9QA15	8	6	41.7	0.000895	0.105665	2.83	DMT	3F4	Caldesmon 1 GN=Cald1
A1L340	9	8	49.5	0.003143	0.222686	1.52	DMT	3F4	Retinitis pigmentosa 1 homolog (Human)-like 1 GN=Rp1l1
S4R257	26	4	259	0.004001	0.23649	1.4	DMT	3F4	Glyceraldehyde-3-phosphate dehydrogenase (Fragment) GN=Gapdh
O08553	7	4	42	0.004005	0.23649	1.4	DMT	3F4	Dihydropyrimidinase-related protein 2 GN=Dpysl2
P62900	2	1	20.1	0.00594	0.29811	4.49	3F4	DMT	60S ribosomal protein L31 GN=Rpl31
Q6DI58	4	3	27.9	0.006311	0.29811	4.09	3F4	DMT	Rpl12 protein (Fragment) GN=Rpl12
Q6PDG0	6	3	23.6	0.00859	0.35802	1.84	DMT	3F4	Nup205 protein (Fragment) GN=Nup205
Q3U741	6	4	33.9	0.009882	0.358526	1.72	DMT	3F4	Probable ATP-dependent RNA helicase DDX17 GN=Ddx17
Q8K3I4	3	2	18.3	0.00994	0.358526	1.81	DMT	3F4	Rab effector MyRIP GN=Myrip
B1ASE2	4	3	23.8	0.01087	0.358526	2.3	DMT	3F4	ATP synthase_ H+-transporting_ mitochondrial F0 complex_ subunit D (Fragment) GN=Atp5h
A0A0R4J1Q0	6	5	37.1	0.011197	0.358526	1.62	3F4	DMT	Enhancer of mRNA-decapping protein 4 GN=Edc4
F6ZSB7	9	3	62.7	0.012408	0.358526	2.09	DMT	3F4	D-3-phosphoglycerate dehydrogenase (Fragment) GN=Phgdh
B2RU82	4	2	23.5	0.013601	0.358526	2.25	DMT	3F4	Solute carrier family 30 (Zinc transporter)_ member 9 GN=Slc30a9
P62918	5	5	49.1	0.013603	0.358526	1.7	DMT	3F4	60S ribosomal protein L8 GN=Rpl8
E9PUA2	6	3	33.3	0.013742	0.358526	1.5	3F4	DMT	Terminal uridylyltransferase 7 GN=Tut7
B1AQX6	4	2	19.2	0.014264	0.358526	2.11	DMT	3F4	SRC kinase-signaling inhibitor 1 GN=Srcin1
A0A1S6GWJ8	2	1	15.3	0.015578	0.358526	3.97	3F4	DMT	Uncharacterized protein GN=Hnrnpm
B1ARR7	15	2	188	0.01661	0.358526	1.35	DMT	3F4	Alpha-enolase (Fragment) GN=Eno1
G3UZR1	4	1	41.2	0.017732	0.358526	12.7	3F4	DMT	Tubulin beta-5 chain (Fragment) GN=Tubb5
Q8BML7	3	2	17.5	0.018228	0.358526	1.91	DMT	3F4	Poly [ADP-ribose] polymerase (Fragment) GN=Parp11
D3YTR0	4	3	20.9	0.01895	0.358526	3.48	DMT	3F4	Adenomatous polyposis coli protein 2 GN=Apc2
P62082	5	5	39.4	0.019383	0.358526	1.4	3F4	DMT	40S ribosomal protein S7 GN=Rps7
Q8CH65	9	2	56.8	0.020113	0.358526	8.92	DMT	3F4	BIRC1E protein GN=Naip5
Q920P5	3	3	17.2	0.020241	0.358526	7.65	DMT	3F4	Adenylate kinase isoenzyme 5 GN=Ak5
A0A140LI36	14	13	62.3	0.020881	0.360836	1.56	DMT	3F4	Androglobin GN=Adgb
F6XAI7	2	1	15.2	0.022444	0.369804	2.72	3F4	DMT	EF-hand calcium-binding domain-containing protein 7 GN=Efcab7
Q546G4	29	26	194	0.023819	0.374875	2.29	DMT	3F4	Serum albumin GN=Alb
P16045	3	3	20	0.027397	0.398896	2.06	DMT	3F4	Galectin-1 GN=Lgals1
P15331	8	1	74.7	0.029486	0.398896	1.98	3F4	DMT	Peripherin GN=Prph
P31001	5	2	33.1	0.029545	0.398896	1.79	3F4	DMT	Desmin GN=Des
Q8K441	6	5	29.6	0.03046	0.398896	1.74	3F4	DMT	ATP-binding cassette sub-family A member 6 GN=Abca6
E7CHD7	3	2	17.5	0.030525	0.398896	3.88	3F4	DMT	RPGRIP1 variant-11aa GN=Rpgrip1
A0A087WRB8	7	7	41.9	0.030817	0.398896	2.38	3F4	DMT	Dystonin (Fragment) GN=Dst
B2RSU7	6	2	44.1	0.031281	0.398896	1.79	DMT	3F4	GRIP and coiled-coil domain containing 2 GN=Gcc2
Q91YP3	3	2	22.5	0.032947	0.398896	1.58	DMT	3F4	Deoxyribose-phosphate aldolase GN=Dera
F8VQJ3	6	4	25.9	0.035355	0.398896	2.31	DMT	3F4	Laminin subunit gamma-1 GN=Lamc1
E9PWX1	4	2	18.6	0.03539	0.398896	1.65	DMT	3F4	Dipeptidyl aminopeptidase-like protein 6 GN=Dpp6
Q3UBS0	7	5	38.8	0.03547	0.398896	2.23	3F4	DMT	Uncharacterized protein GN=Apoe
Q5FW97	40	15	472	0.039998	0.435986	1.28	DMT	3F4	Enolase 1_ alpha non-neuron GN=EG433182
O70291	3	1	19.1	0.040921	0.438885	2.12	DMT	3F4	G protein-coupled receptor kinase 4 GN=Grk4
E9Q9E7	5	5	35	0.041503	0.438885	1.7	DMT	3F4	Centriolin GN=Cntrl

A0A286YE28	14	1	116	0.042689	0.444593	1.2	3F4	DMT	Transketolase (Fragment) GN=Tkt
A0A5F8MPU7	5	3	29.3	0.045117	0.444593	1.46	DMT	3F4	[F-actin]-monooxygenase MICAL3 GN=Mical3
Q3UZK5	3	2	19.9	0.045181	0.444593	1.3	DMT	3F4	Uncharacterized protein (Fragment) GN=Akap13
A2AFS0	4	3	24.1	0.048723	0.451406	1.57	3F4	DMT	Serine--tRNA ligase_ cytoplasmic (Fragment) GN=Sars
Z4YJU8	5	4	31.4	0.049059	0.451406	2.25	DMT	3F4	Golgin subfamily A member 2 (Fragment) GN=Golga2

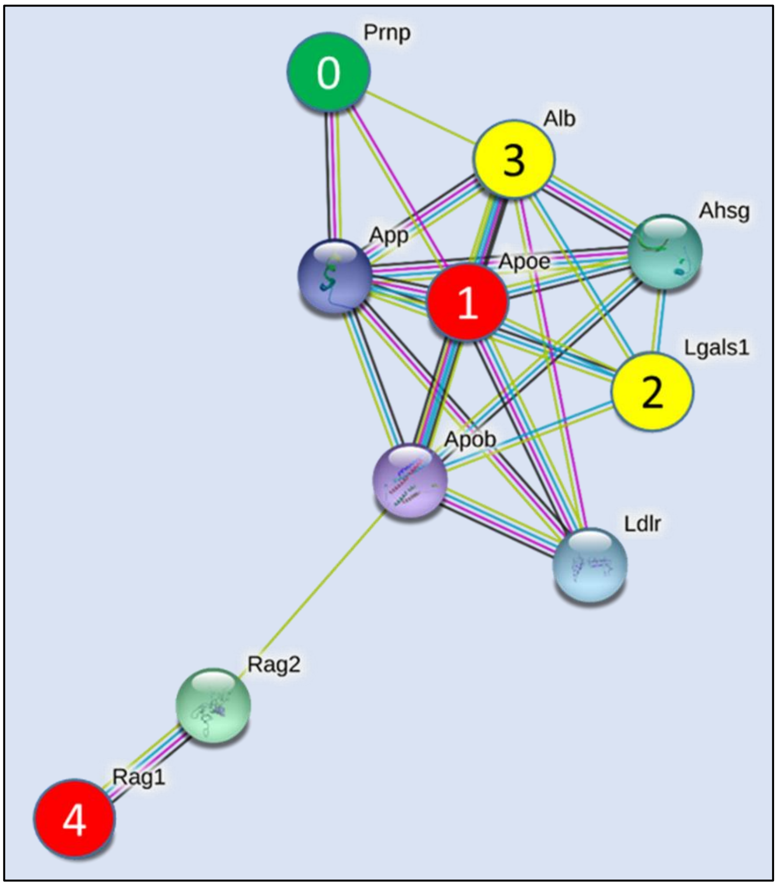

Figure S3: Protein-protein interaction of the identified 4 allergenic genes following direct microglia treatment (DMT) from mouse primary neuron (MPN) in comparison with 3F4 antibody treated cells. Protein-protein interaction analysis was performed using STRING v11.0 server. The *Mus Musculus* database was used as the host organism for the identification of protein interactions. Interaction among the allergenic proteins (numbered 1, 2, 3 & 4) and with PrP^{C} (numbered 0) following direct antibody treatment, where red and yellow color indicate the downregulated and upregulated genes, respectively and green color represents the cellular prion protein PrPC.

Table S7: Properties of the identified proteins following direct antibody treatment to microglia (N11) (Control). The properties were identified by Progenesis Software after the LC-MS analysis.

Accession	Peptides	Unique peptides	Confidence score	Anova (p)	q Value	Max fold change	Highest Mean	Lowest Mean	Description
A0A1D5RLV4	2	1	8.76	2.30E-05	0.004956	37.6	AMT	CTL	Golgin subfamily A member 4 (Fragment) GN=Golga4
A2A7F4	2	1	8.51	0.00108	0.035277	2.07E+03	AMT	CTL	Rearranged L-myc fusion sequence GN=Rlf
D3Z2W9	1	1	4.23	0.022037	0.126289	479	AMT	CTL	Cancer-related nucleoside-triphosphatase homolog GN=Ntpcr
E0CXC1	1	1	5.24	0.044922	0.182062	557	AMT	CTL	Cilia- and flagella-associated protein 206 GN=Cfap206
E0CZ22	2	1	10	1.82E-06	0.000874	32.2	AMT	CTL	Maestro heat-like repeat family member 1 GN=Mroh1
F6TY75	1	1	5.14	0.000859	0.032464	16.8	AMT	CTL	RNA-binding protein MEX3D (Fragment) GN=Mex3d
G3X931	2	1	9.32	0.001436	0.039667	20.8	AMT	CTL	Vomeronasal 2_ receptor 65 GN=Vmn2r65
G3XA59	1	1	4.58	0.019323	0.116306	19.2	AMT	CTL	Transforming growth factor beta activator LRRC32 GN=Lrrc32
O35189	1	1	3.89	0.003149	0.055945	13.2	AMT	CTL	Putative pheromone receptor GN=Vmn2r123
Q3U9U7	1	1	5.28	0.01236	0.088464	38.2	AMT	CTL	PSI domain-containing protein GN=Pttg1ip
Q3U430	1	1	4.69	0.005872	0.065473	α	AMT	CTL	Uncharacterized protein GN=Anapc2
Q4FJL2	1	1	4.13	0.000333	0.022436	153	AMT	CTL	Reticulon GN=Rtn1
Q5SQP1	2	1	9.63	0.013297	0.092428	α	AMT	CTL	HORMA domain-containing protein 2 GN=Hormad2
Q9CWK0	2	1	14.6	0.017752	0.110557	21.8	AMT	CTL	Ribosomal_L14e domain-containing protein GN=Rpl14

Table S8: Properties of the identified proteins following indirect microglia treatment (IMT). The properties were identified by Progenesis Software after the LC-MS analysis. Selected 11 proteins have been highlighted as bold.

Accession	Peptides	Unique peptides	Confidence score	Anova (p)	q Value	Max fold change	Highest Mean	Lowest Mean	Description
Q61833	9	1	58.5	0.001248	0.099667	α	IMT	CTRL	Dolichyl-diphosphooligosaccharide--protein glycosyltransferase subunit 2 GN=Rpn2
Q80XI6	7	1	38.5	0.014107	0.356679	α	IMT	CTRL	Mitogen-activated protein kinase kinase kinase 11 GN=Map3k11
Q69ZL7	3	1	18.2	0.005196	0.229812	α	CTRL	IMT	MKIAA1336 protein (Fragment) GN=Wdr35
Q32P04	18	1	156	0	0	α	CTRL	IMT	Keratin 5 GN=Krt5
Q14CH7	3	1	22.1	0.012012	0.340111	α	IMT	CTRL	Alanine--tRNA ligase_ mitochondrial GN=Aars2
Q8K025	1	1	5.36	0.018876	0.398448	α	IMT	CTRL	GSK-3-binding protein FRAT2 GN=Frat2
Q8BJY1	3	1	16.7	0.005196	0.229812	α	CTRL	IMT	26S proteasome non-ATPase regulatory subunit 5 GN=Psmd5
Q3U0N4	2	1	11.7	0.002914	0.166647	α	IMT	CTRL	Uncharacterized protein GN=Taf1c
G3X9I7	10	1	56.7	4.86E-06	0.003027	α	IMT	CTRL	Zinc finger CCCH-type-containing 12B GN=Zc3h12b
E9PY14	7	1	36.5	0.048028	0.567103	α	IMT	CTRL	Centromere protein F (Fragment) GN=Cenpf
E9PXG7	1	1	5.78	0.043181	0.546739	α	IMT	CTRL	3 beta-hydroxysteroid dehydrogenase/Delta 5-->4-isomerase type 3 GN=Hsd3b3
E9PX52	2	1	18.1	1.05E-05	0.003871	α	IMT	CTRL	Arf-GAP with SH3 domain_ ANK repeat and PH domain-containing protein 2 GN=Asap2
A2AUR3	3	1	14.3	0.010806	0.323802	α	IMT	CTRL	Phosphotriesterase-related protein (Fragment) GN=Pter
A2AM95	1	1	5.31	0.001633	0.123143	α	IMT	CTRL	Emerin GN=Emd
A2AJY2	1	1	4.03	0.001149	0.099291	α	IMT	CTRL	Collagen alpha-1(XV) chain GN=Col15a1
**A0A571BEC4**	**14**	**3**	**96.5**	**0.001168**	**0.099291**	**α**	**IMT**	**CTRL**	**Ligand-dependent nuclear receptor corepressor-like protein GN=Lcorl**
A0A3Q4EH09	1	1	4.41	0.03379	0.498196	α	IMT	CTRL	Vomeronasal type-2 receptor 116 GN=Vmn2r116
**A0A0N4SVP8**	**25**	**2**	**214**	**4.16E-06**	**0.003027**	**α**	**CTRL**	**IMT**	**Eukaryotic translation initiation factor 4A3-like 2 GN=Eif4a3l2**
Q8BM39	1	1	4.61	0.013411	0.350838	27500	IMT	CTRL	Pre-mRNA-splicing factor 18 GN=Prpf18
A0A1Y7VKF5	1	1	5.75	0.048501	0.568532	1850	IMT	CTRL	39S ribosomal protein L32_ mitochondrial GN=Mrpl32
A0A0R4J1V6	4	1	25.6	0.037645	0.522518	1040	IMT	CTRL	Homeodomain-interacting protein kinase 1 GN=Hipk1
A0A338P6P0	2	1	17.6	0.000949	0.095376	900	IMT	CTRL	Leucine-zipper-like transcriptional regulator 1 GN=Lztr1
Q3UZI3	48	1	336	0.025147	0.432245	523	CTRL	IMT	Staphylococcal nuclease domain-containing protein GN=Snd1
B2RUB8	2	1	10.1	2.64E-08	4.37E-05	272	CTRL	IMT	Homeo box C6 GN=Hoxc6
Q9CRB2	4	1	35	0.000355	0.05119	219	IMT	CTRL	H/ACA ribonucleoprotein complex subunit 2 GN=Nhp2
A2AH85	31	1	205	0.024779	0.430826	198	IMT	CTRL	116 kDa U5 small nuclear ribonucleoprotein component GN=Eftud2
**A0A1W2P711**	**8**	**2**	**45.6**	**7.67E-05**	**0.019583**	**148**	**IMT**	**CTRL**	**Melanoma inhibitory activity protein 2 GN=Mia2**
B1AW75	3	1	15	0.00483	0.219487	139	IMT	CTRL	Fanconi anemia group B protein homolog GN=Fancb
J3QN85	4	1	19.3	0.003099	0.174245	134	IMT	CTRL	Fibroblast growth factor receptor GN=Fgfr1
P19324	3	1	16.5	0.020744	0.40704	97.3	IMT	CTRL	Serpin H1 GN=Serpinh1
H3BLP7	12	1	117	0.032229	0.492698	59.6	CTRL	IMT	Heterogeneous nuclear ribonucleoprotein K (Fragment) GN=Hnrnpk
Q8K0D0	3	1	15	0.012262	0.340111	55.3	IMT	CTRL	Cyclin-dependent kinase 17 GN=Cdk17
**Q8BW41**	**5**	**3**	**26.1**	**0.011797**	**0.340111**	**49.1**	**IMT**	**CTRL**	**Protein O-linked-mannose beta-1_4-N-acetylglucosaminyltransferase 2 GN=Pomgnt2**
**A2A863**	**6**	**3**	**33.6**	**0.033583**	**0.497715**	**45**	**IMT**	**CTRL**	**Integrin beta-4 GN=Itgb4**
B7ZN94	1	1	4.8	0.041863	0.542228	43.1	IMT	CTRL	Fbxw14 protein GN=Fbxw14
A0A0A0MQ88	2	1	12.1	0.019122	0.398448	34	IMT	CTRL	Cytoplasmic phosphatidylinositol transfer protein 1 GN=Pitpnc1
B7ZP28	11	1	76.6	0.041194	0.542228	33.5	IMT	CTRL	Dennd2a protein GN=Dennd2a
**Q8BNG3**	**4**	**2**	**13.7**	**0.001197**	**0.099291**	**31.3**	**IMT**	**CTRL**	**Kinesin-like protein GN=Kif6**
A0A0U1RQ14	3	1	14.5	0.003354	0.185432	28.8	IMT	CTRL	MICOS complex subunit MIC60 (Fragment) GN=Immt
M0QWN7	4	1	26.3	0.002478	0.15224	25.5	IMT	CTRL	Ubiquitin-specific peptidase 35 GN=Usp35
D3Z5M8	6	1	37.1	0.001155	0.099291	24.4	CTRL	IMT	Fibroblast growth factor receptor 2 (Fragment) GN=Fgfr2
B7ZND5	4	1	23.2	0.001884	0.134404	22.6	IMT	CTRL	P2X purinoceptor GN=P2rx5
Q3TTV6	2	1	10.3	8.32E-06	0.00345	21.8	CTRL	IMT	Tuftelin-interacting protein 11 GN=Tfip11
**A2AST1**	**17**	**6**	**110**	**0.000258**	**0.040724**	**20.8**	**IMT**	**CTRL**	**Coiled-coil domain-containing 141 GN=Ccdc141**
E9PWW9	14	1	81.9	0.012912	0.345427	19.2	IMT	CTRL	Remodeling and spacing factor 1 GN=Rsf1
Q04692	4	1	21.6	0.001971	0.134404	17.5	CTRL	IMT	SWI/SNF-related matrix-associated actin-dependent regulator of chromatin subfamily A containing DEAD/H box 1 GN=Smarcad1

**E9QNV2**	**6**	**2**	**47**	**0.003956**	**0.199185**	**17.4**	**IMT**	**CTRL**	**Mediator of RNA polymerase II transcription subunit 23 GN=Med23**
A0A1S6GWI7	1	1	5.43	0.006758	0.261819	16.7	IMT	CTRL	Uncharacterized protein GN=Bud23
**Q9R1L5**	**3**	**2**	**13**	**0.008717**	**0.301232**	**16.3**	**IMT**	**CTRL**	**Microtubule-associated serine/threonine-protein kinase 1 GN=Mast1**
Q14B48	6	1	39	0.006859	0.261819	16	IMT	CTRL	Protein ITPRID1 GN=Itprid1
D3YUH8	3	1	16.1	6.39E-06	0.003027	15.3	IMT	CTRL	Rho GTPase-activating protein 20 GN=Arhgap20
A0A1Y7VM45	2	1	15.7	0.024742	0.430826	14.4	CTRL	IMT	Pre-mRNA-processing factor 39 (Fragment) GN=Prpf39
O88194	9	1	55.7	0.011473	0.336812	14.3	IMT	CTRL	Cadherin-related neural recepter 7 (Fragment) GN=CNR7
B9EIC2	3	1	15.7	0.03507	0.503642	13.6	IMT	CTRL	Nlrp4e protein GN=Nlrp4e
A0A1B0GQZ1	2	1	9.92	0.013934	0.356679	13.5	CTRL	IMT	39S ribosomal protein L23_ mitochondrial GN=Mrpl23
C9K101	4	1	22	0.00024	0.039835	13.4	IMT	CTRL	Non-specific serine/threonine protein kinase GN=Mark1
Q3TSQ8	2	1	11.8	0.01483	0.356679	13.2	CTRL	IMT	Uncharacterized protein (Fragment) GN=Bcan
Q9JI78	4	1	20.9	0.011935	0.340111	13.2	CTRL	IMT	Peptide-N(4)-(N-acetyl-beta-glucosaminyl)asparagine amidase GN=Ngly1
A2ATY4	2	1	16	2.59E-05	0.007511	12.9	IMT	CTRL	Zinc finger and BTB domain-containing 34 GN=Zbtb34
**A0A0R4J0F6**	**24**	**9**	**171**	**0.002895**	**0.166647**	**12.3**	**IMT**	**CTRL**	**Cyclin-G-associated kinase GN=Gak**
A2A9K7	7	1	46.1	0.018134	0.393185	12	CTRL	IMT	Connector enhancer of kinase suppressor of Ras 1 GN=Cnksr1
O35074	1	1	5.25	0.02759	0.459298	11.9	IMT	CTRL	Prostacyclin synthase GN=Ptgis
A0A1W2P7C0	6	1	34.5	0.026873	0.450243	11.7	CTRL	IMT	Utrophin GN=Utrn
A0A0G2JE49	1	1	5.36	0.020849	0.40704	11.5	IMT	CTRL	Paired immunoglobulin-like type 2 receptor alpha (Fragment) GN=Pilra
Q9DA97	4	1	19.7	0.003799	0.199185	11.3	CTRL	IMT	Septin-14 GN=Sept14
**D3YVV0**	**5**	**2**	**29.4**	**0.008072**	**0.289623**	**11.3**	**CTRL**	**IMT**	**Tetratricopeptide repeat domain 13 GN=Ttc13**
Q8BIM8	8	1	64.1	0.001603	0.123143	10.8	CTRL	IMT	Uncharacterized protein GN=Myh9
Q9WVM1	3	1	17.2	0.024629	0.430826	10.7	CTRL	IMT	Rac GTPase-activating protein 1 GN=Racgap1
G5E867	7	1	37.9	3.1E-07	0.000343	10.7	IMT	CTRL	Ras association (RalGDS/AF-6) and pleckstrin homology domains 1 GN=Raph1
A0A0G2JGU6	1	1	5.5	0.000192	0.033551	10.7	IMT	CTRL	T cell receptor alpha variable 15N-1 (Fragment) GN=Trav15n-1
Q6PGF5	3	1	15.7	0.019218	0.398448	10.6	CTRL	IMT	BMS1 homolog_ ribosome assembly protein (Yeast) GN=Bms1
Q9R1S7	2	1	9.37	0.032044	0.492698	10.4	CTRL	IMT	Multidrug resistance-associated protein 6 GN=Abcc6
A0A1L1SQR4	6	1	37	0.03148	0.492698	10.3	IMT	CTRL	Dedicator of cytokinesis protein 6 GN=Dock6
Q3TX47	66	1	761	0.009966	0.315236	10.2	CTRL	IMT	Tr-type G domain-containing protein GN=Eef2
Q05BH6	21	1	125	0.02386	0.427858	10.1	IMT	CTRL	Tcof1 protein (Fragment) GN=Tcof1

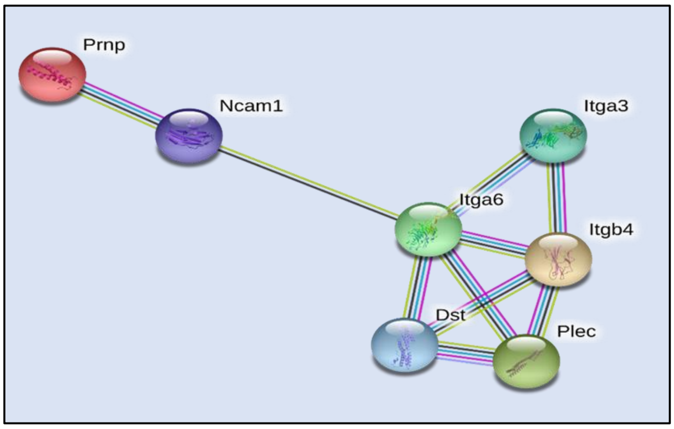

Figure S4: Protein-protein interaction and Classification of the identified allergenic gene following indirect microglial treatment (IMT). Protein-protein interaction analysis was performed using STRING v11.0 server. The *Mus Musculus* database was used as the host organism for the identification of protein interactions. Interaction among the allergenic protein (ITGB4) and PrP^C following indirect microglial treatment.

Table S9: Protein list identified following analysis of the 50 kDa band corresponding to FcεR1a using liquid-chromatography-mass spectrometry. Green color indicates the allergy related proteins identified by AllerGAtlas database.

P05213	Tubulin alpha-1B chain OS=Mus musculus OX=10090 GN=Tuba1b PE=1 SV=2
H9KV07	Nephrocystin-3 OS=Mus musculus OX=10090 GN=Nphp3 PE=1 SV=2
Q53D70	ZFP28 (Fragment) OS=Mus musculus OX=10090 GN=Zfp28 PE=2 SV=1
Q641M0	Uncharacterized protein OS=Mus musculus OX=10090 GN=2610008E11Rik PE=2 SV=1
F7APL9	Zinc finger protein 872 OS=Mus musculus OX=10090 GN=Zfp872 PE=4 SV=1
E9PZ68	Zinc finger protein 788 OS=Mus musculus OX=10090 GN=Zfp788 PE=4 SV=1
Q3URY5	Zinc finger protein 941 OS=Mus musculus OX=10090 GN=Zfp941 PE=1 SV=1
Q3V232	Uncharacterized protein OS=Mus musculus OX=10090 GN=Zfp867 PE=2 SV=1
G3X9T2	Zinc finger protein 619 OS=Mus musculus OX=10090 GN=Zfp619 PE=4 SV=1
**A0A3B2W7W2**	**Peroxisome proliferator-activated receptor delta OS=Mus musculus OX=10090 GN=Ppard PE=1 SV=1**
Q6A022	MKIAA0668 protein (Fragment) OS=Mus musculus OX=10090 GN=mKIAA0668 PE=4 SV=1
Q9JL35	High mobility group nucleosome-binding domain-containing protein 5 OS=Mus musculus OX=10090 GN=Hmgn5 PE=1 SV=2
